# Precisely measured protein lifetimes in the mouse brain reveal differences across tissues and subcellular fractions

**DOI:** 10.1038/s41467-018-06519-0

**Published:** 2018-10-12

**Authors:** Eugenio F. Fornasiero, Sunit Mandad, Hanna Wildhagen, Mihai Alevra, Burkhard Rammner, Sarva Keihani, Felipe Opazo, Inga Urban, Till Ischebeck, M. Sadman Sakib, Maryam K. Fard, Koray Kirli, Tonatiuh Pena Centeno, Ramon O. Vidal, Raza-Ur Rahman, Eva Benito, André Fischer, Sven Dennerlein, Peter Rehling, Ivo Feussner, Stefan Bonn, Mikael Simons, Henning Urlaub, Silvio O. Rizzoli

**Affiliations:** 1 Department of Neuro- and Sensory Physiology, University Medical Center Göttingen, Cluster of Excellence Nanoscale Microscopy and Molecular Physiology of the Brain, 37073 Göttingen, Germany; 2 Department of Clinical Chemistry, University Medical Center Göttingen, 37077 Göttingen, Germany; 3 Bioanalytical Mass Spectrometry Group, Max Planck Institute of Biophysical Chemistry, 37077 Göttingen, Germany; 4 Center for Biostructural Imaging of Neurodegeneration (BIN), 37075 Göttingen, Germany; 5 Genes and Behavior Department, Max Planck Institute of Biophysical Chemistry, 37073 Göttingen, Germany; 6 Department of Plant Biochemistry, Albrecht-von-Haller-Institute, Georg-August-University, 37073 Göttingen, Germany; 7 Laboratory of Epigenetics in Neurodegenerative Diseases, German Center for Neurodegenerative Diseases (DZNE), 37075 Göttingen, Germany; 8 German Center for Neurodegenerative Disease (DZNE), 81377 Munich, Germany; 9 Department of Cellular Logistics, Max Planck Institute for Biophysical Chemistry, 37077 Göttingen, Germany; 10 Laboratory of Computational Systems Biology, German Center for Neurodegenerative Diseases (DZNE), 37075 Göttingen, Germany; 11 Department of Psychiatry and Psychotherapy, University Medical Center Göttingen, 37075 Göttingen, Germany; 12 Department of Cellular Biochemistry, University Medical Center Göttingen, 37077 Göttingen, Germany; 13 Max Planck Institute for Biophysical Chemistry, Göttingen, 37077 Germany; 14 Institute of Medical Systems Biology, Center for Molecular Neurobiology (ZMNH), University Medical Center Hamburg-Eppendorf (UKE), 20246 Hamburg, Germany; 15 German Center for Neurodegenerative Diseases (DZNE), 72076 Tübingen, Germany; 16 Munich Cluster for Systems Neurology (SyNergy), 81377 Munich, Germany; 17 Institute of Neuronal Cell Biology, Technical University Munich, 80805 Munich, Germany

**Keywords:** Proteomics, Molecular neuroscience, Homeostasis, Molecular medicine

## Abstract

The turnover of brain proteins is critical for organism survival, and its perturbations are linked to pathology. Nevertheless, protein lifetimes have been difficult to obtain in vivo. They are readily measured in vitro by feeding cells with isotopically labeled amino acids, followed by mass spectrometry analyses. In vivo proteins are generated from at least two sources: labeled amino acids from the diet, and non-labeled amino acids from the degradation of pre-existing proteins. This renders measurements difficult. Here we solved this problem rigorously with a workflow that combines mouse in vivo isotopic labeling, mass spectrometry, and mathematical modeling. We also established several independent approaches to test and validate the results. This enabled us to measure the accurate lifetimes of ~3500 brain proteins. The high precision of our data provided a large set of biologically significant observations, including pathway-, organelle-, organ-, or cell-specific effects, along with a comprehensive catalog of extremely long-lived proteins (ELLPs).

## Abstract (web summary)

Measuring precise protein turnover rates in animals is technically challenging at the proteomic level. Here, Fornasiero and colleagues use isotopic labeling with mass spectrometry and mathematical modeling to accurately determine protein lifetimes in the mouse brain

## Introduction

Aged proteins must be replaced by newly produced ones in a precisely coordinated fashion, to avoid the accumulation of damaged molecules, and to prevent over- or under-production. This process, termed protein turnover, can be reduced to one basic parameter, the protein lifetime, which is the average amount of time spent by each protein before its eventual degradation. We currently lack basic information on protein lifetimes in the native brain, and on how the lifetimes are regulated in different brain structures, cells, or subcellular locations.

The stability of proteins has been analyzed in a number of studies, both in vivo and in cell culture. Most proteins seem to be degraded on a scale from ~1 day to a few weeks. While the majority of the proteome is probably exchanged fast, a few unusually long-lived proteins have been found in the eye lens, in myelin, in nucleosomes and in nuclear pores from the brain^1–4^. Such proteins have been identified by feeding animals with isotopically labeled diets (i.e., pulsing the animals), followed by long chase periods, in the absence of the isotopically labeled diet. The proteins that remain isotopically labeled after the chase are then detected by mass spectrometry, and have been termed long-lived proteins (LLPs) or extremely long-lived proteins (ELLPs)^3^. Such findings are valuable, but only reveal a minority of unusually stable proteins. They give no information about the wide majority of the proteins, whose lifetimes remain unknown.

To address this, several studies attempted to measure lifetimes in the brain in an unbiased fashion, by pulsing the animals with isotopically labeled diets for several different lengths of time, and then measuring the results in mass spectrometry^5,6^. These studies relied on a diet composed of blue-green algae (*Spirulina*) grown on ^15^N-containing medium. This implies that multiple ^15^N-containing amino acids are introduced into the proteins, resulting in complex mass spectrometry analyses. Relatively few lifetimes have been obtained (1000 or lower), and their precision was later questioned.

Analyses in cell cultures have been easier to perform, and several studies have been already performed in primary cultured neurons^4,7–9^. Unfortunately, in spite of the elegant pulse- or pulse-chase designs of these studies, the resulting protein lifetimes hold only limited information for the in vivo situation. All lifetimes are fairly short in culture, and tend to conform to a Gaussian distribution, which does not enable strong discrimination between the lifetimes of proteins in different cell compartments or pathways (e.g., between pre- and postsynaptic proteins, or between synaptic and non-synaptic proteins).

Overall, although protein turnover has been linked to the regulation of synaptic function^4,10^ and of neuropathology development^11,12^, a global resource for the lifetimes of proteins in the brain is not currently available. One of the major problems that have impaired in vivo studies is that pulse-chase protocols with isotopic amino acids have been designed for in vitro studies. Here proteins are derived from only one pool of amino acids: those that are added (or replaced) into the cell medium. In vivo at least two pools need to be considered: the isotopically labeled pool from the diet, and the non-labeled pool resulting from the degradation of the animal’s pre-existing proteins. The second pool slowly becomes labeled over time, which increases the complexity of the modeling. Multi-compartment mathematical models have been attempted for animals fed with ^15^N-containing *Spirulina*^13^, but due to complexities in amino acid metabolism their applicability remains limited, and their precision is questionable, as mentioned above.

To address all of these issues we developed a rigorous approach to the analysis of mouse brain turnover. We pulsed the mice with the essential amino acid lysine containing stable ^13^C isotopes^14^, relying on a standard diet that is easily accepted by the animals (unlike *Spirulina* chow), followed by mass spectrometry analysis. We developed a mathematical model that solved the issue of amino acid pools for lysine, and we tested the results by multiple approaches, ranging from double pulse-chasing with amino acids carrying different isotopes, to the analysis of peptides containing multiple lysines. Importantly, we have also designed new approaches to independently verify the mathematical model, by measuring the amino acid pools directly via gas-chromatography mass spectrometry, and by measuring the isotopic labeling of proteins produced on cue in the brains of tamoxifen-inducible mice.

This enabled us to determine the lifetimes of more than 3500 proteins in the brain, focusing on the cortex and on the cerebellum, on isolated synapses, on purified synaptic vesicles, and also on mice that underwent a prolonged environmental enrichment protocol, which is known to enhance synaptic plasticity and cognition^15^. We also compared the brain turnover measurements with the measurement obtained from the heart and the leg muscle tissue. Finally, we measured protein and mRNA abundance values, determined by next-generation sequencing and quantitative iBAQ mass spectrometry^16^.

## Results

### A method for in vivo protein lifetime determination

**Fig. 1:**
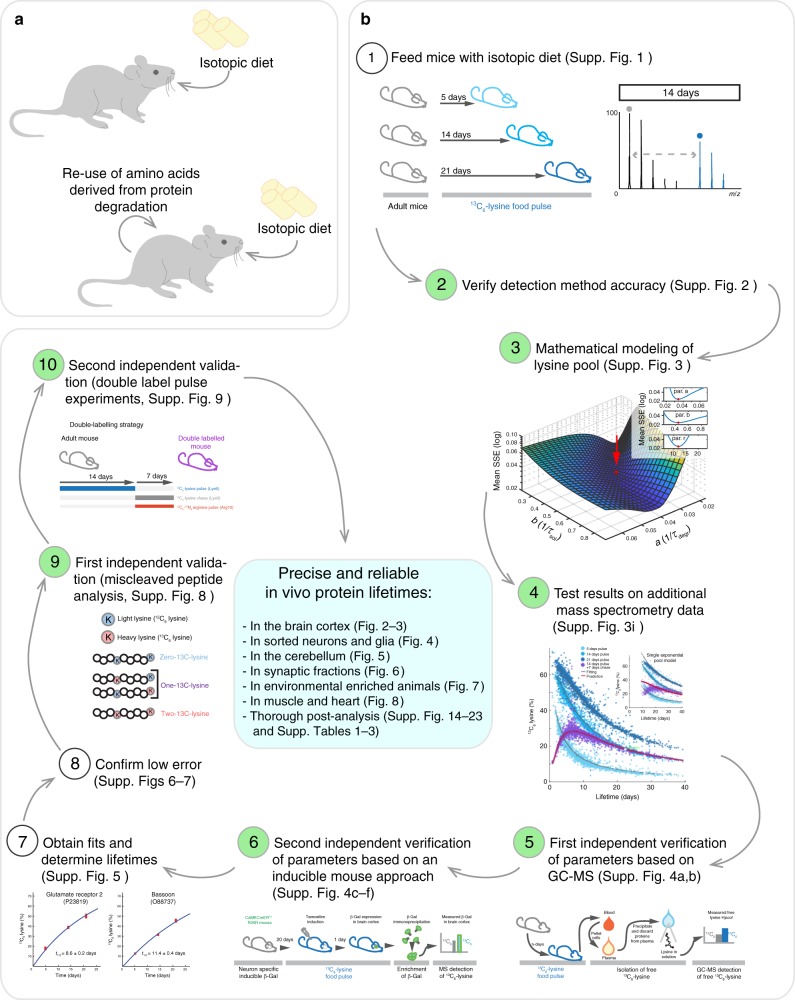
A highly calibrated method for determining protein lifetimes in vivo. **a** The interpretation of protein turnover in vivo is complicated by the initially non-labeled pool of amino acids that can be reused following the degradation of pre-existing proteins. **b** To solve this problem, a thorough protocol has been optimized and validated with several independent methods (as discussed in detail in the text and in Supplementary Figs. 1–9). Note that all steps highlighted in green have been performed for the first time in vivo

As indicated in the Introduction section, we argue that a major problem for in vivo isotopic pulsing is that for essential amino acids, two pools of amino acids need to be taken into account, the isotopically labeled one from the diet, and the initially non-labeled pool resulting from the degradation of the animal’s pre-existing proteins (Fig. 1a). We have optimized a thorough protocol to deal with this, which is explained in Supplementary Figs. 1–9, and is shown in graphical fashion in Fig. 1b. We are not aware of any comparable protocols in the literature, especially since 7 of its 10 main steps have never been performed in the past in vivo.

We have first optimized an in vivo metabolic pulse assay, as an extension of the stable isotope labeling with amino acids in cell culture (SILAC) technique^14^. We pulsed mice for three time periods (5, 14, or 21 days), by feeding them with a conventional SILAC diet (Supplementary Fig. 1a). In this diet, the essential amino acid lysine is substituted with an isotopically stable ^13^C_6_-lysine, which is an essential amino acid incorporated in newly produced proteins that can be detected by mass spectrometry (MS). The ^13^C_6_-lysine-containing proteins were produced at different rates (Supplementary Fig. 1b), suggesting that they turn over with different kinetics. This was not due to problems with peptide detection, since the detection of ^13^C_6_-lysine-containing proteins was as good as that of unlabeled proteins (Supplementary Fig. 2).

### A mathematical model of protein turnover in vivo

We then turned to an analysis of protein turnover in theoretical terms, considering the origin of the lysines used in protein synthesis. While the approaches used in vitro to calculate protein turnover can rely simply on a rapid substitution of the medium to easily determine protein half-life (*t*_1/2_; referred in this work as lifetime), the situation in vivo is more complex (Supplementary Fig. 3a, b). Animals absorb essential amino acids from the diet, but these can also be recycled following protein degradation, and re-enter the pool available for protein neo-synthesis (Supplementary Fig. 3c). As a consequence, in order to determine protein lifetimes in vivo it is necessary to study ^13^C_6_-lysine availability in the brain. Before the SILAC pulsing, the amino acids provided by protein degradation lack ^13^C_6_-lysine. During the pulse both ^13^C_6_-lysines (from SILAC food) and normal ^12^C_6_-lysines (recycled from degraded proteins) will be incorporated during the subsequent protein synthesis. To address this, we initially used a mathematical model, as detailed in Supplementary Fig. 3c (see also Methods). In order to account for the re-use of lysine from the degradation of proteins, we also included a pulse-chase approach, where we pulsed mice for two weeks and chased them for one week (Supplementary Fig. 3e). We adjusted the parameters of the lysine pool to obtain the best overall fit for all of the proteins measured by MS (Supplementary Fig. 3d–i). The optimized model indicates that ~50% of the lysines used in protein synthesis in the brain originate directly from the food intake, with the rest recycled through protein degradation. This model also predicts that the amount of lysines deriving from the food is rapidly saturated with ^13^C_6_-lysine (within ~1 day), while lysines derived from the degradation of previously existing proteins are exchanged far more slowly (Supplementary Fig. 3h). We tested the validity of this model by comparing it with a single exponential change of lysine availability, and we found that our model describes reliably ^13^C_6_-lysine availability in vivo in the brain (Supplementary Fig. 3i).

### Independent controls to test the mathematical model

To experimentally verify the model, we performed some initial controls in vivo. First, we measured the soluble ^13^C_6_-lysine pool, through a gas-chromatography MS approach applied on the mouse blood plasma, which should closely reflect the free soluble lysine pool that is available in the organism for protein synthesis. We found that that the content of ^13^C_6_-lysine in the plasma was indeed overlapping to that predicted by our model (Supplementary Fig. 4a, b). Second, to verify that actual proteins synthesized in the brain use this same lysine pool, we measured by a targeted MS approach the incorporation of lysines in an exogenous protein that was expressed on cue in the mouse cortex, using a tamoxifen-inducible system^17^. The amount of ^13^C_6_-lysine incorporated in this protein gives a direct measurement of the ^13^C_6_-lysine/^12^C_6_-lysine fractions that are available for protein synthesis. We found that ^13^C_6_-lysine incorporation was in good agreement with the one predicted by our model (Supplementary Fig. 4c–f). We then used the model to fit the different MS datasets (see examples in Supplementary Fig. 5). The model was able to fit the actual MS data with great precision (Supplementary Figs. 6–7), allowing us to calculate the protein lifetimes.

### Further controls to validate the mathematical model

Once we obtained the protein lifetimes, we designed some additional experiments to check the reliability of our measures, and performed some additional MS controls. First we checked that the lifetimes were in line with the labeling of peptides containing two lysines, by investigating the zero-, one- and two-13C-lysine versions of these peptides (Supplementary Fig. 8). Second, we tested with an in vivo double-labeling approach that the labeling of the peptides was in line with our predictions, relying on a 2-week pulse with ^13^C_6_-lysine, followed by a 1-week chase with ^13^C_6_-^15^N_4_-arginine (Supplementary Fig. 9). In both cases the model and the experimental measures were in good agreement. We also tested whether our dataset contained proteins whose stability diverged significantly from the model, relying on the chase data that was used for the precise estimation of the lysine pool (from Supplementary Fig. 3h, i). We found few significant results (Supplementary Fig. 10a, b and Supplementary Data 1), in line with the idea that non-exponential degradation in the proteome is a rare event, which is expected to affect <10% of proteins^18^.

### An overview of protein lifetimes in the brain cortex

**Fig. 2:**
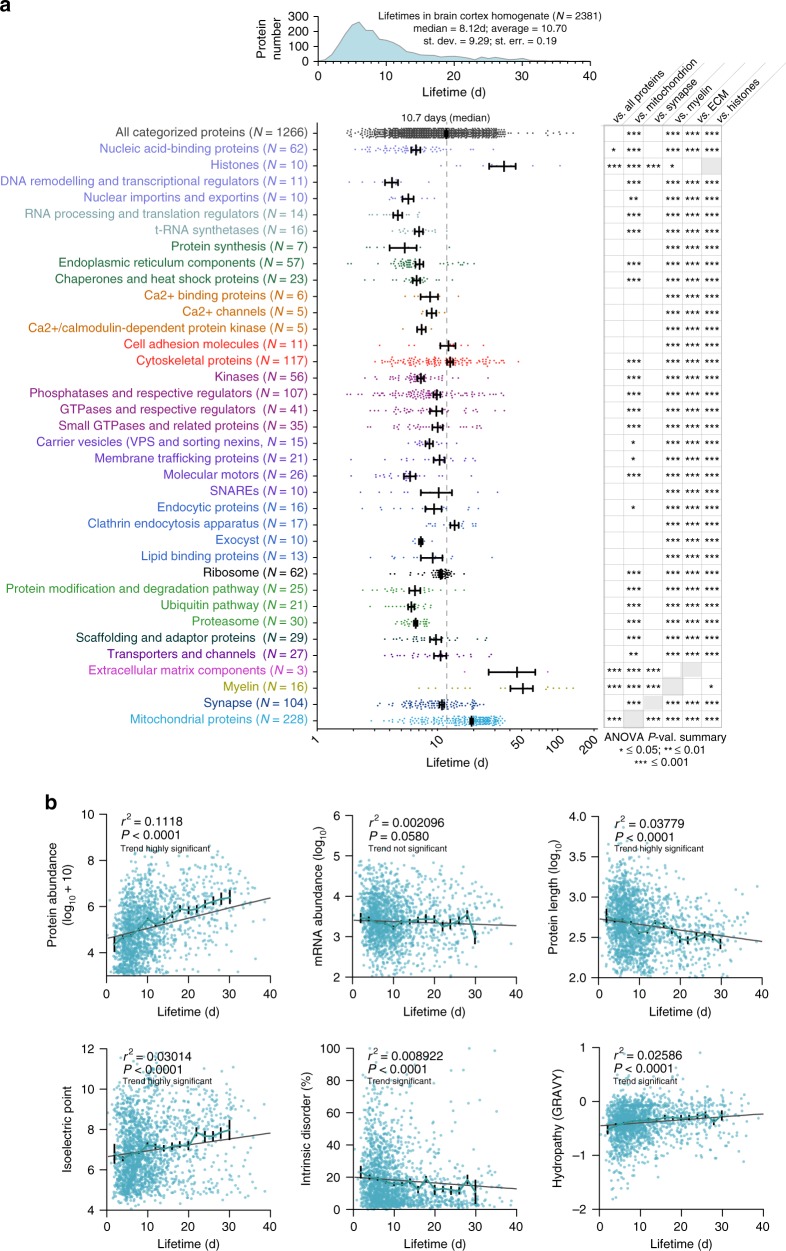
The lifetimes of brain proteins. **a** Upper panel: distribution of 2381 lifetimes calculated in the brain cortex homogenate. Lower panel: lifetimes of 1266 proteins organized in 36 groups, accordingly to their organelle and/or functional affiliation, performed by integrating previously published categorizations^19–24^ (see also Supplementary Data 1). Each data point corresponds to a single protein lifetime. The black lines indicate the mean and the standard error of the mean (SEM) for each group. The analysis of variance (ANOVA) on the right summarizes *P*-values for the indicated comparisons, following Bonferroni post hoc test (*≤0.05, **≤0.01, ***≤0.001). **b** Lifetimes are positively correlated to protein abundances (determined with iBAQ), isoelectric point, and grand average of hydropathy (GRAVY). Lifetimes are negatively correlated to protein length and intrinsic disorder, while the correlation to mRNA abundances is not significant. The lines with error bars indicate averaged bins of 2 days, with SEM. Brain cortex homogenate data have been used in this figure and the protein and mRNA abundances were measured in this study, in the same preparation (brain cortex of the same mice)

**Fig. 3:**
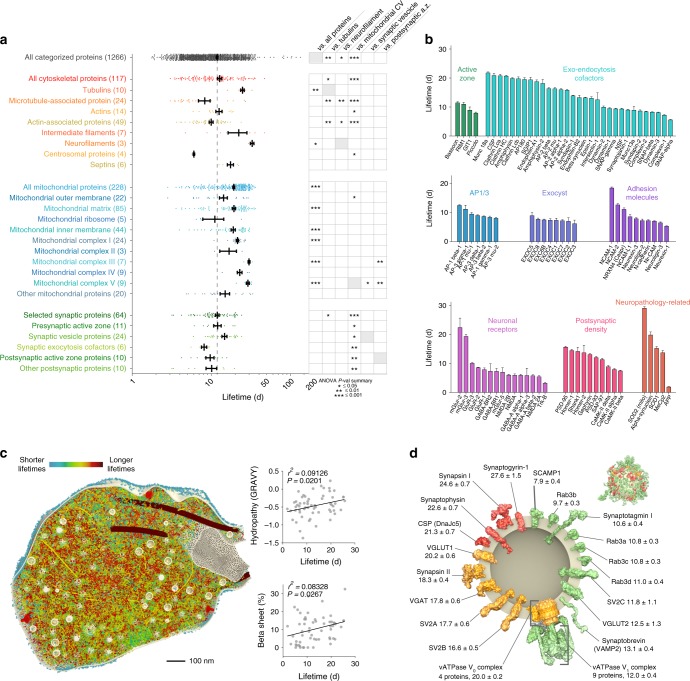
Detailed lifetimes of cytoskeletal, mitochondrial and synaptic proteins. **a** A subdivision of lifetimes for cytoskeletal, mitochondrial, and synaptic proteins subgroups^19–24^. The analysis of variance (ANOVA) on the right summarizes *P*-values for the indicated comparisons, following Bonferroni post hoc test (*≤0.05, **≤0.01, ***≤0.001). **b** The lifetimes of 90 proteins of particular interest, subdivided in 8 groups, are detailed. For each protein, the 95% confidence interval is reported as a measure of the error. **c** A section through a 3D model of the synaptic bouton, indicating 60 proteins shown in the copy numbers (as in ref. ^23^) and pseudo-colored as determined by their lifetime values. The two insets on the right side represent scatter plots of the lifetimes vs. either the hydropathy (upper panel) or the percentage of beta sheet stretches in the secondary protein structure (lower panel). In both cases there is a positive correlation between lifetimes and these values, indicating that hydrophobic and more structured proteins tend to live longer in the presynaptic bouton (ANOVA *P*-values ≤ 0.05). **d** A 3D view of a modeled synaptic vesicle, showing the lifetimes of 20 proteins (or protein complexes), as determined from the synaptic vesicle fraction of the brain cortex, see also Fig. 6. The proteins are color-coded from short-lived (green) to long-lived (red). The numbers indicate the lifetimes, in days and the error corresponds to the 95% confidence interval. The inset in the upper right corner is a synaptic vesicle, with proteins color coded for their lifetimes and shown in the appropriate copy numbers^23^. For the synaptic vesicle the data from the cortex subcellular fractionation has been used (see also Fig. 6), while all other data is from brain cortex homogenate

Having verified the validity of our lifetime measurements, we then studied the distribution of our results, first focusing on the lifetimes of proteins from the mouse cortex, which spread from ~1 day to several hundred days, albeit most were between 3 and 13 days (Fig. 2 and Supplementary Data 1). We first categorized the lifetimes of ~1200 proteins by integrating previously published databases of protein organelle location and functional affiliation^19–24^ (Fig. 2a and Supplementary Data 1). Proteins transiently binding to DNA, and several proteins related to signaling, RNA processing and protein production were among the shortest-living proteins, while histones, myelin components and extracellular matrix constituents were significantly longer lived than the average of all proteins. Since protein stability parameters have been previously linked to biophysical protein measurements and to protein and mRNA abundances^25–32^, we checked the correlation of our dataset with these values, which we either estimated with bioinformatics tools or we determined by iBAQ MS^16^ and next generation sequencing. Although all of these measurements correlated in a highly significant fashion with the lifetimes (with the exception of the mRNA abundance), the coefficients of determination are very modest (Fig. 2b), hinting at a related but different regulation of, e.g., protein abundance and protein lifetime. The lifetimes of proteins from the same organelle, family or protein complex were also correlated, as suggested by the lifetimes of protein from different organelles (Fig. 3) and by a thorough analysis of different protein complexes and families (Supplementary Fig. 10c–e).

### Comparison to previous studies

We have compared our data first with the largest data set of brain lifetimes available, from the seminal study of Price and collaborators^5^. Overall, our results correlate reasonably to the previous values, but several hundred of the previously published lifetimes seem to have been derived from less precise fits based on a single peptide measurements (see Supplementary Figs. 11, 12). We have also compared our data to those from four studies of primary cultured neurons in vitro^4,7–9^ (Supplementary Fig. 13). In brief, protein lifetimes in cultured neurons correlate with those in vivo, albeit not extremely well. The main difference lays in the fact that lifetimes in vitro are far shorter and are clustered together much more closely, which obscures many biologically relevant differences. For example, synaptic vesicle proteins tend to have about the same lifetime in vitro, but in vivo they separate into different sets of lifetimes (Supplementary Fig. 13d, e).

### Protein lifetimes in different organelles

We followed this up with an analysis of the cytoskeletal, mitochondrial and synaptic proteins (Fig. 3a). This revealed several interesting effects, such an unusually long lifetime of neurofilaments, or of mitochondrial proteins belonging to some of the oxidation chain complexes (Complexes V and III). Mitochondria proteins, albeit long-lived as a group, were fairly heterogeneous. The short-lived proteins in the mitochondrion tended to be either implicated in fatty acid metabolism (KEGG pathway mmu01212; false discovery rate, FDR, <0.001), or were connected with peroxisome function (KEGG pathway mmu04146; FDR < 0.001). We also found some notable differences in synapses. For example, the endocytosis machinery was very long-lived (Fig. 3b). Among synaptic adhesion molecules NCAM-1 was particularly long-lived (~20d), while several neurexins and neuroligins are turned over almost at twice the speed (with a lifetime of ~10d; Fig. 3b). Among neuronal receptors, some metabotropic receptors are almost twice as long-lived as the ionotropic ones (as an example mGlur-2 has a lifetime of ~21d while GluRs have a lifetime of ~9–10d). Interestingly, the structural components and the proteins with higher hydropathy (such as transmembrane proteins) were the most stable components of the synapse (Fig. 3c). At the level of the synaptic vesicle, the accessory proteins tended to be short-lived, while the core proteins are were longer-lived, on average (Fig. 3d).

To categorize protein lifetimes in a complementary unbiased approach, we used a fuzzy c-means clustering algorithm, coupled to a functional enrichment^33^ analysis of the identified proteins in non-redundant gene ontology (GO)-enriched terms for biological process, cellular component and molecular function categories (Supplementary Fig. 14). This revealed, for example, that the shortest lifetime group in the cortex is enriched for proteins involved in the regulation of RNA splicing and helicase activity, while clusters of stable proteins correspond to myelin, mitochondria, and structurally stable elements such as the cytoskeleton and nuclear pore.

### ELLPs and LLPs

Due to their peculiarity and low number, the most stable proteins in the proteome have been the focus of several reports^2–4^. While our pulse scheme was designed to cover in greater detail the lifetime of the majority of the proteome, we argued that some exceptionally LLPs might not be efficiently labeled at 21 days to allow the determination of their lifetimes. For this reason, we included two additional long-pulsing steps (30 and 60 days), specifically designed for the analysis of long-lived proteins. This allowed us to confirm and extend the list of previously defined ELLPs, while also providing their lifetimes for mice (Supplementary Table 1). To formally define ELLPs, we turned to a statistical description of the protein lifetime distribution. Lifetimes in different branches of science and industry have been analyzed for decades relying on the Weibull distribution (e.g., refs. ^34,35^). A Weibull distribution fit overlapped well with the distribution of protein lifetimes in the cortex (*r*^2^ = 0.93) for most of the lifetimes, but not above ~20–25 days. The fit suggested that <0.5% of the proteins are expected to have lifetimes greater than ~26 days, while in reality ~5% of the proteins have such lifetimes (10-fold more than expected). Moreover, the Weibull fit was virtually at 0 above lifetimes of ~33 days (0.02% of the proteins), although such lifetimes still accounted for ~2% of all proteins (100-fold more than expected). We have therefore formally defined ELLPs as the proteins that were in the upper 98th percentile of the proteome in term of stability. This list overlaps well with previous results^3^ and includes nuclear proteins (such as Histones and NUPs), myelin components, extracellular matrix proteins, and some other specific proteins (Supplementary Fig. 15). Following the results of the Weibull fit, we have also defined as “normal” LLPs those comprised from the 95th to the 98th percentile of the proteome in terms of stability. This group was mostly populated by mitochondrial proteins, enriched for the inner matrix and for some oxidation chain complexes (Supplementary Fig. 16), but contained also a number of signaling proteins and the neuronal exocytosis SNARE syntaxin-1A (Supplementary Table 1).

### Correlations of lifetimes to protein and mRNA abundances

We also evaluated the potential relations between protein lifetimes and protein and mRNA abundance, to obtain a global overview of protein homeostasis. For this, we relied on the data presented in Fig. 2b, and we subdivided the 3D scatter of the three datasets in 27 bins, as described in Supplementary Fig. 17. This analysis revealed a number of interesting protein classes. These include proteins with a short lifetime and low protein abundance, but with a relatively high mRNA abundance, which belong to regulatory processes such as protein polyubiquitination, response to radiation and to lipid changes (Supplementary Fig. 17c). At the same time, there are several processes that rely on proteins with long lifetimes, and large mRNA and protein abundances. These include many mitochondrial and cytoskeletal pathways.

### Lifetimes across cell types, brain regions, and fractions

**Fig. 4:**
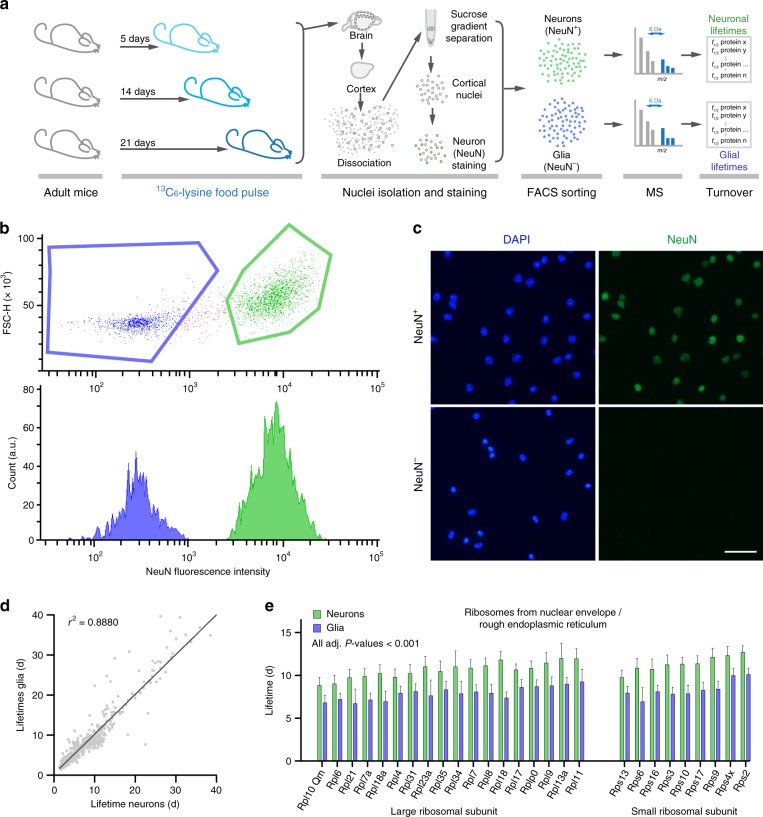
Lifetimes of proteins from sorted neuronal and glia cell nuclei. **a** Schematic representation of the pulsing strategy, followed by fluorescence-activated cell sorting (FACS), mass spectrometry analysis, and lifetime determination. **b** Representative scatter plot of sorting events, with the positive (green) and negative (blue) sorted populations highlighted vs. the forward scatter height (FSC-H). **c** Representative images of sorted neuronal (NeuN^+^) and glial (NeuN^−^) nuclei. **d** Scatter plot of protein lifetimes for neuronal and in glial nuclei. **e** Detailed lifetimes for components of the large and of the small ribosomal subunit. All differences are significant, with a Bonferroni adjusted *P*-value < 0.001. In all cases the ribosomes that are enriched in the nuclear envelope/rough endoplasmic reticulum are shorter-lived in glial cells than in neurons. See also Supplementary Data 1 for a detailed list of protein lifetimes in the nuclei of neurons and glial cells. See also Supplementary Fig. 18 for the string analysis of proteins either significantly longer-lived in neurons or in glial cells. Ribosomal constituents, focal adhesions and nuclear parts are significantly longer-lived in neurons vs. glial cells. The lower false discovery rates (FDR; an adjusted form of *P*-value to account for false-positive hits) observed in glia cells indicate that overall there are fewer long-lived groups of proteins in these cells

To address differences between neurons and glia, we combined our metabolic pulsing scheme with the sorting of NeuN^+^ neuronal nuclei and NeuN^−^ glial nuclei, and we calculated protein lifetimes in neurons and in glial cells separately (Fig. 4). While the lifetimes were in good correspondence (*r*^2^ = 0.88; Fig. 4d, Supplementary Data 1), some protein groups were strikingly different, including several subunits of the ribosomes associated with the nuclear envelope (longer-lived in neurons; *P*-value < 0.001). We also performed a functional categorization^36^ aimed at revealing functional networks to the proteins that were either significantly longer lived in neuron or in glial nuclei (respectively Fig. 4e, and Supplementary Fig. 18). This analysis also suggested, among other results, that gene expression regulators are more stable in neuron than in glial nuclei, and that some components of the nucleoplasm are longer-lived in glia nuclei (as detailed in Supplementary Fig. 18 and Supplementary Data 1).

We also relied on published data on the categorization of proteins enriched in different cell subtypes in the brain^37^ or in GABAergic and glutamatergic neuron subtypes^38^ (Supplementary Fig. 19). Oligodendrocytes are the only cell type for which we found significantly longer-lived proteins in the brain. This is due to the fact that these cells produce myelin, which is extremely long-lived. After excluding the proteins that compose myelin, no statistical difference remained (Supplementary Fig. 19a). We found no overall difference of protein markers from GABAergic and glutamatergic neurons (Supplementary Fig. 19b).

**Fig. 5:**
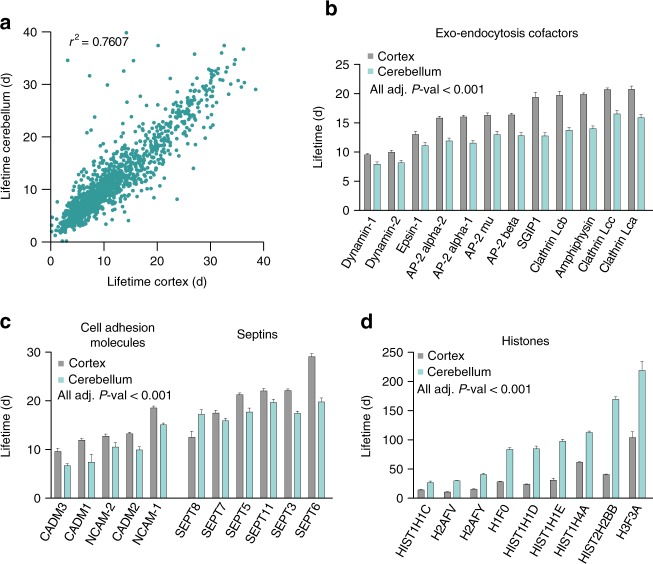
The lifetimes from specific pathways are different between brain cortex and cerebellum. **a** Scatter plot of protein lifetimes in the cortex homogenate vs. the cerebellum homogenate. **b**–**d** Proteins significantly different in the two tissues for several pathways identified by the classification of lifetime changes (Bonferroni adjusted *P*-value < 0.001; see also Supplementary Fig. 20). Several exo-endocytosis cofactors are shorter-lived in the cerebellum when compared to the cortex (**b**), as well as specific adhesion molecules of the brain (**c**, left side). With the exception of septin 8, most septins (3, 5, 6, 7, and 11) are also longer-lived in the cortex (**c**, right side). On the contrary, histones are more stabilized in the cerebellum than in the cortex (**d**)

We next analyzed the lifetime differences between the brain cortex and the cerebellum, two region of the brain characterized by very different cellular composition. While the lifetimes again correlated well between the two brain regions (*r*^2^ = 0.76; Fig. 5a, Supplementary Data 1), a categorization of the proteins in different classes (Supplementary Fig. 20a) revealed several highly significant differences. For example, several endocytic proteins and components of the clathrin endocytosis apparatus are shorter-lived in the cerebellum than in the cortex (Fig. 5b), as are many adhesion molecules (Fig. 5c). On the contrary, several histones are significantly longer-lived in the cerebellum, suggesting differential nucleosome stability between the two tissues (Fig. 5d). We also combined an unbiased functional analysis to understand if there were other processes differentially regulated in the two brain regions (Supplementary Fig. 20b). The analysis results confirmed our initial results, and also revealed that proteins implicated in RNA splicing are more stabilized in the cerebellum than in the brain cortex.

**Fig. 6:**
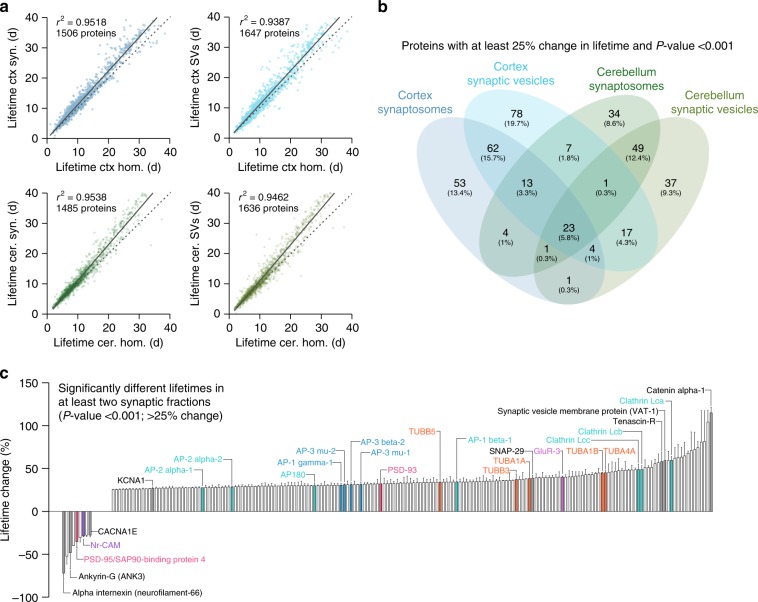
Protein lifetime changes in the synaptic fractions. **a** Scatter plots of protein lifetimes between the homogenate and either the synaptosomal or the synaptic vesicle (SV) fractions. The blue plots represent data obtained from the cortex, while the green plots correspond to the cerebellum. In all cases, synaptic-enriched fractions are longer lived, as indicated by the upward trend vs. the diagonal identity line (represented as a segmented trait). All lifetimes are reported with their confidence intervals in Supplementary Data 1. **b** Venn diagrams showing the overlap of proteins significantly changed in the four datasets (25% change with Bonferroni adjusted *P*-values < 0.001). While some proteins change specifically in only one dataset, there are many proteins that changed in the same manner across different datasets. **c** Average lifetime changes in the synaptic fractions (only shown for proteins significantly different in at least two of the four fractions, with a change >25% and *P*-value < 0.001). A positive change indicates longer lifetimes in the synaptic fractions. In accordance to the scatter plots, the majority of lifetimes are increased in the synaptic fractions. Synaptic molecules already discussed in Fig. 2 are detailed and color-coded as in Fig. 3b. Several exo-endocytosis cofactors (turquoise) are stabilized, together with AP-1/3 adaptor proteins (light blue). Several tubulin subunits are stabilized (orange), as well as GluR-3. A number of other synaptic components are indicated in black. In general there is a clear differential regulation of adhesion molecules, with some either stabilized or destabilized at the synapse, suggesting that their localization might be a predominant determinant of their stability. See also Supplementary Fig. 21c-f for the detailed gene ontology analysis of the lifetime changes among different fractions

To next test the relation between protein lifetimes and subcellular localization, we prepared synaptosomal and synaptic vesicle fractions from the cortex and from the cerebellum (Supplementary Fig. 21a), and measured the different lifetimes. In all cases the correlation between the lifetime of the total homogenate and the subcellular fractions was very good (average *r*^2^ = 0.95; Fig. 6a). The synaptic-enriched fractions were generally longer-lived (Fig. 6a–c). These results mirror those obtained in a recent study based on a single pulse and chase step^4^, in which synaptic proteins appeared to be longer-lived. However, the precision with which we can derive accurate lifetimes, coupled with confidence intervals, enabled us to determine that several proteins were significantly shorter-lived in synapses. These include proteins that have been linked to plasticity, and that are thought to be highly dynamic in synapses (Fig. 6c).

The proteins stabilized in synaptic fractions included several exo-endocytosis cofactors, tubulin subunits and adhesion molecules (Supplementary Fig. 21c–f). Interestingly, some proteins are stabilized in the synaptic fractions of the cerebellum (e.g. mitochondria matrix components), but not in those of the cortex (Supplementary Fig. 21d), and vice versa (e.g., microtubules in the cortex, Supplementary Fig. 21e).

### Protein lifetimes following physiological perturbation

**Fig. 7:**
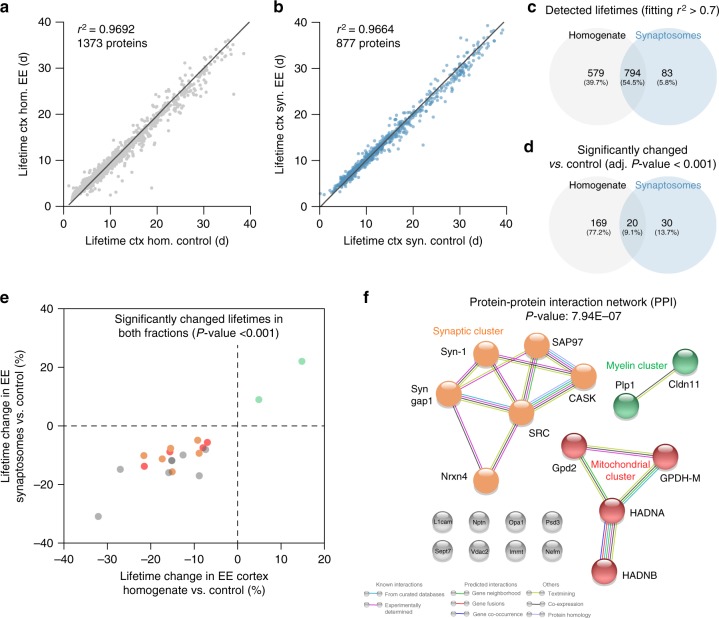
Protein lifetime differences upon chronic environmental enrichment. **a**, **b** Scatter plots of protein lifetimes upon prolonged environmental enrichment in the cortex homogenate (**a**) or in the cortex synaptosomes (**b**) vs. the control mice. **c** Among the proteins whose lifetime have been precisely determined, there are 794 common hits. **d** Some of the proteins are significantly different (Bonferroni adjusted *P*-value < 0.001), and of these only 20 are common among the two cellular fractions. **e** A precise analysis of these proteins identifies synaptic components that are turned over at a higher speed following environmental enrichment, such as the presynaptic adhesion molecule Neurexin-4, the scaffold molecule CASK, the phosphoprotein synapsin-1 and the neuronal RasGAP SynGAP1 (see Supplementary Table 2 for details). Some mitochondrial components implicated in the metabolism of glutamate and acetyl-CoA are also turned over at a higher speed following environmental enrichment (Supplementary Table 2). On the contrary, two myelin components (PLP1 and Claudin 11) are stabilized upon environmental enrichment (Supplementary Table 2). **f** String analysis of the 20 common proteins. This identifies three functional protein clusters changed upon environmental enrichment (Supplementary Table 2). The detailed analysis indicates that the most important differences between these two cohorts of mice are at the level of myelin, the mitochondrial inner membrane and the synapse

Having thus observed that both protein function and protein location influence the brain protein lifetimes, we turned to investigating how protein lifetimes change after physiological perturbation of brain activity (Fig. 7, Supplementary Table 2 and Supplementary Data 1). We therefore compared the lifetimes in the cortex with those of environmentally enriched (EE) mice, for both the overall brain homogenate and for the synaptosomal fraction. In both cases the correlation between datasets was very high (*r*^2^ = 0.97; Fig. 7a, b), albeit several proteins changed their lifetimes significantly (adjusted *P*-value < 0.001; Fig. 7c, d). Among the highly significant changes we identified mostly proteins that were shorter-lived in the environmentally enriched mice (Fig. 7e and Supplementary Table 2), with only two myelin proteins (PLP1 and Cldn11) being longer-lived in these mice. The shorter-lived proteins include some well-known synaptic proteins such as Synapsin-1, CASK, Syngap1, and Neurexin-4, and some mitochondrial proteins that are involved in glutamate metabolism (Fig. 7f). A gene ontology analysis of these groups confirmed their involvement in myelin formation, mitochondrial function and nervous system development (Supplementary Table 2).

These results are at odds with those obtained in a recent study based on a single pulse and chase step^4^, which suggested that virtually all proteins increased their turnover after the EE procedure. This result is difficult to accept, since such a massive change in brain turnover has never been reflected by any of the previous studies in the EE literature. We suggest that the apparently higher turnover is due to differences in the feeding rates. Mice subjected to EE run and move more, thus feed more. The amino acid pools change, due to the different feeding behavior, and thus proportionally more isotopically labeled amino acids become available for daily protein production than before. This in turn translates into different protein labeling, which will be interpreted as differential protein stability.

### Protein lifetimes in neuronal cultures and other organs

**Fig. 8:**
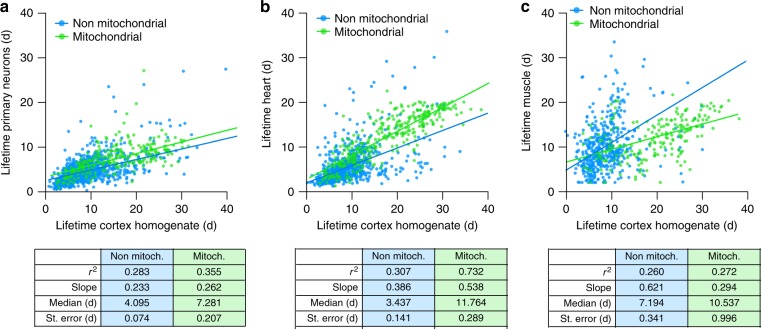
Protein lifetimes across different cells or tissues. **a**, **b** Scatter plots of the lifetimes in the cortex homogenate versus the lifetimes of primary rat neurons (published elsewhere^7^; **a**). Non-mitochondrial proteins are represented in blue while mitochondrial proteins are represented in green. The lifetimes of proteins in vitro are shorter than in vivo, both for mitochondrial and non-mitochondrial proteins. Even accounting for this overall difference, presynaptic proteins are shorter living in cultured cells than in vivo (see Supplementary Table 3 for details). This difference might be due to the fact that culture neurons are still growing and developing axons and synapses at the time of the measurements. **b** Scatter plots of the lifetimes in the cortex homogenate versus the lifetimes in heart samples. In the heart the turnover of proteins is overall faster than in the brain, but the mitochondria proteins tend to be longer-lived than the rest of the proteome. The gene ontology analysis suggests that the main lifetime differences observed between the two tissues are at the level of cell cycle and metabolic processes, in line with their different requirements of the two tissues (Supplementary Table 3). **c** Scatter plots of the lifetimes in the cortex homogenate versus the lifetimes of skeletal muscle (gastrocnemius). Protein turnover in muscle is also faster than in the brain. If compared with the heart, the mitochondrial turnover in the muscle is relatively faster, probably reflecting a difference in the metabolic requirements of the two tissues (please compare **b** and **c**). In the muscle the turnover of proteins involved in DNA biosynthesis is relatively faster than in the brain, probably because of the limited cell renewal and DNA synthesis observed in the nervous system (Supplementary Table 3)

Finally, we compared the protein lifetimes from the brain cortex with lifetimes measured in rat primary hippocampal neurons by Ziv and collaborators^7^ (taken as representative for the different in vitro studies, Supplementary Fig. 13) as well as with the protein lifetimes that we measured in the mouse heart and leg muscle (Fig. 8 and Supplementary Table 3). Overall, long-lived proteins in the cortex remained long-lived in these fractions, although the *r*^2^ values were on average lower (~0.28). A differential analysis revealed several interesting trends. For example, most proteins were shorter-lived in culture than in the brain, but presynaptic proteins were especially short-lived in cultured neurons, when compared to the brain. This difference might be due to the specific developmental pattern of neuronal cultures in vitro versus the brain cortex in vivo (Fig. 8a). As another example, the overall turnover of mitochondrial proteins in the heart is similar to that in the brain, while this is not the case for most other proteins, which are shorter-lived in the heart (Fig. 8b). A gene ontology analysis revealed that the proteins involved in the cell cycle are also differentially regulated (Supplementary Table 3). The muscle turnover also showed a large difference between mitochondrial and non-mitochondrial proteins (Fig. 8c), while also revealing a faster turnover than in the brain for proteins such as those implicated in the biosynthesis of DNA, probably because of the limited cell renewal observed in the nervous system (Supplementary Table 3).

### Lifetime correlations point to similarly regulated proteins

A potential use of our data is the investigation of homeostasis links between proteins. We have argued that, since protein lifetimes are correlated to their functional interactions, and since different protein complexes and pathways are differentially used across tissues, the variation in protein lifetimes across tissues may reveal functionally or homeostatically related groups of proteins. To test this, we studied the correlation of ~450 proteins whose lifetimes were measured in seven different tissues or cellular compartments (cerebellum and cortex homogenates, synapse fractions and synaptic vesicle fractions, along with heart samples). The overall changes across tissues and fractions were positively correlated for all of the ~450 proteins, when taken as a whole (correlation coefficient of ~0.58). Nevertheless, the changes in the lifetimes of members of several known functional complexes correlated very strongly (correlation coefficients higher than 0.9; Supplementary Fig. 22). Proteins that are not expected to function together, such as the different annexins, did not correlate (Supplementary Fig. 22c). In contrast, proteins known to function together, such as those involved in vesicle endocytosis, correlated strongly. While considering proteins whose functional interactions are unclear, we observed an extremely high correlation between several Rab proteins^39^, between two Vps proteins (which have been suggested to form a sub-complex^40^), or between several Arp proteins^41^. This suggests that there may be coordinated functional and/or homeostatic interactions between these molecules. This kind of analysis could serve as a guide for the development of new hypotheses on protein interactions, which would then be tested by independent experiments.

### Comparing in vivo lifetimes with in vitro manipulations

At the same time, our data can be employed to study the relation of protein lifetimes to the results of various cellular manipulations. More generally, it could be the basis for works that mechanistically dissect how different protein production and degradation forces shape the proteome, by integrating our in vivo lifetime analysis with in vitro results on manipulations of neuronal cultures. To showcase this possibility, we have considered some recently published results^42^ on the deceleration of protein degradation measured in rat in vitro neuronal cultures. We have compared our lifetime data with these results, and we could confirm the idea that shorter-lived proteins are those first to be increased in amounts following proteasome inhibition (Supplementary Fig. 23a). We could confirm this also in our own experiments (Supplementary Fig. 23b, c). Conversely, inhibiting protein translation by Cycloheximide decreased the level of short-living proteins, while longer-living proteins were apparently increased (Supplementary Fig. 23d). Similar approaches could potentially be extended to other drug treatments (e.g., lysosome inhibition), to decipher the links between protein lifetimes and various turnover pathways.

### Comparing in vivo lifetimes with protein surfaces

Protein lifetimes could also be correlated to other types of parameters, to reveal the regulatory mechanisms that are at the basis of protein turnover. One simple hypothesis that we have explored is if the size of the exposed surfaces of proteins can play a role in their stability, also in the light of the observation that more structured membrane proteins are more stable at the synapse (Fig. 3c). To do that, we analyzed the turnover of 72 proteins or protein complexes from the presynaptic compartment, whose surfaces we have analyzed in the past^23^, and found that the size of the surface exposed to the cytosol was negatively correlated to the lifetime (Supplementary Fig. 23e), in line with the observation that large proteins tend to be shorter-lived (Fig. 2b).

## Discussion

In this study we examined the proteome turnover in several samples, encompassing different organs, tissues, cells, organelle fractions, and activity modulations. Our work constitutes the largest available dataset of protein lifetimes in the nervous system, complementing and substantially improving the knowledge accumulated by previous protein turnover studies^2–5^.

We have developed here, an in vivo pulsing strategy workflow that utilizes isotopically stable ^13^C_6_-lysine, coupled to a proteome-wide mathematical description of lysine metabolism. This strategy brings several improvements for the determination of lifetimes, including a simple and robust interpretation of mass spectrometry results, which allows obtaining data for several peptides for each protein. This is a major advantage with respect to approaches based on ^15^N diets that limit the analysis to fewer arbitrarily selected peptides^5^, thereby rendering our lifetime determinations much more accurate. The development of this strategy also gave us the chance to clarify some general aspects of essential amino acid re-usage, with potential implication for other metabolic studies and possibly opening new avenues for similar studies.

At the same time, we introduced four different ways of testing the accuracy of in vivo results: double-labeling of mice with amino acids containing different isotopes, an analysis of peptides containing multiple isotopically labeled amino acids (e.g., peptides with two lysines), a direct analysis of the amino acid pool by gas chromatography mass spectrometry (GC-MS), and a direct analysis of a protein expressed on cue in the brain of isotopically pulsed mice. This is a new toolbox for in vivo mass spectrometry, whose applications reach far beyond this study.

The overall description of how the proteome is exchanged over time indicates that in the brain a large majority of proteins have lifetimes between 3 and 13 days. While our approach was tuned to have the best dynamic range in this short period, we could also confirm and expand the previous categorization of ELLPs. Due to their peculiarity these stable proteins have been the focus of several studies^2,3^, including a very recent work^4^. For example, it is appealing to speculate that the ELLPs are somehow more relevant than other proteins, since some may be long-term carriers of positional and functional information within the brain^43^. At the same time, the definition of the “extremely stable proteins” is somehow arbitrary when performed without exact knowledge of the other protein lifetimes. For example, our large collection of lifetimes allowed us to distinguish between LLPs (roughly between the 95th and 98th percentile most stable proteins) and ELLPs (98th to 100th percentile most stable proteins), which we found to have different functional implications.

Similar analyses also provided several quantitative conclusions. For example, it is clear that protein lifetimes are conserved across different organs, albeit the overall proteome turnover can be faster or slower in each tissue (e.g., brain, heart, or muscle), to cope with their specific metabolic needs. At the same time, profound differences can be found at the level of particular pathways and organelles. These changes might reflect a distinct usage of these proteins in the different conditions, or more simply may represent an alternative regulation of their production/degradation rates. Independent from the cause of these changes, specific differences in the stability of proteins may be essential for understanding the function and regulation of the particular pathways. The cytoskeleton is a good example, since it contains molecules with apparently similar functions, but different lifetimes. Its most stable components are neurofilaments, followed by tubulins and intermediate filaments, while the lifetime of actins is very close to the average lifetime of the entire proteome (Fig. 3a). Similarly, proteins associated to microtubules are more stable than proteins associated to actin, along with the suggestions from the literature that the microtubule cytoskeleton is a more stable component of the cell than the highly dynamic actin network.

Our results on the subcellular fractionation indicate that localization to synapses and to synaptic vesicles extends the lifetime of most proteins, probably reflecting a differential regulation of protein degradation in these compartments. At the same time, the precise determination of lifetimes with confidence intervals allows us to pinpoint specific changes of several proteins that are especially stabilized or destabilized at the synapse. Among these, several adhesion molecules are more stable in synapses than elsewhere, potentially due to mechanisms that remove adhesion molecules if they are not engaged in synaptic contacts. Interestingly, among the most destabilized proteins in synapses there is the adhesion molecule Nr-CAM (along with its binding partner Ankyrin), which is implicated in directional signaling during axonal cone growth and synapse formation, and might be specifically removed from mature synapses.

We have alluded in the Introduction and in Results to several important previous studies dealing with protein stability or protein lifetimes. Our results complement the databases of long-lived proteins presented in previous works^2–4^. Importantly, our data provide a clearer perspective for such proteins, by comparing them accurately with normal proteins. For example, nuclear pore proteins or histones are well-known ELLPs, but many of them do not live far longer than, for example, the fusion SNARE protein Syntaxin 1A (Supplementary Table 1). At the same time, it is extremely interesting that SNAP25, the closest functional partner of Syntaxin 1A, only lives 4 days in vivo (8-fold shorter than Syntaxin 1A). In view of such data it is evident that a discussion of ELLPs alone, without information on the other proteins, has only a limited use.

Our data extend widely the previous lists of protein lifetimes in the brain^5,6^. Importantly, we also updated and rectified these lists, thereby removing much uncertainty from the field (Supplementary Fig. 11–12). We also extend and correct several aspects determined in vivo by single pulse and chase procedures^4^, including descriptions of protein lifetime differences between synapses and brain homogenates, or between normal and environmentally enriched mice.

Finally, our data offer a new perspective on protein lifetime determinations in vitro, in cultured neurons^4,7–9^. In spite of the excellent technical setups behind these experiments, which are aided by the ease with which cell cultures can be manipulated, the in vitro results are far more limited than the in vivo ones, due to the short lifetime of the cells. This implies that most protein lifetimes are short (Supplementary Fig. 13), and biologically interesting differences are far harder to detect than in vivo.

We therefore conclude that our data will serve as a useful resource for different studies, ranging from the modulation of proteome homeostasis and metabolism to the planning of down-regulation and inducible knockout approaches in vivo. At the same time, our method for determining precise lifetimes can be easily reproduced in different mouse models, and should therefore prove useful for the study of brain physiology and pathology.

## Methods

### Mice

All mouse experiments were approved by the local authority, the Lower Saxony State Office for Consumer Protection and Food Safety (Niedersächsisches Landesamt für Verbraucherschutz und Lebensmittelsicherheit). Adult (>3.5 months old) wild-type male mice (C57BL/6JRj) were purchased from Janvier Labs (Germany). The R26R LacZ reporter mouse line^44^ was crossed to the tamoxifen-inducible CaMKCreERT2 driver line expressing the CreER^T2^ fusion protein under the control of the regulatory elements of the CaMKIIα gene^17^. Expression was triggered by a Tamoxifen intraperitoneal injection at a dose of 2 mg dissolved in corn oil (Sigma) twice per day before brain harvesting and tissue processing. For the enriched environment (EE), mice were housed in large plastic cages in groups of 5–8 and provided with a series of toys. The latter consisted of running wheels, tubing, a housing item, as well as a variety of objects of different size and texture. To ensure novelty, at least two of the items, excluding the housing item and the running wheels, were exchanged for two novel objects every other day. The remaining toys were rearranged in the cage to ensure spatial novelty. Mice were enriched for 6 weeks before the beginning of the SILAC labeling and during the SILAC labeling. We have previously shown that this enrichment protocol results in enhanced memory function^45^.

### SILAC mouse labeling

The L-^12^C_6_-lysine, the L-^13^C_6_-lysine and the L-^13^C_6_–^15^N_4_-arginine SILAC diets^14^ were purchased from Silantes, Martinsried, Germany. For pulsing experiments mice were first habituated to the unlabeled L-^12^C_6_-lysine diet, before starting feeding the L-^13^C_6_-lysine diet. We performed this to avoid undesired effects, such as weight loss, which can occur due to the dietary switch. Several different labeling pulses have been used in this work. For the overall determination of protein lifetimes we relied on short pulses (0, 5, 14, and 21 days), which we estimated after a preliminary study to be the best pulsing times for picturing the large majority of the proteome (which has a median lifetime of 8.12 days and an average of 10.7 days). For the long-living proteins we added two additional longer pulses (30 and 60 days), to better picture the change in the most stable fraction of the proteome. We also included a pulse and chase approach where we pulsed for 14 days and we chased with the light L-^12^C_6_-lysine diet for 7 days (used for the estimation of the lysine pool in Supplementary Fig. 3 and for the determination of the more- or less-stable proteins in Supplementary Fig. 10). Finally we included a pulse and chase approach where the chase was concurrent with a second pulse, for the confirmation of the validity of our model (as detailed in Supplementary Fig. 9). All animals were fed ad libitum and had unrestricted access to water. Food consumption was monitored daily, to exclude mice that were not eating regularly from the study. The brain of the “full SILAC” mouse used in Supplementary Fig. 3 (>2 generations on L-^13^C_6_-lysine SILAC diet) was also purchased from Silantes.

### Determination of free ^13^C_6_ lysine in blood plasma

Blood serum was extracted with two volumes of extraction buffer (methanol/chloroform/water 32.25:12.5:6.25 [v/v/v]). The mixture was vortexed and centrifuged for 5 min at 2000 × *g* at 4 °C. A volume of 20 µl of the upper polar phase was dried under nitrogen stream, rendered volatile with methoxyimino (MEOX)- and trimethylsilyl (TMS) derivatives, and analyzed by GC-MS as recently described^46^. For quantification of lysine, the 4 TMS derivatives were analyzed. The ^12^C_6_ to ^13^C_6_ ratio of lysine was determined by quantifying the mass-to-charge ratios 317 Da/e and 322 Da/e. A total of 33 independent experimental measures were included in this experiment, corresponding to one animal each. The variation depends on the feeding behavior of each animal. This is not a problem in the MS determinations, where each biological sample corresponded to a pool of four animals (that was necessary to obtain sufficient material for the subcellular enrichment of synaptic fractions).

### Immuno-enrichment of β-galactosidase

Beta-galactosidase was enriched through immunoprecipitation with Anti-β-Galactosidase (Promega, Mannheim, Germany; mAb Z3781) and pull-down with Dynabeads Protein A (ThermoFisher Scientific, Germany). In brief, the mouse cortex was dissected, homogenized, sonicated and treated with Benzonase-nuclease (Sigma) to remove nucleic acids. For 20 mg of homogenate sample, 300 µl of Dynabeads Protein A were loaded with 70 µg of anti-β-galactosidase antibody, following the protocol of the producer.

### Preparation and characterization of brain fractions

To obtain enough material for fractionation, the tissues (cortex or cerebellum) from 4 mice were pooled for each biological replicate. Following brain extraction, brain fractions were purified from mouse cortex and cerebellum as described^22,47^. Briefly, after dissection the tissues were homogenized in ice-cold sucrose buffer (320 mM Sucrose, 5 mM HEPES, pH 7.4) with a glass-Teflon homogenizer at 900 rpm. Following the collection of sample of the homogenate, for further analysis, samples were subjected to a 2 min 1000 × *g* centrifugation (to discard large cellular debris, P1) and to a 12 min 15,000 × *g* centrifugation to pellet the P2 fraction (Supplementary Fig. 21a). To obtain axonal fractions, a further fractionation step was performed, by applying a step-gradient centrifugation (6, 9 and 13% Ficoll in sucrose). The two fractions at the two interfaces of the 9% Ficoll medium were pooled (in what is termed P2’), were washed in sucrose buffer, and were further processed for hypo-osmotic lysis. Upon lysis, synaptosomal membranes were pelleted for 15 min at 17,000 × *g*, and the supernatant was centrifuged for 2 h at 300,000 × *g*, to obtain the axonal (presynaptic) pellet (termed LS2). Immediately after purification, each fraction was snap frozen in liquid nitrogen and was stored at −80 °C until used in further experiments. The quality of brain fractions was confirmed by immunoblotting (Supplementary Fig. 21b) with the antibody listed in Supplementary Table 4.

### Mass spectrometry

The protein concentration of individual samples was determined with a BCA kit (ThermoFisher Scientific). For each sample, 100 µg of total protein was loaded on pre-casted NuPAGE gels (4–15%, ThermoFisher Scientific). Gels were run at constant voltage, stained overnight with Coomassie Blue, and were destained with water. After destaining, each lane was cut into 23 gel pieces using an in-house-made gel cutter, and processed for in-gel digestion using trypsin^48^ (Serva). The eluted peptides were dried and resuspended in 5% acetonitrile and 0.1% formic acid solution, and were further processed for LC-MS in an online UltiMate 3000 RSLCnano HPLC system (ThermoFisher Scientific) coupled online to the Q-Exactive-HF. Peptides were desalted on a reverse phase C18 pre-column (3 cm long, 100 μm inner diameter 360 μm outer diameter) for 3 min. After 3 min the pre-column was switched online with the analytical column (30 cm long, 75 μm inner diameter) prepared in-house using ReproSil-Pur C18 AQ 1.9 μm reversed phase resin. The peptides were separated with a linear gradient of 5–50% buffer B (80% acetonitrile and 0.1% formic acid) at flow rate of 10 nl/min over 88 min and 58 min gradient time. The temperature of the pre-column and of the column was set to 50 °C during chromatography. The MS data were acquired by scanning the precursors in mass range from 350 to 1600 Da at a resolution of 60,000 at *m*/*z* 200. The top 30 precursor ions were chosen for MS2 by using data-dependent acquisition (DDA) mode at a resolution of 15,000 at *m*/*z* 200 with maximum IT of 50 ms. For MS2, HCD fragmentation was performed with the AGC target fill value of 1e5 ions. The precursors were isolated with a window of 1.4 Da. The lock mass option (*m*/*z* 445.1200) was used for internal recalibration. For the experiment in Supplementary Fig. 2 the homogenate of the brain of the “full SILAC” mouse was mixed with normal brain homogenate, in-solution digested and processed for MS.

For the targeted detection of the β-galactosidase, the enriched protein was “on-bead” digested using Trypsin (Promega). Briefly, the beads were resuspended in 30 μl of 1% RapiGest (Waters) and heated to 95 °C for 5 min. Further, 20 μl of 55 mM dithiothretol was added and incubated for 1 h at 750 rpm at room temperature followed by addition of 20 μl of 100 mM iodoacetamide for 20 min, 750 rpm at room temperature in dark. After diluting the detergent concentration to 0.1%, 10 μl of 0.1 μg/μl Trypsin (Promega) was added and incubated overnight at 750 rpm, 37 °C. The trypsin was quenched by adding 20 μl of 10% Formic acid. The undigested peptides were pelleted while the supernatant was desalted and dried. The peptides were resuspended in 40 μl of 5% acetonitrile containing 0.1% formic acid. A volume of 5 μl of resuspended peptides were injected on a reverse phase C18 pre-column (3 cm long, 100 μm inner diameter 360 μm outer diameter) followed by analytical column (30 cm long, 75 μm inner diameter) prepared in-house using ReproSil-Pur C18 AQ 1.9 μm reversed phase resin. The peptides were separated with a linear gradient of 5–50% buffer B (80% acetonitrile and 0.1% formic acid) at flow rate of 10 nl/min over 58 min gradient time on a TSQ Vantage Triple Quadrupole Mass spectrometer for targeted MS.

### Mass spectrometry data analysis

The acquired RAW data was analyzed using MaxQuant software^49^ version 1.5.2.8 based on the Andromeda search engine^50^. The mouse UniProt database (downloaded on 2015.11; containing 16,727 reviewed entries) was used for identifying proteins. For defining the label on peptides, the multiplicity was selected to “2” and the label ^13^C_6_-lysine was ticked as heavy. Protein quantification was based on “unique and razor peptides” for a given protein. For each protein, at each one of the three time points (5, 14, and 21 days), three biological replicates and three technical replicates were measured (27 measurements in total for each type of sample, although not all proteins were always detected in all fractions; see Supplementary Figs. 6–7 for a detailed description of the error estimation in the calculation of the lifetimes). The median H/L (Heavy to Light) ratios among detected peptides (>3) was determined for each protein. Ratios were used for the determination of protein lifetimes, following the modeling of the amino acid pool, as described below. For the analysis of long-living proteins (Supplementary Table 1 and Figs. 15–16) we defined ad hoc a database of proteins that had longer lifetimes in our results, but we also included the previously described long-living proteins^2,3^ and the proteins that were identified only in the label-free fraction in all measured samples. For a more appropriate determination of the lifetimes of these LLPs we also used longer pulses (30 and 60 days), and we reported the position of the each specific protein with respect of the entire proteome as “unlabeled percentile rank” which is positively correlated with their stability. For the analysis of the peptides containing two lysines (mis-cleavage analysis; Supplementary Fig. 8), we designed a set of specific experiments, because we identified very few peptides containing two lysines in our original dataset (~15), indicating that our 16 h digestion protocol is particularly efficient. This would be regarded per se as a positive aspect in MS measurements, but it is not useful for this kind of analysis. For this, first, we optimized a protocol that would allow us to detect peptides with two lysines in the different labeling forms. We in-solution digested the brain homogenate of our 21-day pulsed mice using three different proteases, i.e., Chymotrypsin, GluC, ArgC, Trypsin (ProMega) for short-time periods. Briefly, 20 μg of homogenate was denatured using 10 μl of 1% RapiGest (Waters) at 95 °C for 5 min. The disulfide bonds were reduced using 10 mM DTT followed by alkylation using 55 mM iodoacetamide for 30 min each at 37 °C, 750 rpm. The proteins were digested using 1:100 enzyme-to-protein ratio using the 4 proteinases all separately for 2 and 4 h. The digestion reaction was quenched by acidifying using 20 μl of 10% TFA. The undigested proteins were pelleted by centrifuging at max speed (10,000 rpm) for 15–20 min in a table-top centrifuge. The supernatant was desalted using StageTips. Among the conditions tested, we obtained the best results from a 2 h incubation with trypsin. In this case we observed the highest number of peptides containing two lysines (~170 peptides). Among these, 34 were reliably measured in three different biological replicates in their zero-, one- and two-13C-lysine form and are reported in Supplementary Fig. 8. For the analysis of the in vivo double pulse data (Supplementary Fig. 9) we used only the unique peptides that were reliably detected in all four forms (light, Lys6, Arg10 and Lys6-Arg10) with this pulsing scheme (Supplementary Fig. 9a). The percentages reported in Supplementary Fig. 9c-h are related to the total obtained from the sum of all intensities (light, Lys6, Arg10 and Lys6-Arg10). Since we have not measured the pool of arginine, we estimated the lysine efficiency from optimizing only its associated parameter b (1 / tau_sol_) for the arginine model while constraining all other parameters to those found in the lysine-only model. Due to the metabolism of arginine to proline, we analyzed only peptides that contained one lysine, one arginine and no proline. If more than one unique peptide was detected for one protein, the values were averaged for the same protein.

The targeted MS experiments for the detection of β-Galactosidase were analyzed using Skyline (version 3.1).

### Quantification of proteins

To obtain iBAQ values of proteins, 10 μg of brain cortex homogenate and 10 μg of the UPS2 (Universal Protein Dynamic Range Standard mix containing purified 48 human proteins; Sigma) were processed for in-solution digestion using Trypsin and analyzed by LC-MS/MS on an Orbitrap Fusion Tribrid mass spectrometer. The brain cortex homogenate sample and UPS2 were measured seven times, with a total amount of 1 µg of protein on the column. The RAW files were analyzed using MaxQuant software^49^ version 1.5.2.8 based on the Andromeda search engine^50^. The mouse UniProt database (downloaded on 2015.11; containing 16,727 reviewed entries) and UPS2 database (downloaded from Sigma website) was used for identifying and quantifying the proteins. The MaxQuant iBAQ option was ticked for analysis. Further, the obtained iBAQ values^16^ were plotted against the known amounts of the proteins in the UPS2 standard. The calibration slopes were calculated as described^23^ by taking the mean values of the technical replicates. The absolute amounts of proteins present in brain fractions were calculated by linear regression. All contaminants were removed from the protein list and protein abundance was averaged across seven replicates following median normalization and expressed as log_10_ + 10 of the normalized intensities, as it is often done with this typology of data. The quantification of cell culture protein abundances was performed by LFQ as described^51^.

### Amino acid pool modeling and lifetime determination

Lysine in animals is present in at least two pools: the soluble (free) lysine pool and the pool immobilized into proteins^52^. By feeding the SILAC diet, ^13^C_6_-lysines (heavy lysines, *H*_sol_) are incorporated into the free lysine pool (Supplementary Fig. 3c), mixing with the unlabeled lysines (light lysines, *L*_sol_). Since the soluble lysine pool is used for protein synthesis, the heavy lysines from this pool enter into the pool immobilized within proteins (*H*_prot_). At the same time, protein degradation mobilizes lysines from the previously existing proteins (*L*_prot_, and later also *H*_prot_) into the soluble lysine pool. Eventually, lysines are eliminated by excretion. During the feeding with ^13^C_6_-lysines, these four populations of lysines (*L*_sol_*, H*_sol_*, L*_prot_*, H*_prot_) change with time (*t*). Since the mice used in this study are adult, we consider that the overall amount of proteins in adult animals is unchanged over time. The system can then be described with the following set of coupled differential equations:

1 $$\frac{{{\mathrm d}L_{\mathrm {prot}}}}{{{\mathrm d}t}} = - a \times L_{\mathrm {prot}} + a \times L_{\mathrm {sol}}$$

2 $$\frac{{{\mathrm d}H_{\mathrm {prot}}}}{{{\mathrm d}t}} = - a \times H_{\mathrm {prot}} + a \times H_{\mathrm {sol}}$$

3 $$\frac{{{\mathrm d}L_{\mathrm {sol}}}}{{{\mathrm d}t}} = - r \times a \times L_{\mathrm {sol}} + r \times a \times L_{\mathrm {prot}} - b \times L_{\mathrm {sol}}$$

4 $$\frac{{{\mathrm {d}}H_{{\mathrm {sol}}}}}{{{\mathrm {d}}t}} = - r \times a \times H_{{\mathrm {sol}}} + r \times a \times H_{{\mathrm {prot}}} - b \times H_{{\mathrm {sol}}} + c$$

where $a = 1/\tau _{{\mathrm {degr}}}$, $b = 1/\tau _{{\mathrm {sol}}}$, and *c* are the respective rates for protein degradation, lysine excretion, and heavy lysine feeding. Considering that the overall amount of soluble lysines is conserved, the lysines that are absorbed also need to be excreted. The fourth equation can therefore be further simplified by using:

5 $$c = b \times H_{{\mathrm {sol}}} + b \times L_{{\mathrm {sol}}}$$

The parameter *r* accounts for the sizes of the two pools, namely:

6 $$r = \frac{{N \times S_{{\mathrm {prot}}}}}{{S_{{\mathrm {sol}}}}}$$

*S*_prot_ indicates the overall number of proteins, *S*_sol_ the overall number of freely available lysines, and *N* the average number of lysines incorporated in one protein. Thus *r* is the ratio of the number of lysines that are incorporated in proteins and the number of freely available lysines.

*a*, *b*, and *r* are the only independent equation parameters. Solving the coupled set of differential equations using the initial conditions $H_{{\mathrm {sol}}}\left( 0 \right) = 0$, $H_{{\mathrm {prot}}}\left( 0 \right) = 0$, $L_{{\mathrm {sol}}}\left( 0 \right) = 1$, and $L_{{\mathrm {prot}}}\left( 0 \right) = 1$, we find an analytical expression for $H_{{\mathrm {sol}}}\left( t \right)$, $H_{{\mathrm {prot}}}\left( t \right)$, $L_{{\mathrm {sol}}}\left( t \right)$, and $L_{{\mathrm {prot}}}\left( t \right)$. The required measure for understanding the turnover of individual proteins is $H_{{\mathrm {sol}}}\left( t \right)$. The solution for $H_{{\mathrm {sol}}}\left( t \right)$ reflects a double exponential convergence:

7 $$H_{{\mathrm {sol}}} = 1 - A \times e^{ - t/\tau _1} - \left( {1 - A} \right) \times e^{ - t/\tau _2}$$

with *τ*_1_, *τ*_2_ (time constants) and *A* (amplitude) being connected to the equation parameters *a*, *b*, and *r* in the following way:

8 $$\tau _1 = \frac{2}{{a + b + a \times r + C}}$$

9 $$\tau _2 = \frac{2}{{a + b + a \times r - C}}$$

10 $$A = - \frac{{a - b + a \times r - C}}{{2 \times C}}$$

11 $$C = \sqrt{{-4 \times a \times b + (a + b + a \times r)^2}}$$

Note that a double exponential “biphasic” pool dynamics has previously been observed^5^, however without a formal mathematical treatment.

After modeling the pool of soluble lysines, we have taken into consideration the individual proteins of interest (POI). In the following we again assume that the overall amount of POI is conserved over time. For simplicity we have not taken into consideration the minor contribution of the lysines released from a single POI into the soluble pool. The dynamics of an individual POI are then described by the following differential equation:

12 $$\frac{{{\mathrm {d}}H_{{\mathrm {poi}}}}}{{{\mathrm {d}}t}} = - \frac{1}{{\tau _{\mathrm{poi}}}} \times H_{{\mathrm {poi}}} + \frac{1}{{\tau _{{\mathrm {poi}}}}} \times H_{{\mathrm {sol}}}$$

where $1{\mathrm{/}}\tau _{{\mathrm {poi}}}$ is the rate at which the POI is degraded. Using the above mentioned double exponential convergence of the heavy lysine population $H_{{\mathrm {sol}}}$ with amplitudes *A* and 1 − *A*, time constants *τ*_1_ and *τ*_2_ as well as the initial condition $H_{{\mathrm {poi}}}\left( 0 \right) = 0$ we find an analytical expression for$H_{{\mathrm {poi}}}\left( t \right)$:

13 $${H_{{\mathrm {poi}}}\left( t \right) = 1 - e^{ - t/\tau _{{\mathrm {poi}}}} \times \left( {1 - \frac{{A \times \tau _1\left( {1 - e^{t/\tau _{{\mathrm {poi}}} - t/\tau _1}} \right)}}{{\tau _1 - \tau _{{\mathrm {poi}}}}} - \frac{{\left( {1 - A} \right) \times \tau _2\left( {1 - e^{t/\tau _{{\mathrm {poi}}} - t/\tau _2}} \right)}}{{\tau _2 - \tau _{{\mathrm {poi}}}}}} \right)}$$

This result is in agreement with the work of Guan and collaborators^13^.

For the chase experiments, SILAC diet is replaced again with food containing light lysines. The differential equations need to be adjusted accordingly for the terms describing lysine uptake and excretion (terms marked with stars refer to the chase model):

14 $$\frac{{{\mathrm {d}}L_{{\mathrm {sol}}}^ \ast }}{{{\mathrm {d}}t}} = - r \times a \times L_{{\mathrm {sol}}}^ \ast + r \times a \times L_{{\mathrm {prot}}}^ \ast + b \times H_{{\mathrm {sol}}}^ \ast$$

15 $$\frac{{{\mathrm {d}}H_{{\mathrm {sol}}}^ \ast }}{{{\mathrm {d}}t}} = - r \times a \times H_{{\mathrm {sol}}}^ \ast + r \times a \times H_{{\mathrm {prot}}}^ \ast - b \times H_{{\mathrm {sol}}}^ \ast$$

The initial conditions are those of the pulse phase model at the end of the pulse: $H_{{\mathrm {sol}}}^ \ast \left( 0 \right) = H_{{\mathrm {sol}}}\left( {t_{{\mathrm {end}}}} \right)$, $H_{{\mathrm {prot}}}^ \ast \left( 0 \right) = H_{{\mathrm {prot}}}\left( {t_{{\mathrm {end}}}} \right)$, $L_{{\mathrm {sol}}}^ \ast \left( 0 \right) = L_{{\mathrm {sol}}}\left( {t_{{\mathrm {end}}}} \right)$, and $L_{{\mathrm {prot}}}^ \ast \left( 0 \right) = L_{{\mathrm {prot}}}\left( {t_{{\mathrm {end}}}} \right)$. The analytical expression for $H_{{\mathrm {sol}}}^ \ast \left( t \right)$ for the time after the end of the pulse is then again a double-exponential convergence:

16 $$H_{{\mathrm {sol}}}^ \ast \left( t \right) = A_1 \times e^{ - t/\tau _1} + A_2 \times e^{ - t/\tau _2}$$

Using amplitudes:

17 $$A_1 = \frac{{\left( {C - a + b + a \times r} \right) \times H_{{\mathrm {sol}}}\left( {t_{{\mathrm {end}}}} \right) - 2 \times a \times r \times H_{{\mathrm {prot}}}\left( {t_{{\mathrm {end}}}} \right)}}{{2 \times C}}$$

18 $$A_2 = \frac{{\left( {C + a - b - a \times r} \right) \times H_{{\mathrm {sol}}}\left( {t_{{\mathrm {end}}}} \right) + 2 \times a \times r \times H_{{\mathrm {prot}}}\left( {t_{{\mathrm {end}}}} \right)}}{{2 \times C}}$$

For the solution of $H_{{\mathrm {poi}}}^ \ast \left( t \right)$, with the initial condition being the condition at the end of the pulse, $H_{{\mathrm {poi}}}^ \ast \left( 0 \right) = H_{{\mathrm {poi}}}\left( {t_{{\mathrm {end}}}} \right)$, the analytical expression is:

20 $$ {H_{{\mathrm {poi}}}^ \ast \left( t \right) = e^{ - t/\tau _{{\mathrm {poi}}}} \times \left( {H_{{\mathrm {poi}}}\left( {t_{{\mathrm {end}}}} \right) - \frac{{A_1 \times \tau _1 \times \left( {1 - e^{t/\tau _{{\mathrm {poi}}} - t/\tau _1}} \right)}}{{\tau _1 - \tau _{{\mathrm {poi}}}}} - \frac{{A_2 \times \tau _2 \times \left( {1 - e^{t/\tau _{{\mathrm {poi}}} - t/\tau _2}} \right)}}{{\tau _2 - \tau _{{\mathrm {poi}}}}}} \right)}$$

In order to determine the lifetimes (*t*_1/2_) of all proteins one can fit the experimental data for every single POI with the function for $H_{{\mathrm {poi}}}\left( t \right)$ as given above. However, since all proteins are built from the same amino acid pool, we treat the equation parameters *a*, *b*, and *r* as global, i.e., common for all proteins, and extract only the protein half-lives from the individual fits, thereby obtaining much more robust fit results. We performed an optimization of the equation parameters in order to obtain the values {*a*, *b*, *r*} of the soluble pool $H_{{\mathrm {sol}}}\left( t \right)$ which best describes our experimental data. Therefore, we fitted the labeling dynamics of all POIs that were reliably detected at all times in the brain cortex homogenate (2409) with different global equation parameter combinations. To account for possible effects due to the degradation of the entire proteome, we included in this fitting also the data obtained from the pulse-chase experiments as schematized in Supplementary Fig. 3e–f. We compared the sum of square deviations of the measured data points from the fitted curves for the complete dataset. The minimum of this value, indicating the best equation parameter combination, was used to fit the experimental data of all POIs using the function $H_{{\mathrm {poi}}}\left( t \right)$ (Supplementary Fig. 3 and see Supplementary Fig. 5 for some fitting examples). In addition, we also verified that the soluble pool $H_{{\mathrm {sol}}}\left( t \right)$, obtained from equation parameter optimization, agrees well with all other experimental measures that we observe from other five completely different sets of experiments (the GC-MS measurements of the plasma in Supplementary Fig. 4b, the transgenic approach in Supplementary Fig. 4f, the mis-cleavages analysis in Supplementary Fig. 8, the in vivo double pulse approach in Supplementary Fig. 9 and the pulse and chase approach in Supplementary Fig. 3i).

### RNA extraction sequencing and quantification

Total RNA was extracted from flash-frozen tissue homogenate using the QIAzol Lysis reagent (Qiagen GmbH) processed with RNeasy spin columns and DNase to purify RNA and remove residual contaminating genomic DNA. Library preparation for mRNA sequencing was performed according to Illumina standard protocols using the TruSeq RNA Sample Prep Kit v2. Libraries were quality controlled and quantified using a NanoDrop 2000 (ThermoFisher Scientific), an Agilent 2100 Bioanalyzer (Agilent Technologies) and Qubit (ThermoFisher Scientific). Single-end 50 bp sequencing data were generated on an Illumina HiSeq2000™ using Illumina TrueSeq SBS kits. The data represented in Fig. 1b are expressed as log_10_ of the mRNA counts expresses as fragments per kilobase million.

### Cell-type-specific sorting of nuclei

Neuronal and glial nuclei sorting were performed as described^53^ with slight modifications. Briefly, flash frozen mice cortices were homogenized, cross-linked (1% formaldehyde) and quenched (0.125 M glycine) in room temperature for both 5 min. The crude nuclei pellets were further purified through sucrose gradient, stained with conjugated anti-NeuN-Alexa488 (MAB377X, 1:1000) for 20 min and re-suspended into nuclei suspension buffer (0.2% tween-20, 1% BSA, 1x Roche complete EDTA-free protease inhibitor cocktail in 1× PBS). As a negative control, anti-mouse-Alexa488 (A-11029, 1:2000) was used. Sorting was done with BD FACSARIA III containing 85 µm nozzle. Gating was done based on nuclei size, aggregate exclusion and Alexa488 fluorescence. Nuclei were sorted into BSA-coated falcon tubes, spun down, flash-frozen and stored at −80 °C until further processing. The effectiveness of the sorting was confirmed by mounting a small fraction of the sorted nuclei and imaging in an inverted Nikon Ti epifluorescence microscope (Nikon Corporation, Chiyoda, Tokyo, Japan) equipped with a ×20 air objective. Following sorting the nuclei were in-gel digested and processed for MS as explained in the Methods.

### Hippocampal cultures and pharmacological treatments

First coverslips were prepared by cleaning them with nitric acid overnight. After acid treatment, coverslips were washed thoroughly with double distilled water, were sterilized and were coated overnight with 1 mg/ml PLL. After coating, coverslips were washed thoroughly with sterile water, and were incubated with plating medium (MEM supplemented with 10% horse serum, 3.3 mM glucose, and 2 mM glutamine). Neuronal hippocampal cultures were obtained from dissociated hippocampi of E18 mice^54^. In brief, brains were extracted from the skulls of E18 mice, and the hippocampi were isolated under a dissection microscope. Following three washes with HBSS (Invitrogen, Waltham, MA, USA) to remove tissue debris, the hippocampi were incubated for 15 min in enzyme solution, as described^54^. Following dissection, neurons were plated at a concentration of ~30,000/cm^2^ and were left to adhere for 1–4 h at 37 °C in a 5% CO_2_ cell-incubator. After adhesion, the medium was changed to Neurobasal-A medium (Gibco, Life Technologies, Carlsbad, CA, USA) containing 1:50 B27 supplement (Gibco) and 1:100 GlutaMAX (Gibco). Neurons were kept in culture at 37 °C and 5% CO_2_ for 15 days before pharmacological treatments. Cultures were treated for 24 h with either Lactacystin (Santa Cruz; CAS 133343–34–7) at the final concentration of 10 µM^42^ or Cycloheximide (SIGMA; C4859) at a final concentration of 75 µM. No evident signs of cellular stress were observed after the treatments. Samples were extracted in 8 M Urea and precipitated using 4 volumes of acetone. Resuspended lysate after protein estimation was trypsinized in-solution overnight, and loaded on Q-Exactive Hybrid Quadrupole Orbitrap mass spectrometer. Protein abundance was determined by LFQ as described^51^, and the relative difference in protein abundance was defined with respect to an untreated control. Three biological replicates corresponding to three independent neuronal cultures were used for these experiments. The protein abundance change following treatment vs. the control is plotted in Supplementary Fig. 23 against the protein lifetimes that were measured in vivo in the brain homogenate sample.

### Determination of protein surfaces

Protein surface estimates (Supplementary Fig. 23e) were derived from information on protein structures obtained from the protein database (PDB). The following proteins have been analyzed: actin, AMPAR, amphiphysin, AP1 beta, AP1 mu, AP180, bassoon, CAMKII kinase, citrate aconitase, citrate synthase, clathrin Heavy Chain, clathrin Light Chain, ClC3, complex 1, complex 2 and complex 3 of the mitochondrial respiration chain, creatine kinase U type, CSP, dynamin, endophilin, epsin1, fATPase alpha, beta, delta, epsilon and gamma, GABABR1, gephyrin, hexokinase, homer, intersectin1, l1cam, mGluR1, mGluR5, monoamine oxidase a, munc18, neurexin, neuroligin, NFL, NMDAR, nrcam, piccolo, PIPKIgamma, psd95, rab3a, rab3b, rab3c, RIM1, SCAMP1, shank2, SNAP25, SNAP29, SV2a, SV2b, SV2c, synapsin1, synapsin2, synapsin3, synaptobrevin2, synaptophysin1, synaptotagmin1, syndapin, syntaxin1a, tubulin, VAMP4, vGlut1, vGlut2, vti1b, the vATPase a, c, and d subunits, and the soluble vATPase complex (subunits A-H). Most structures have been previously described in our publications^22,23^. For the proteins that were not in our previous publications, complete structures were created by combining the structural data from the protein data bank (www.rcsb.org) to additional information based on similar proteins. In detail the following PDB IDs were used: AMPAR, 3KG2; neuroligin 3BIX and 3BIW; neurexin 3BIW and 2H0B; l1cam, 1CFB; tubulin, 1JFF. The triangle mesh based solvent accessible surface (SAS) was calculated subtracting the meshes, which were shielded by other proteins or lipid membranes, resulting in the best polygon surface approximation for the exposed surface.

### Bioinformatics, date representation, data analysis, and statistics

Protein information such as protein length was retrieved from The Universal Protein Resource (UniProt Consortium^55^). The isoelectric point was calculated with ExPASy (https://web.expasy.org/compute_pi/) and the grand average of hydropathy (GRAVY) was calculated with the online sequence manipulation tool (http://www.bioinformatics.org/sms2/protein_gravy.html). For disorder prediction, IUPred^56^ was used with a cutoff of 0.5 for disorder definition in mode “short or long” with canonical protein sequences acquired from Uniprot. Percentage of the sequence that is above this threshold is reported for every protein. Due to the incongruence of protein classification and localization information for brain proteins, for protein categorization we relied on a database that we built by manual integration of three other databases (Synprot^19^, SynaptomeDB^20^ and G2C:Genes to Cognition^21^), alongside with previous publications^22,23^ obtaining the reliable localization/affiliation of ~1200 proteins as detailed in Supplementary Data 1. We have used this categorization for the classification presented in Figs. 2, 3 and Supplementary Fig. 20. For the protein family and the molecular complex affiliation we relied on previously published data^55^. All analyses were performed with the help of Matlab (The Mathworks Inc., Natick, MA, USA), GraphPad Prism (GraphPad Software) or SigmaPlot software (Systat Software), using self-written routines. For the fuzzy c-means clustering we used the appropriate function of the Fuzzy Logic Toolbox. Lifetimes were determined as explained above, in the section “Amino acid pool modeling and mathematical lifetime determination”. Functional enrichment analysis was performed with the WEB-based GEne SeT AnaLysis Toolkit^57^ or with STRING^36^, a functional protein association tool to analyze and visualize protein networks. For the functional enrichment analysis, we relied on the overrepresentation enrichment analysis (ORA) and on the non-redundant gene ontology modules, to avoid synonym terms. The *P*-values for this analysis were adjusted with the Holm-Bonferroni method and the false discovery rates were reported as –log_10_ (where higher values indicate more relevant significance). The 3D representation of the synapse and of the synaptic vesicle with the appropriate number of molecules was adapted from our previous work^23^. For the sorted nuclei, to avoid any possible detection bias, protein lifetimes were calculated only for proteins that were found both in neurons and in glial cells where the difference in intensity between the two cell types was ≤500%. The cellular expression specificity for microglia, neurons, astrocytes, and oligodendrocytes was defined from the data published by Sharma and collaborators as were >10-fold more abundant in one cell type compared with all the others cell types^37^ (following the principle proposed by the authors). The data for the GABAergic and glutamatergic neurons was obtained from a previous work^38^. The results on the reduction of protein degradation were obtained from the work of Hakim and collaborators^42^, and expressed as in the original work as log_2_H/M. Venn diagrams were initially plotted with Venny (http://bioinfogp.cnb.csic.es/tools/venny/). The calculation of the error in the determination of the lifetimes is summarized for the brain datasets in Supplementary Fig. 6–7. The average *r*^2^ of the fittings and the average 95% confidence interval in Supplementary Fig. 6b, c are reported for all the datasets and they are largely overlapping. The surfaces of the proteins were determined as indicated in the previous section. The exposed surfaces for soluble proteins were considered to be equal to their actual surfaces. For transmembrane or membrane-attached proteins, we considered the membrane-facing sides as non-exposed, and reduced the exposed surfaces accordingly. The same was performed for proteins that were members of protein complexes, since surfaces covered by partner proteins were not considered to be exposed. A further reduction was performed for proteins that are found in polymers of complexes, such as tubulin. For protein lifetimes we relied on the 95% confidence interval, since it is the most appropriate measure of error for the typology of data that we are reporting^58^. All *P*-values for multiple comparisons were adjusted with the Bonferroni correction and they were calculated from the confidence intervals as reported elsewhere^59^.

## Electronic supplementary material

Supplementary Information

Description of Additional Supplementary Files

Supplementary Data 1

## Data Availability

The proteomic datasets are accessible with the PRIDE ID PXD010859
