# Supplementary material for: Precisely measured protein lifetimes in the mouse brain reveal differences across tissues and subcellular fractions
Source: Nat Commun. 2018 Oct 12;9:4230. doi: 10.1038/s41467-018-06519-0 (PMC6185916; doi:10.1038/s41467-018-06519-0)
Supplement: Supplementary file 1 — Supplementary Information [file 41467_2018_6519_MOESM1_ESM.pdf]

Supplementary Information
of the manuscript:

Precisely measured protein lifetimes in the mouse brain reveal differences across tissues and subcellular fractions

Fornasiero et al., 2018

Supplementary Figures:

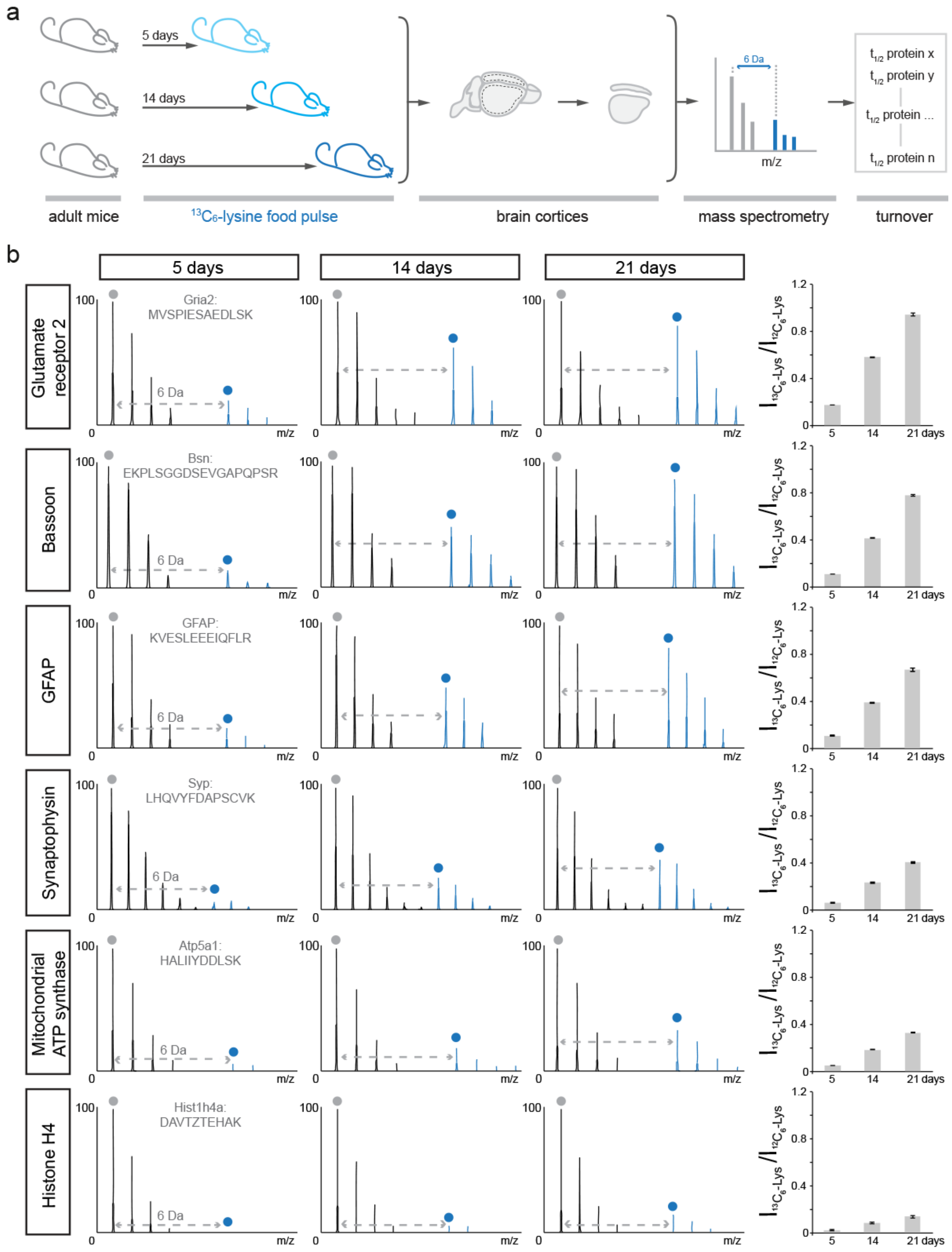

Supplementary Fig. 1 | General procedure of the metabolic pulsing of mice. a, Mice were pulsed with a SILAC diet, containing $^{13}C_6$-lysine, for different lengths of time (5, 14 or 21 days), followed by cortex dissection and mass spectrometry analyses, to reveal relative incorporation rates. These, in turn, are interpreted to provide protein lifetimes (see also **Supplementary Fig. 3 and 5**). **b**, Exemplary MS1 scan of 6 peptides. The blue peaks indicate the $^{13}C_6$-lysine-containing peptides while the ones shown in black represent the regular unlabeled peptide. The ratio between the $^{13}C_6$-lysine-containing peptides and the regular ones is shown in the bar graphs on the right. Data are presented as mean ± s.e.m.; n = 3. Relative incorporation increases steadily during the pulsing, at different rates for the different proteins, indicating that they turn over with different speeds.

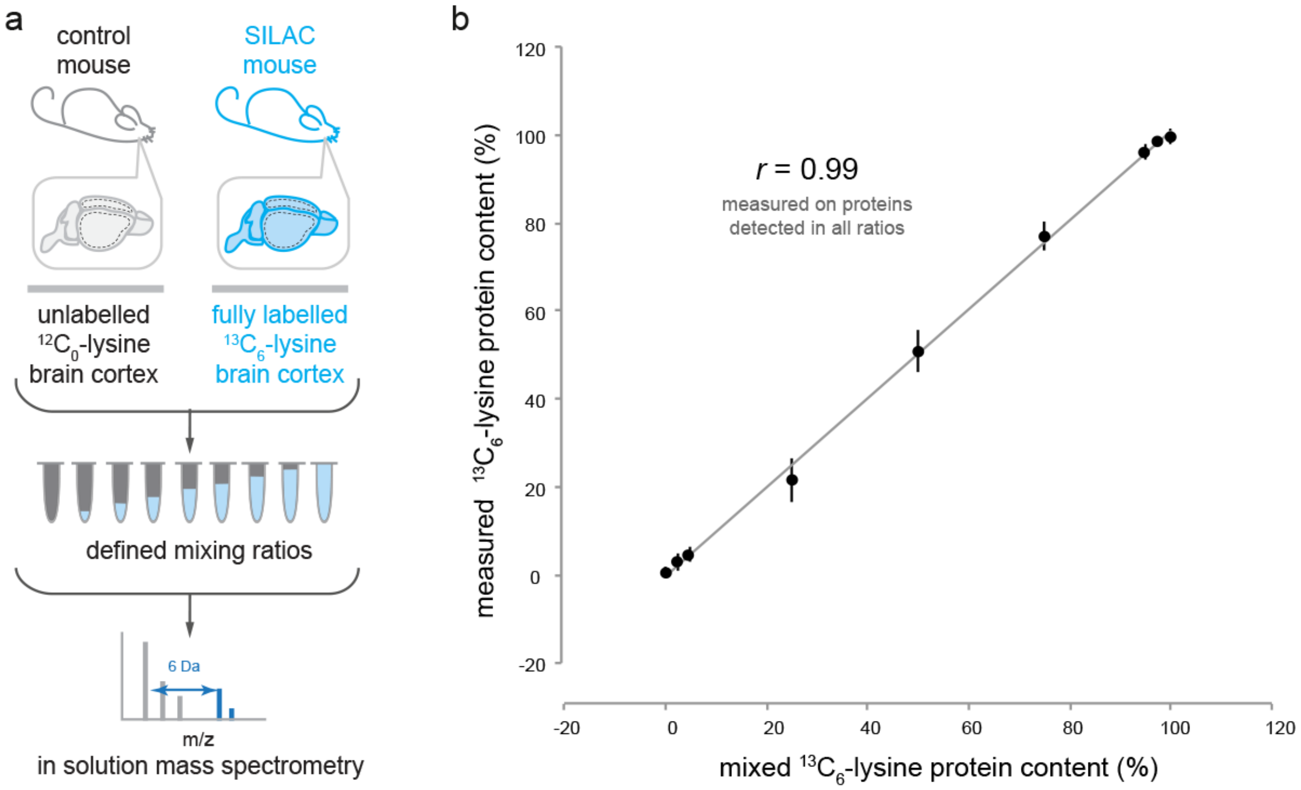

Supplementary Fig. 2 | $^{13}C_6$-lysine-containing peptides are detected accurately. a, To test whether the $^{13}C_6$-lysine-containing peptides were detected as accurately as the normal ones, we prepared a mock experiment. We mixed cortices from a $^{12}C_6$-lysine control mouse and from a full SILAC mouse, in different ratios, and measured the resulting mixture by mass spectrometry. **b**, The measured contents of $^{13}C_6$-lysine-peptides was virtually identical to the one expected from the mixture ratios ($r = 0.99$, P-val < 0.001).

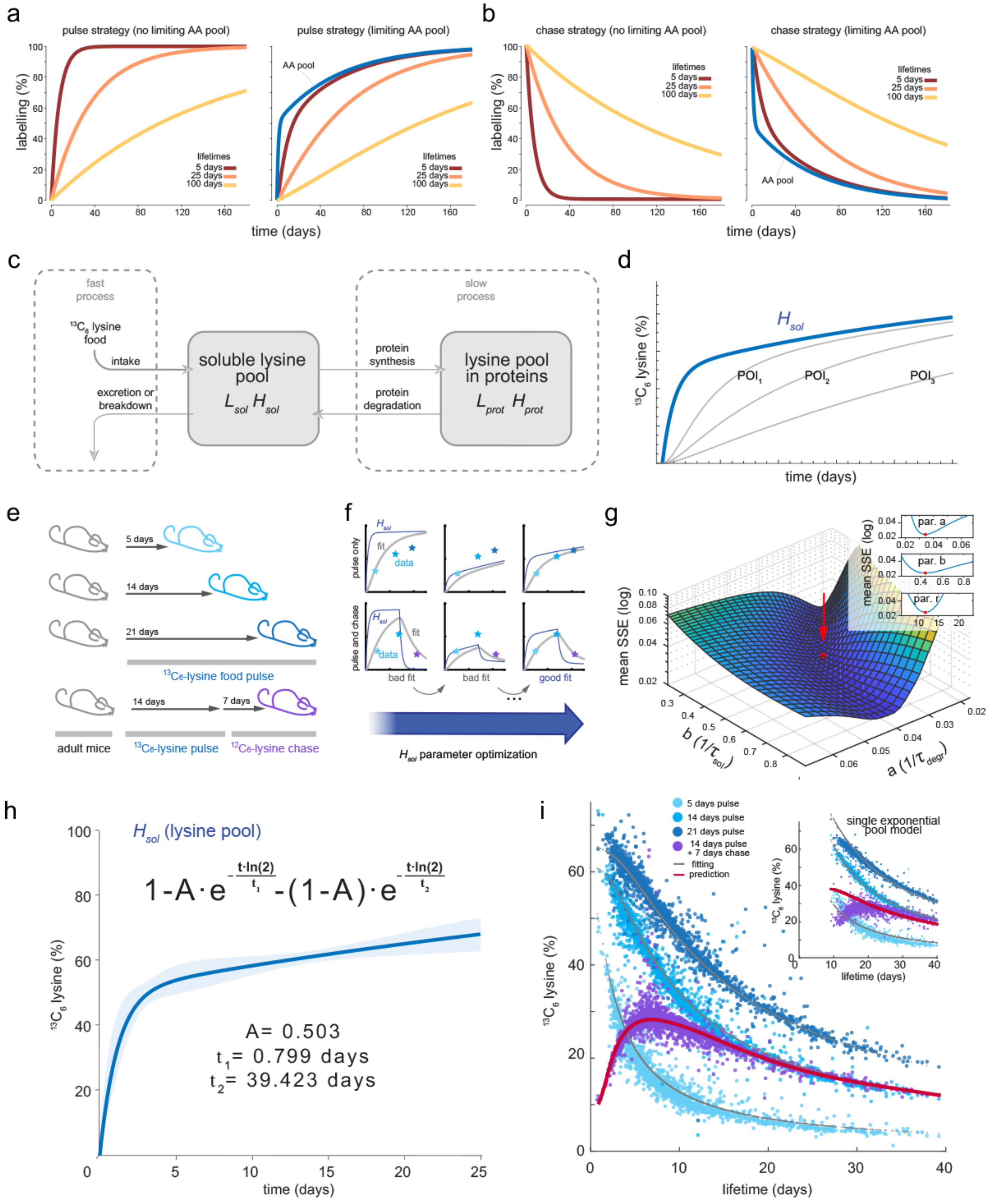

Supplementary Fig. 3 | Model for the availability of lysine during protein turnover. a, Pulsing strategy for the study of protein turnover *in vitro* (left) and *in vivo* (right). *In vitro* essential amino acids can be substituted by a rapid medium change, and the $t_{1/2}$ of proteins (referred to in this work as lifetime) can be measured directly from the labeling of different proteins (see examples in the left plot). *In vivo* the situation is more complicated. Animals absorb essential amino acids from the diet, just as the cells do, but the way the amino acid levels change in the body depends on the metabolism of the entire proteome, since amino acids can be recycled following protein degradation, and re-enter the amino acid pool available for protein neo-synthesis. As a consequence, in order to determine protein lifetimes *in vivo* it is necessary to study the behavior of the amino acid pool. **b**, Same as in **a**, but for a chase strategy. **c**, Lysine is present in at least two

Supplementary Fig. 3 legend continuing from the previous page:
pools in the mammal organism: the soluble (free) lysine pool, which is in equilibrium with food intake, and the pool immobilized into proteins. Initially all lysines are light ($^{12}C_6$) in the mice fed with normal food. SILAC food intake delivers $^{13}C_6$-lysines (heavy lysines, *H*) into the soluble lysine pool (H_{sol}), which is used for protein synthesis, thereby placing the heavy lysines into proteins (H_{prot}). At the same time, protein degradation delivers light lysines (*L*) from the protein pool (L_{prot}) to the soluble pool (L_{sol}). Eventually, lysines are eliminated by excretion from the soluble pool. We solved the model represented here (see methods for details). **d**, Graphical representation of the free lysine pool. For simplicity only H_{sol} is plotted (since its temporal behavior is sufficient to determine the protein lifetimes). H_{sol} is influenced by the fast rise of $^{13}C_6$-lysines from the food intake and by the slower exchange with the protein pool, resulting in a double-exponential behavior. Assuming a constant rate of production and degradation, each protein is likely to be turned over in a single exponential process. It is important to point out that the availability of $^{13}C_6$-lysines in the soluble pool determines the apparent labeling of proteins. This renders the interpretation of the turnover of the proteins more complex, and therefore their lifetimes cannot be derived from single exponential fits. Instead we have to fit a function that takes into account the double exponential behavior of the soluble lysine pool (see online Methods). This function is shown here for three exemplary proteins of interest (POIs) with different turnover rates. **e-f,** Strategy for parameter optimization. We also included a chase step to make sure that our model presented in **c** is correct. While the lifetime of each POI is different, all POIs are synthetized from the same H_{sol}. We optimized for H_{sol} by fitting all POIs using the same double-exponential parameters. Using various parameter combinations and minimizing the sum of square errors (SSE), the most likely soluble pool H_{sol} is eventually found. **g**, Graphical representation of the minimum in the SSE as a function of the parameters of the H_{sol} (see online Methods for details). The red arrow indicates the minimum. The inset above the surface shows slices through the minimum of the SSE for all three parameters of the double-exponential function. **h**, Equation and graphic representation describing H_{sol}, as obtained from the parameter optimization. The light blue area indicates the standard errors of the optimization. Please note that $t_1 = \tau_1 \cdot \ln 2$. **i**, Single lysine incorporation percentages, across different pulse times vs. the calculated lifetimes. Each point represents a single protein from the cortex homogenate dataset. The light gray lines indicate the respective fittings of the points. The pulse-chase data are represented in purple, and the red line indicates the predicted labeling for this dataset. Although the pulse-chase data were only used for the refinement of the lysine pool, their distribution is in agreement with the predicted lifetimes obtained with the pulse only strategy (see also Supplementary Fig. 10 for the analysis of proteins that deviate from the prediction). To test the reliability of our model we used the comparison method, where we compared the two-pool model with a single exponential pool model, as shown in the inset. Here, the H_{sol} pool dynamics are disregarded, and are instead assumed as being 100% and 0% during pulse and chase phases, respectively (as in the left sides of panels **a** and **b**). Individual POI fit results show generally longer lifetimes, but also large fit residuals towards shorter lifetimes. For the global comparison of the two models we used the probability of the selected model (Akaike weight), based on the Akaike information criterion (AIC)[1]. The Akaike weight is a number comprised between 0 and 1, where 0 indicates the least probable model and 1 indicates the most probable model. This measure takes into account the complexity of the model, by adding penalties to the more complex models. The Akaike weight from our two lysine pool is indistinguishable from 1, indicating that this model is far better than a model assuming a single exponential change of lysine availability (which has an Akaike weight indistinguishable from 0).

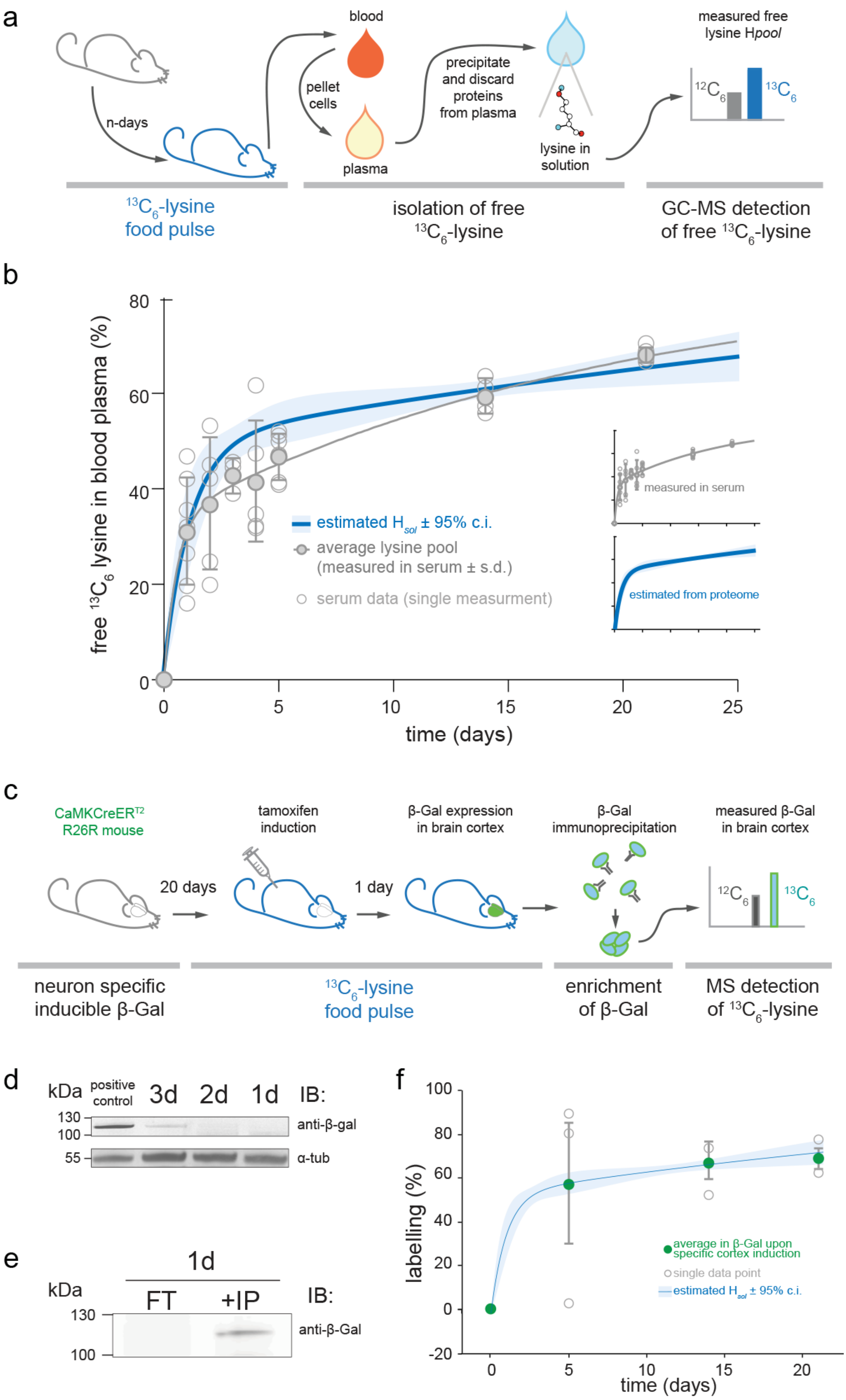

Supplementary Fig. 4 | *In vivo* confirmation of the reliability of the lysine pool model presented in Supplementary Fig. 3. a, A result of the optimization presented in **Supplementary Fig. 3** is that the food-

Supplementary Fig. 4 legend continuing from the previous page:
derived lysine pool is rapidly saturated with $^{13}C_6$-lysine (within ~1 day). To test this notion, we pulsed mice with $^{13}C_6$-lysine food, and isolated the free lysines from the blood plasma following precipitation of the blood proteins (to avoid contamination from lysines immobilized into proteins). In this fraction we measured the content of labeled lysines (H_{sol}) after derivatization using gas-chromatography mass spectrometry (GC-MS). **b**, The kinetics of free $^{13}C_6$-lysine enrichment in the deproteinized plasma (dots) are close to the estimated behavior of free $^{13}C_6$-lysine in our model (line). N = 33 independent experimental measures, corresponding to one animal each. **c**, A second notion deriving from the model is that the lysine pool resulting from the degradation of previously existing proteins is very slowly replaced by $^{13}C_6$-lysine, and provides normal lysines for incorporation into newly synthesized proteins for a long time period. In simple terms, even after our longest pulse (21 days), the mouse proteome would "release" unlabeled lysines, which would mix with the labeled ones and contribute to new protein synthesis. An elegant, although laborious way to test this specifically in the brain is to induce the expression of a protein for a very short time, after the metabolic labeling of the mice. To test this, we measured the $^{13}C_6$-lysine incorporation in a protein expressed on cue, after 4, 13 or 20 days of feeding with the heavy lysine. We generated a CaMKCreER^{T2} R26R reporter mouse line by crossing the R26R LacZ reporter mouse line[2] to the tamoxifen-inducible neurospecific CaMKCreER^{T2} driver line, which is specific for excitatory neurons of the forebrain[3]. We injected these reporter mice with tamoxifen thereby inducing the expression of the exogenous β-galactosidase (β-gal) reporter in neurons for one day. The cortex was then extracted, β-gal was immunoprecipitated and analyzed by mass spectrometry. **d**, Low levels of β-gal are expressed after 1 day of induction, and they become clearly visible after 3 days of induction by Western Blotting (the positive control in the left lane indicates a mouse injected with tamoxifen for 5 days, twice daily, and sacrificed 10 days later, for comparison purposes). **e**, Since we aim at inducing β-gal in mice for a short period (1 day only), we needed to rely on an immunoenrichment strategy to obtain sufficient amount of β-gal that can be analyzed by targeted-MS. The protein is evident in Western Blotting after enrichment. **f**, Measured β-gal labeling overlaid with the labeling predicted by our model. Even after 20 days of SILAC diet feeding, the labeling of β-gal is ~70%, since about 30% of the lysines used in biosynthesis are still normal lysines, coming from the degradation of previously existing proteins. This value was virtually identical to the measured levels of $^{13}C_6$-lysine in β-gal. Data are presented as mean ± s.e.m. N = 9 independent biological measurements, corresponding to one mouse each.

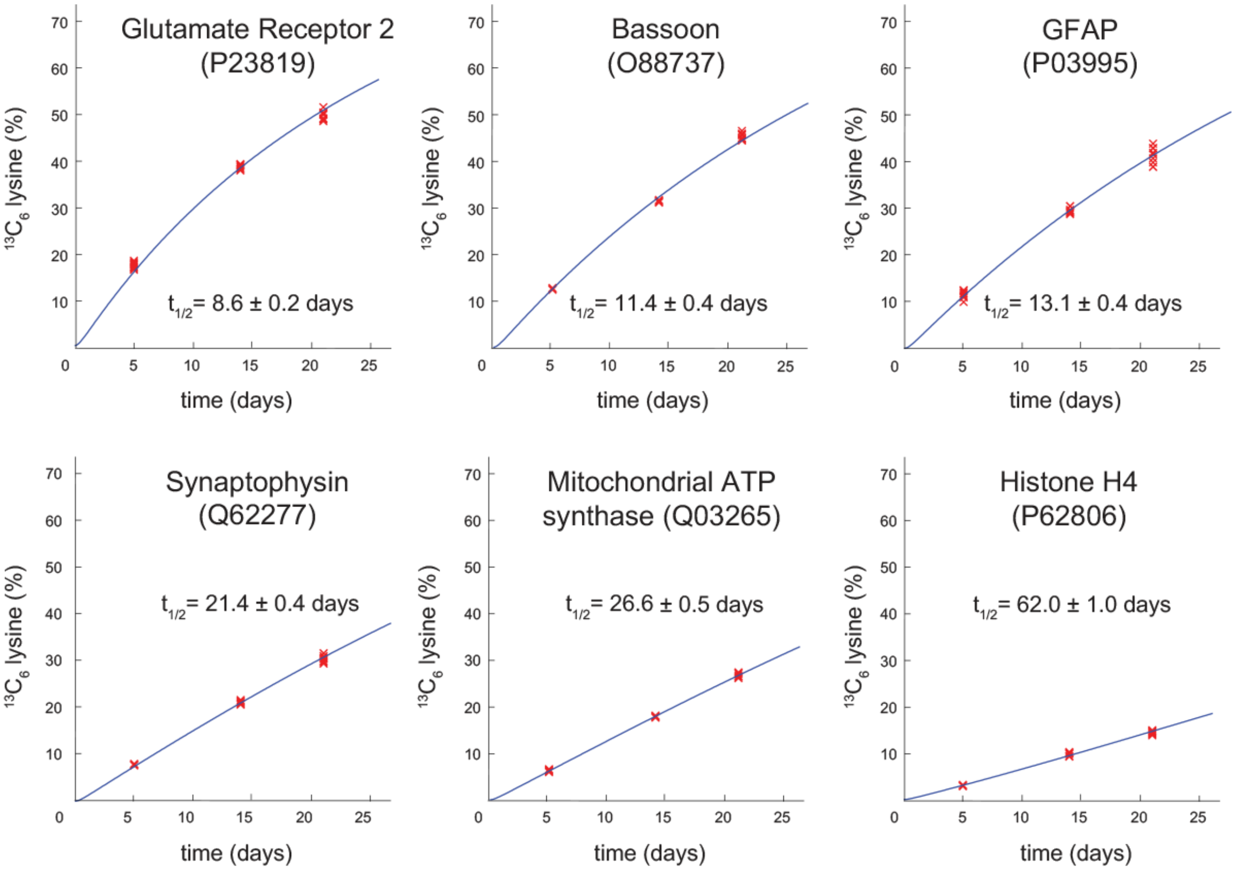

Supplementary Fig. 5 | Determination of the protein lifetimes. Using the optimized parameters (**Supplementary Fig. 3**) and after confirming the reliability of our model *in vivo* (**Supplementary Fig. 4**), we performed precise fits to the $^{13}C_6$-lysine amounts measured from the spectra, as shown here for the exemplary proteins presented in **Supplementary Fig. 1b**. These provide the protein lifetimes (expressed as $t_{1/2}$ of each POI, see also **Supplementary Data 1**).

a

tissue	fraction	treatment	number of measured lifetimes	median r^2 of fitting	median 95% conf. interval (% of lifet.)	median data points for fitting	lifetimes r^2 > 0.7	lifetimes c.i. < 10%
cortex	homogenate	control	2398	0.98	6.19	13	1918	1432
cortex	synaptosome	control	2312	0.88	8.67	10	1541	1188
cortex	syn. ves.	control	2705	0.94	7.36	17	2066	1615
cerebellum	homogenate	control	2693	0.97	9.25	9	1977	1299
cerebellum	synaptosome	control	1926	0.97	8.24	10	1434	975
cerebellum	syn. ves.	control	2413	0.95	8.05	13	1812	1296
cortex	homogenate	environ. enr.	2471	0.96	11.95	8	1657	962
cortex	synaptosome	environ. enr.	1419	0.96	9.12	10	1068	683
cortex	syn. ves.	environ. enr.	2212	0.96	9.54	11	1583	983
all	all	all	3559	0.81	1.45	8	2143	1077

b

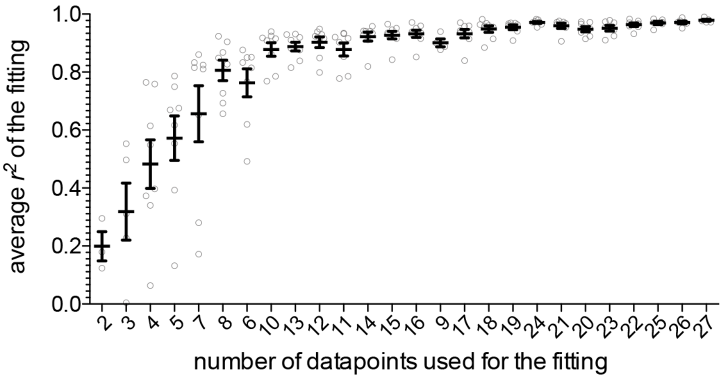

c

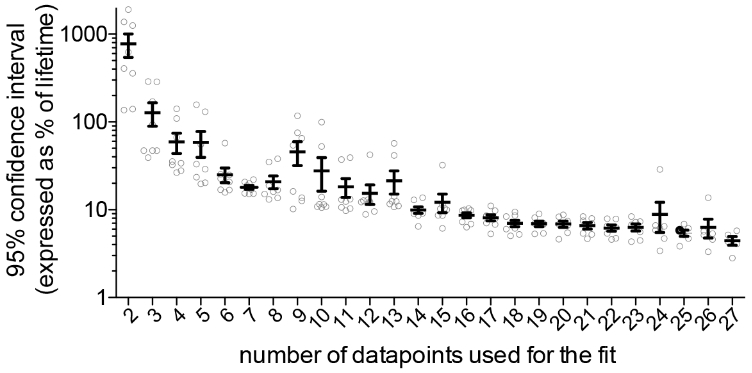

Supplementary Fig. 6 | Systematic quality evaluation of the brain protein lifetime datasets. a, Table resuming size and quality of the protein lifetime datasets presented in this work (for individual r^2 and 95% confidence intervals refer to **Supplementary Data 1**). The minimum r^2 of the fitting that was used for bioinformatic analyses was 0.7. **b**, Dependency of the average r^2 on the number of points used for the fitting. The graph includes the measurements of all datasets (for a more detailed analysis refer to **Supplementary Fig. 7 a-i**). If 10 or more independent datapoints are used in the fitting, the results become inherently reliable. **c**, Same as in **b** but for the 95% confidence interval (c.i.) in the determination of the lifetime (expressed as a % of each measured lifetime; for a more detailed analysis refer to **Supplementary Fig. 7 j-r**). As a small technical note, the 95% c.i. is the most reliable measure of the error for this typology of data. When more than 15 datapoints are used in the fitting, the confidence interval becomes ~10% of the lifetime (meaning that a protein with a lifetime of ~3 days will have a confidence interval of ~0.3 days in the determination of the lifetime).

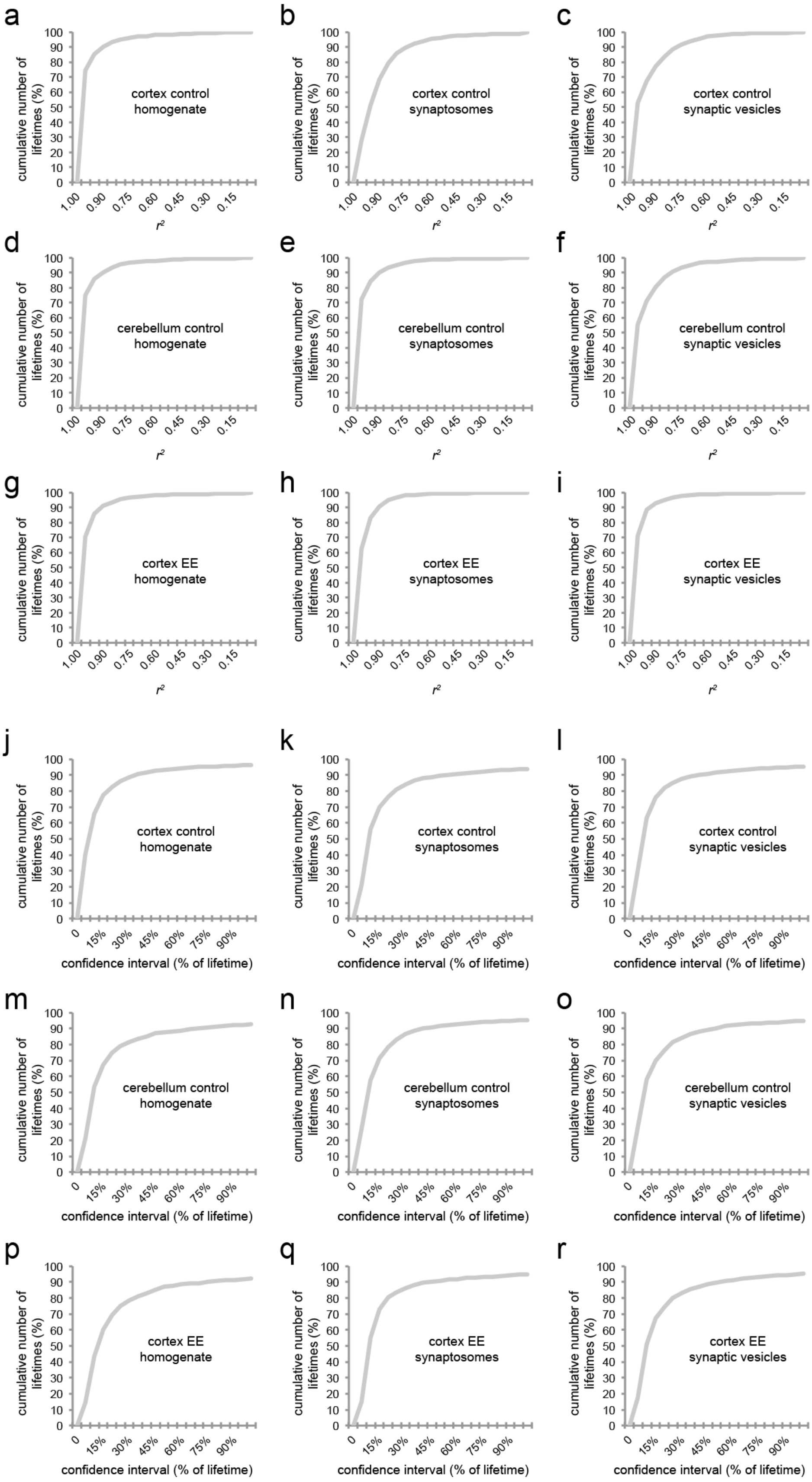

Supplementary Fig. 7 | Detailed quality evaluation of the lifetime datasets. a-i, Distribution of lifetimes *vs.* r^2 expressed as cumulative histograms. The large majority of datasets have lifetime determinations with r^2 > 0.7, indicating reliable curve fitting. **j-r**, Same as in previous panels but for the confidence interval (expressed as a % of each lifetime). The large majority of lifetimes is determined with confidence intervals lower than 30% for each lifetime.

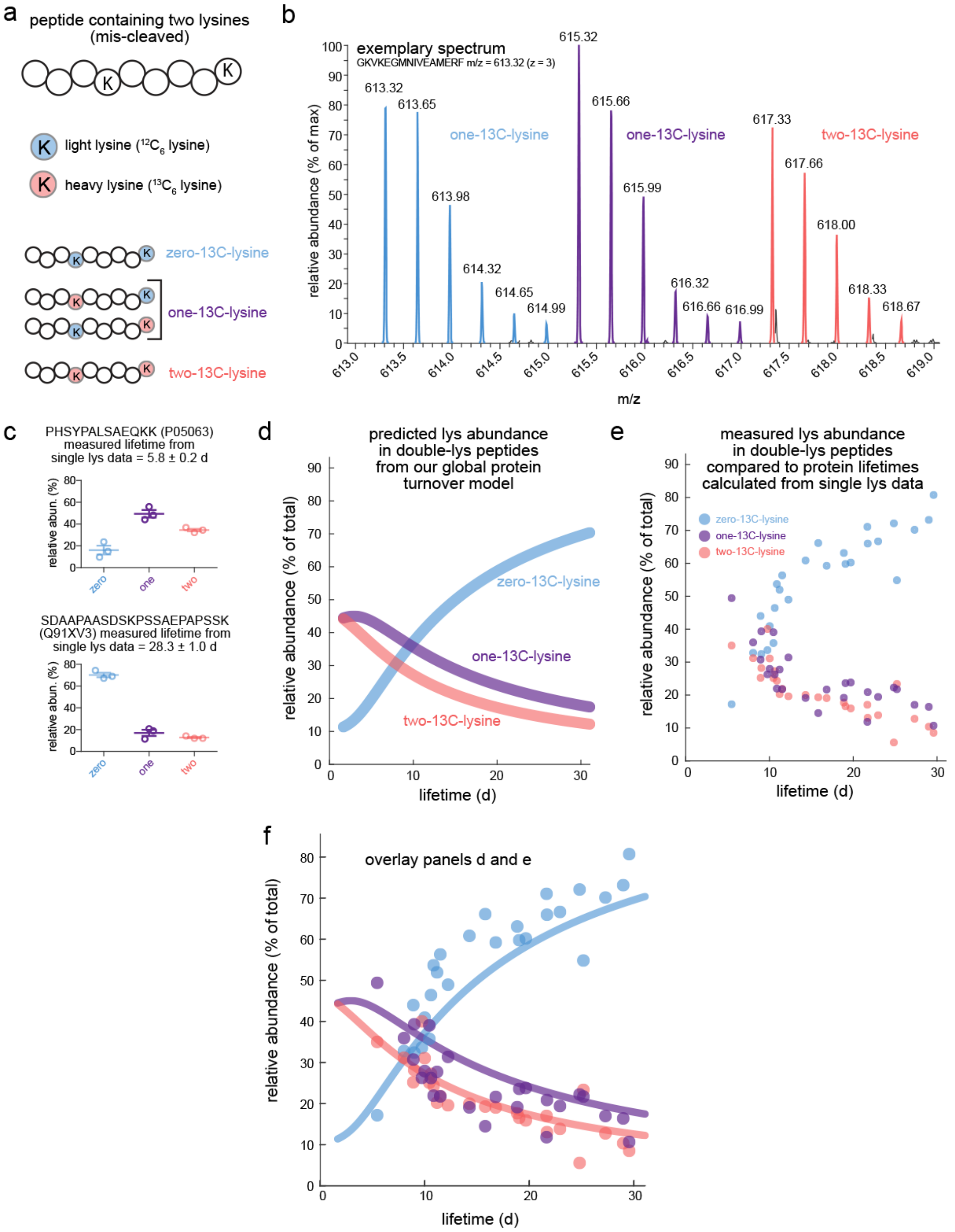

Supplementary Fig. 8 | *In vivo* analysis of the peptides containing two lysines (mis-cleavage analysis) confirms the validity of the lifetime measurements. a, Schematic representation of the possible labeling status of peptides containing either two unlabeled lysines ($^{12}C_6$; zero-13C-lysine), one unlabeled and one labeled lysine ($^{12}C_6$ and $^{13}C_6$; one-13C-lysine) or two labeled lysines ($^{13}C_6$; two-13C-lysine). **b**, Exemplary spectrum of one peptide from mice pulsed for 21 days showing the three possible labeling forms. **c**, Relative abundance of the zero-, one- and two-13C-lysine form of two peptides from mice labeled for 21 days (N = 3 independent biological replicates). **d**, Predicted distribution of the three labeling profiles at 21 days *versus* the determined protein lifetimes based on the lysine pool availability (from **Supplementary Fig. 3-4**). **e**, Measured labeling profiles of double-lysine peptides from mice pulsed for 21 days *versus* the lifetimes that were determined as in the strategy explained in **Fig. 1** and **Supplementary Fig. 1, 3** and **5**. **f**, Overlay of the predicted and the measured lysine abundance in measured peptides. While initially the differences in labeling seemed difficult to interpret (see panel **c**), we realized that the labeling depended on the lifetimes of the respective proteins, and therefore could be easily explained if these were taken into account. The miscleavage analysis is therefore an additional confirmation of the validity of the model we used for the determination of the lifetimes.

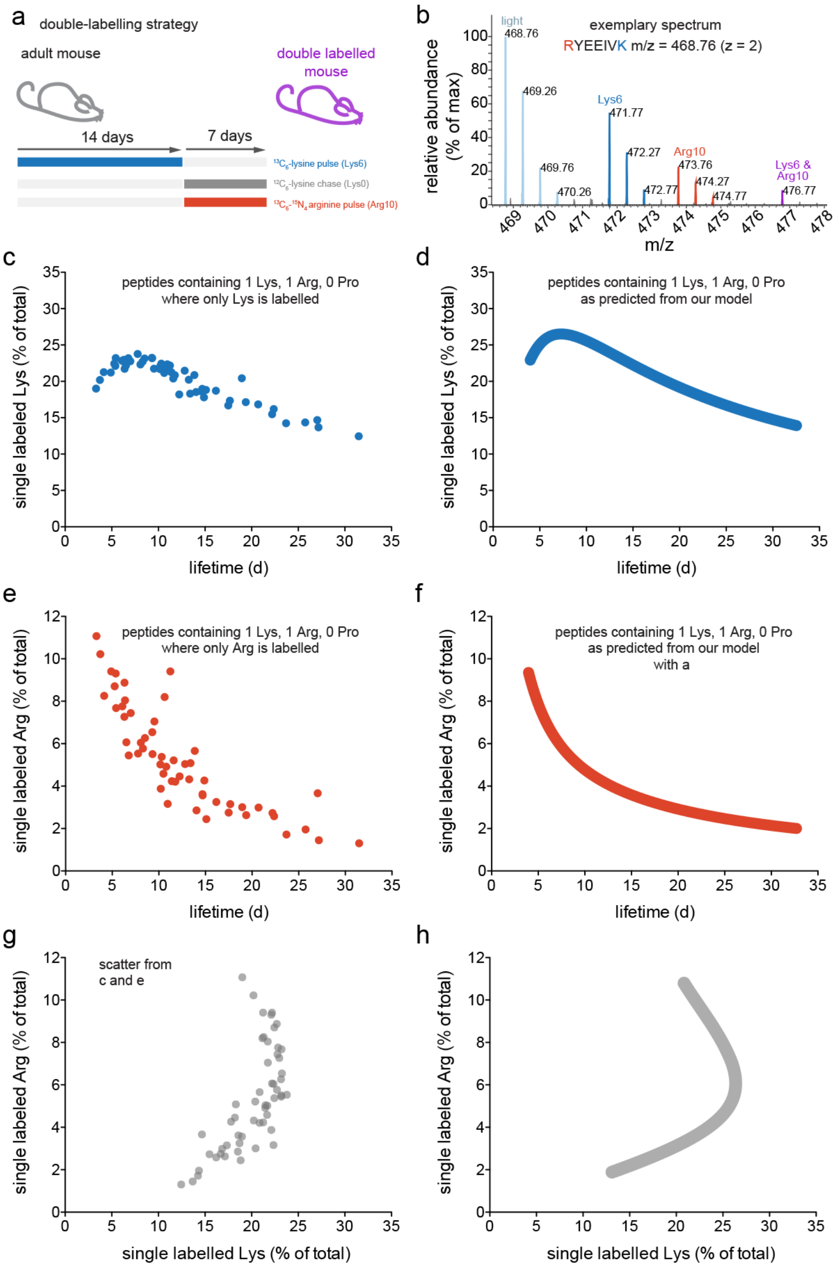

Supplementary Fig. 9 | An *in vivo* pulse-chase approach combined with a second pulse confirms the validity of the lifetime measurements. a, Double-labeling strategy used in the experiment. Mice were first pulsed for 14 days with Lys6 ($^{13}C_6$ lysine) and were then pulsed for 7 days with Lys0 ($^{12}C_6$ lysine). This chase was combined with the pulse of Arg10 ($^{13}C_6$-$^{15}N_4$ arginine). Please note that even if Arg is an amino acid whose dietary deprivation can cause growth deficits[4], it is not an essential amino acid and *in vivo* it can be metabolized to proline. Thus we restricted any analysis from this experiment to peptides containing one lysine and one arginine but devoid of proline. **b**, Exemplary spectrum for a peptide deriving from the *in vivo* labeling described in **a**. **c**, Scatter plot distribution of Lys6-labelling in the analyzed peptides where only lysine is labeled (Lys6-only; dark blue peaks in panel **b**) *versus* the lifetimes of the proteins determined as explained in **Supplementary Fig. 1, 3 and 5**. The percentage relates to the total amount of the respective peptides, taking into account all four possible labeling profiles (light, Lys6-only, Arg10-only, Lys6-Arg10; as exemplified in panel **b**). **d**, Predicted distribution of this labeling profile. Since we have not measured the pool of arginine, we estimated its labeling efficiency from optimizing the associated parameter b (1 / tau_{sol}) for the arginine model, while constraining all other parameters to those found in the lysine-only model. **e-f**, As in **a-b**, although in this case the Arg10-only containing peptides are shown (red peaks in panel **b**). **g**, Scatter plot deriving from the combination of panels **c** and **e** (measured labeling of single labeled Arginine on the y-axis and of single labeled Lysine on the x-axis). **h**, Predicted distribution of the data presented in **g**. The double pulse approach confirms once more the validity of the model used for the determination of the lifetimes.

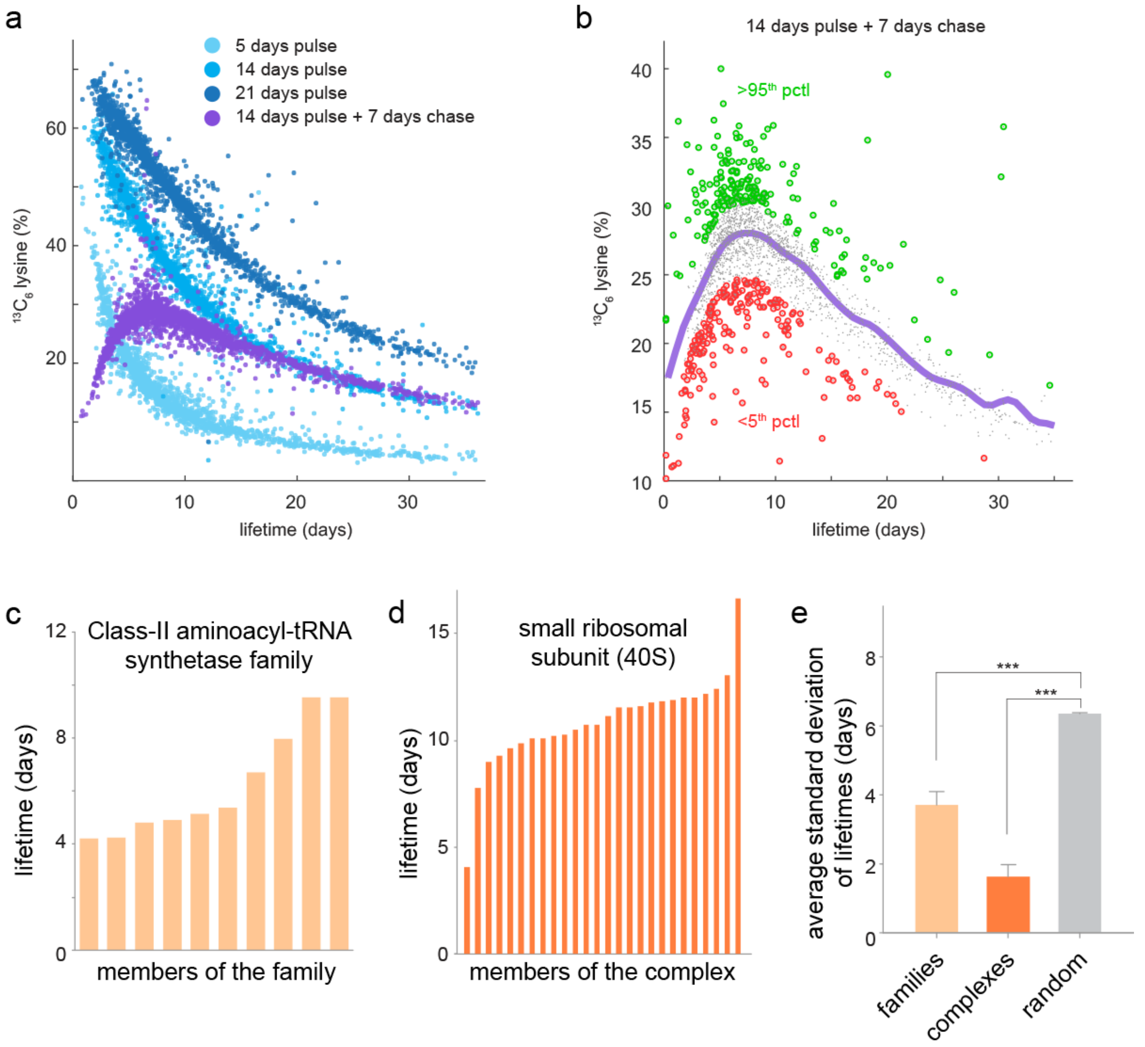

Supplementary Fig. 10 | Analysis of the pulse-chase data, and lifetimes in protein families and complexes. a, The pulse-chase data is represented in purple and is ordered by the lifetime measured from the pulse data, as in **Supplementary Fig. 3i**. Each data point corresponds to the average of the biological replicates for a particular protein. **b**, Zoom of the pulse-chase data from panel **a**. The purple line represents the weighted average behavior of the data, using Gaussian weighting (sigma = 2 days). In green we have highlighted the data with values higher than the 95th percentile (with the highest lysine incorporation in the chase) while in red the values lower than the 5th percentile (with the lowest lysine incorporation). We checked if the difference of incorporation for these values is significant, and we realized that there are no significantly different values with respect to the mean, and the data scattering corresponds to noise. In any case, we reasoned that some of the proteins in these two groups might behave differently than the rest of the proteome. We performed the gene ontology categorization of these two groups, and we found only a single biological process that is enriched in the lower 5th percentile (protein maturation, GO:0051604; including 12 proteins: O08915, P30999, P05132, P68181, P05480, Q61330, P13020, Q8BHG1, O08663, Q99JB2, Q9D924, Q9CYN9). This is a rather general protein category since GO:0051604 is related to "any process leading to the attainment of the full functional capacity of a protein". **c**, The lifetimes of proteins from the class-II aminoacyl-tRNA synthetase protein family. Albeit not identical, the lifetimes of the proteins in this family are all between 4 and 10 days. **d**, The lifetimes of proteins from the 40S (small) ribosomal subunit, as a representative macromolecular complex. **e**, We analyzed the coefficient of variation of the lifetimes in 119 protein families and macromolecular complexes, expressed as percentages of the coefficient of variation obtained when selecting random proteins. Data are presented as mean ± s.e.m.; n ≥ 20; *** = ANOVA *P*-value < 0.001. The coefficient of variation of the lifetimes is smaller than that of randomly selected proteins for both protein families and complexes, indicating that even if the lifetimes are not the same within these two groups, they are statistically closer to each other than a random group of proteins as might be expected.

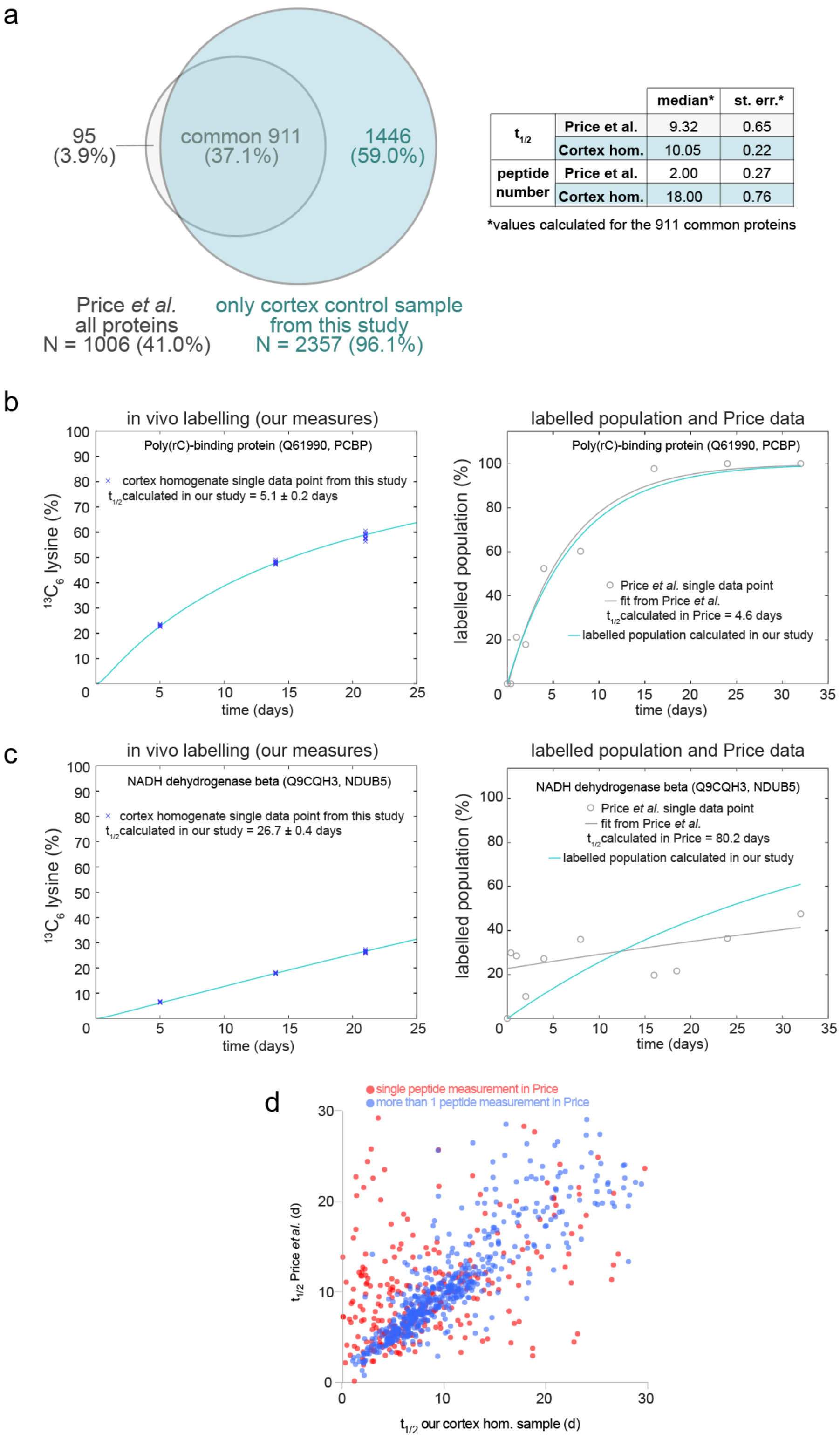

		median*	st. err.*
$t_{1/2}$	Price et al.	9.32	0.65
	Cortex hom.	10.05	0.22
peptide number	Price et al.	2.00	0.27
	Cortex hom.	18.00	0.76

*values calculated for the 911 common proteins

Supplementary Fig. 11 | Comparison between our brain cortex homogenate dataset and Price and collaborators[5]. a, Venn diagram and basic statistics of the proteins identified in the two datasets. **b**, A first example comparing the incorporation of $^{13}C_6$-lysine at different time points in one protein, in our own work (left), and the incorporation of ^{15}N labeling in the same protein, from Price and collaborators. For most proteins the measurements from the two studies are extremely similar, and the resulting lifetimes are also similar. **c**, Same as in **b**, showing one of the measurements where there is a substantial difference. Please note that in Price and collaborators the authors have decided in their fitting strategy to allow the population to have a variable delay in the emergence of the labeling (t_0) which results in inherently good fitting results (r^2), albeit this strategy is difficult to explain in physiological terms. **d**, Scatter plot showing the lifetimes of common proteins. The proteins that were determined with a single peptide in Price et al. are shown in red.

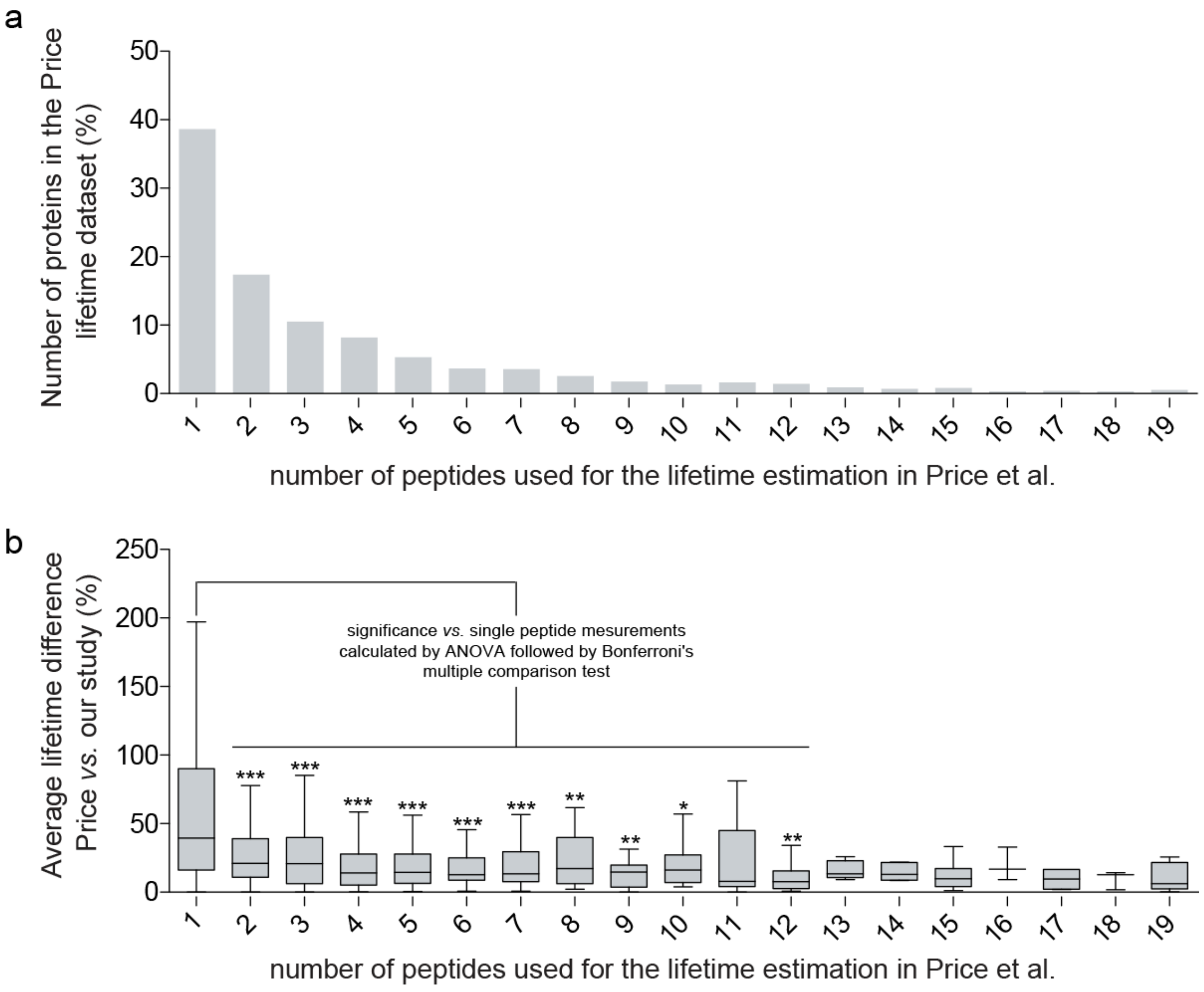

Supplementary Fig. 12 | Number of peptides used for the lifetime calculation in the dataset from Price and collaborators[5], and average difference between the two datasets depending on the number of peptides used in the measurements. a, Histogram of the number of protein lifetimes calculated in Price and collaborators, depending on the number of peptides used in the determination of the lifetimes. Note that for 37% of the proteins the lifetimes are calculated on a single peptide determination. **b**, Average lifetime difference between the two datasets (expressed as % of the respective lifetimes) subdivided for the peptide number used for the lifetime determination by Price *et al.* Note that the highest difference has been observed for the measures based on a single peptide in Price *et al.*, suggesting that the measurements based on at least two peptides are more consistent across different datasets. Adjusted *P*-values for ANOVA with Bonferroni *post hoc* test *vs.* single peptide (* ≤ 0.05, ** ≤ 0.01, *** ≤ 0.001).

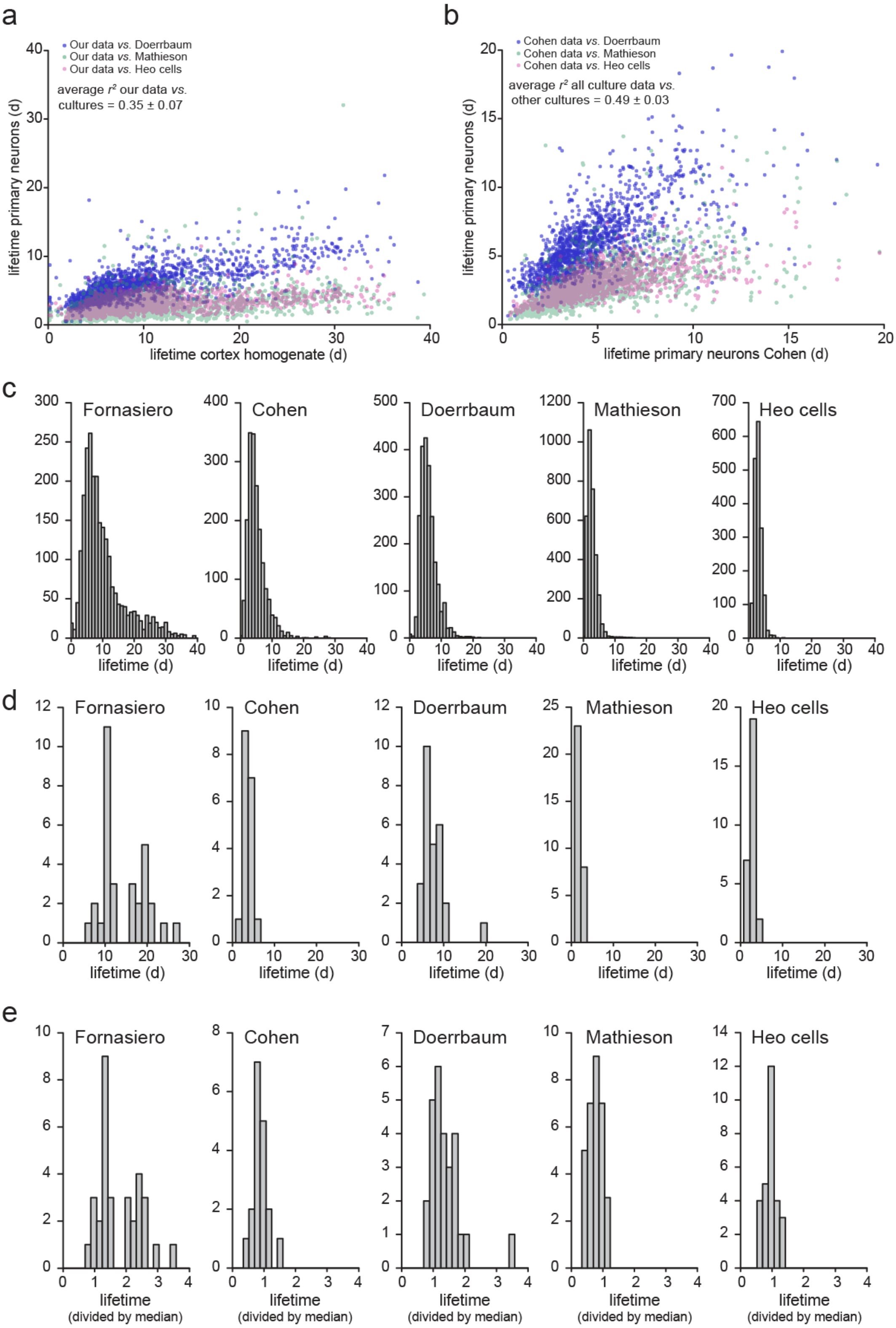

Supplementary Fig. 13 | Comparison between our brain cortex homogenate dataset and the *in vitro* results from other studies. a, Scatter plot comparing the lifetimes of proteins in vitro with our cortex in vivo data. The in vitro data come respectively from the Schuman laboratory (Dörrbaum et al., 2018)[6], the Savitski laboratory (Mathieson et al., 2018)[7] and the Huganir laboratory (Heo et al. 2018)[8]. **b**, Scatter plot comparing the in vitro data from the Ziv laboratory (Cohen et al., 2013)[9] with the other in vitro data introduced in panel **a**. **c**, Histograms of protein lifetimes in the different datasets showing the distribution of lifetimes. Note that in vitro all lifetimes are clustered together. **d**, Histograms of protein lifetimes in the different datasets for synaptic vesicle proteins. **e**, Same as in panel **e**, following median normalization (division of the vesicle protein lifetimes by the median of all measured lifetimes). The lifetimes measured in cultured are still all clustered tightly together.

a

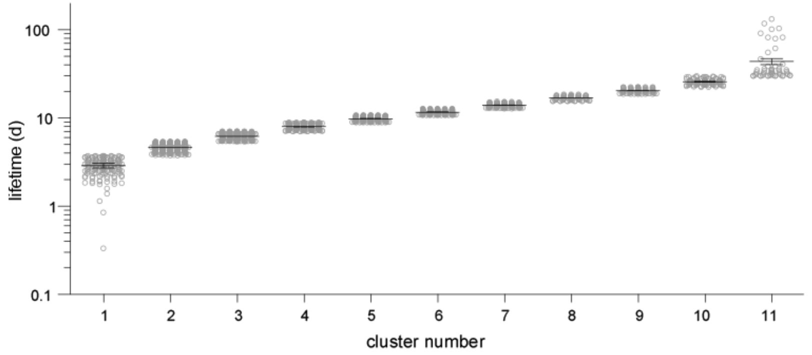

b

	Cluster features	lifetime boundaries	Cellular component (non redundant; FDR < 0.05)	-log10 p-value
Cluster 1	number of proteins = 156, mean t1/2 = 2.87 days	0.33 to 3.74 days	nuclear body (GO:0016604)	5.65
Cluster 2	number of proteins = 296, mean t1/2 = 4.67 days	3.74 to 5.41 days	nuclear envelope (GO:0005635)	3.62
Cluster 3	number of proteins = 345, mean t1/2 = 6.21 days	5.42 to 7.07 days	ER-Golgi intermediate compartment (GO:0005793)	3.71
			peptidase complex (GO:1905368)	3.27
			endoplasmic reticulum lumen (GO:0005788)	3.14
Cluster 4	number of proteins = 277, mean t1/2 = 7.98 days	7.08 to 8.92 days	——	—
Cluster 5	number of proteins = 213, mean t1/2 = 9.86 days	8.93 to 10.8 days	ribosome (GO:0005840)	5.50
			cytosolic part (GO:0044445)	3.42
Cluster 6	number of proteins = 173, mean t1/2 = 11.6 days	10.8 to 12.8 days	ribosome (GO:0005840)	9.10
			cytosolic part (GO:0044445)	8.47
			cell-substrate junction (GO:0030055)	4.16
			cell-cell adherens junction (GO:0005913)	2.83
Cluster 7	number of proteins = 117, mean t1/2 = 13.9 days	12.8 to 15.4 days	——	—
Cluster 8	number of proteins = 82, mean t1/2 = 16.8 days	15.4 to 18.5 days	——	—
Cluster 9	number of proteins = 97, mean t1/2 = 20.4 days	18.7 to 22.4 days	respiratory chain (GO:0070469)	6.32
			organelle inner membrane (GO:0019866)	6.21
			oxidoreductase complex (GO:1990204)	5.95
			mitochondrial protein complex (GO:0098798)	5.35
			mitochondrial membrane part (GO:0044455)	4.01
			plasma membrane protein complex (GO:0098797)	3.07
			transport vesicle (GO:0030133)	2.75
Cluster 10	number of proteins = 108, mean t1/2 = 25.8 days	22.5 to 29.8 days	organelle inner membrane (GO:0019866)	16.0
			mitochondrial membrane part (GO:0044455)	15.7
			mitochondrial protein complex (GO:0098798)	15.1
			respiratory chain (GO:0070469)	11.0
			mitochondrial matrix (GO:0005759)	8.50
			oxidoreductase complex (GO:1990204)	8.08
			myelin sheath (GO:0043209)	6.40
			cytochrome complex (GO:0070069)	5.01
			nucleoid (GO:0009295)	3.56
			outer membrane (GO:0019867)	3.21
Cluster 11	number of proteins = 54, mean t1/2 = 43.7 days	29.9 to 133 days	myelin sheath (GO:0043209)	9.60
			organelle inner membrane (GO:0019866)	6.56
			proton-transporting two-sector ATPase complex (GO:0016469)	5.65
			mitochondrial membrane part (GO:0044455)	4.87
			mitochondrial protein complex (GO:0098798)	4.55
			oxidoreductase complex (GO:1990204)	3.80
			intermediate filament cytoskeleton (GO:0045111)	3.35

c

	Cluster features	lifetime boundaries	Biological process (non redundant; FDR < 0.05)	-log10 p-value
Cluster 1	number of proteins = 156, mean t1/2 = 2.87 days	0.33 to 3.74 days	mRNA metabolic process (GO:0016071)	5.31
			translational initiation (GO:0006413)	5.03
			RNA splicing (GO:0008380)	3.93
			posttranscriptional regulation of gene expression (GO:0010608)	3.58
Cluster 2	number of proteins = 296, mean t1/2 = 4.67 days	3.74 to 5.41 days	——	—
Cluster 3	number of proteins = 345, mean t1/2 = 6.21 days	5.42 to 7.07 days	protein folding (GO:0006457)	6.83
Cluster 4	number of proteins = 277, mean t1/2 = 7.98 days	7.08 to 8.92 days	antigen processing and presentation (GO:0019882)	5.63
			signal release (GO:0023061)	4.63
			peptidyl-serine modification (GO:0018209)	4.08
			negative regulation of cell cycle (GO:0045786)	3.70
			amide transport (GO:0042886)	3.43
			positive regulation of secretion (GO:0051047)	3.25
			cell cycle phase transition (GO:0044770)	3.22
Cluster 5	number of proteins = 213, mean t1/2 = 9.86 days	8.93 to 10.8 days	single-organism carbohydrate metabolic process (GO:0044723)	4.16
			lamellipodium organization (GO:0097581)	3.84
Cluster 6	number of proteins = 173, mean t1/2 = 11.6 days	10.8 to 12.8 days	cellular aldehyde metabolic process (GO:0006081)	6.79
Cluster 7	number of proteins = 117, mean t1/2 = 13.9 days	12.8 to 15.4 days	——	—
Cluster 8	number of proteins = 82, mean t1/2 = 16.8 days	15.4 to 18.5 days	——	—
Cluster 9	number of proteins = 97, mean t1/2 = 20.4 days	18.7 to 22.4 days	modulation of synaptic transmission (GO:0050804)	4.36
Cluster 10	number of proteins = 108, mean t1/2 = 25.8 days	22.5 to 29.8 days	generation of precursor metabolites and energy (GO:0006091)	8.95
			tricarboxylic acid metabolic process (GO:0072350)	5.86
			mitochondrial membrane organization (GO:0007006)	5.68
			mitochondrial transport (GO:0006839)	3.92
			nucleoside triphosphate metabolic process (GO:0009141)	3.50
			hydrogen transport (GO:0006818)	3.42
			dicarboxylic acid metabolic process (GO:0043648)	3.33
Cluster 11	number of proteins = 54, mean t1/2 = 43.7 days	29.9 to 133 days	nucleoside triphosphate metabolic process (GO:0009141)	7.38
			nucleoside monophosphate metabolic process (GO:0009123)	6.82
			hydrogen transport (GO:0006818)	6.53
			glycosyl compound metabolic process (GO:1901657)	6.21
			ribose phosphate metabolic process (GO:0019693)	4.90
			purine-containing compound metabolic process (GO:0072521)	4.90
			ensheathment of neurons (GO:0007272)	4.56
			neural nucleus development (GO:0048857)	4.13
			axon development (GO:0061564)	4.12
			organophosphate biosynthetic process (GO:0090407)	3.85
			midbrain development (GO:0030901)	3.76
			monovalent inorganic cation transport (GO:0015672)	3.72
			generation of precursor metabolites and energy (GO:0006091)	3.15
			intermediate filament-based process (GO:0045103)	2.95

d

	Cluster features	lifetime boundaries	Molecular function (non redundant; FDR < 0.05)	-log10 p-value
Cluster 1	number of proteins = 156, mean t1/2 = 2.87 days	0.33 to 3.74 days	helicase activity (GO:0004386)	3.67
Cluster 2	number of proteins = 296, mean t1/2 = 4.67 days	3.74 to 5.41 days	——	—
Cluster 3	number of proteins = 345, mean t1/2 = 6.21 days	5.42 to 7.07 days	unfolded protein binding (GO:0051082)	4.18
Cluster 4	number of proteins = 277, mean t1/2 = 7.98 days	7.08 to 8.92 days	threonine-type peptidase activity (GO:0070003)	9.02
			protein serine/threonine kinase activity (GO:0004674)	4.93
			endopeptidase activity (GO:0004175)	3.41
Cluster 5	number of proteins = 213, mean t1/2 = 9.86 days	8.93 to 10.8 days	structural constituent of ribosome (GO:0003735)	5.06
Cluster 6	number of proteins = 173, mean t1/2 = 11.6 days	10.8 to 12.8 days	structural constituent of ribosome (GO:0003735)	10.6
			rRNA binding (GO:0019843)	5.29
Cluster 7	number of proteins = 117, mean t1/2 = 13.9 days	12.8 to 15.4 days	actin binding (GO:0003779)	4.12
Cluster 8	number of proteins = 82, mean t1/2 = 16.8 days	15.4 to 18.5 days	——	—
Cluster 9	number of proteins = 97, mean t1/2 = 20.4 days	18.7 to 22.4 days	oxidoreductase activity, acting on NAD(P)H (GO:0016651)	5.68
			metal cluster binding (GO:0051540)	4.62
Cluster 10	number of proteins = 108, mean t1/2 = 25.8 days	22.5 to 29.8 days	structural constituent of cytoskeleton (GO:0005200)	4.20
			heme-copper terminal oxidase activity (GO:0015002)	3.83
			oxidoreductase activity (GO:0016675)	3.48
Cluster 11	number of proteins = 54, mean t1/2 = 43.7 days	29.9 to 133 days	inorganic cation transmembrane transporter (GO:0022890)	4.69
			active transmembrane transporter activity (GO:0022804)	4.17

Supplementary Fig. 14 | Fuzzy c-means clustering of the protein lifetimes and functional enrichment analysis of the clusters. a, The lifetimes were divided in classes with the fuzzy c-means algorithm[10]. The

Supplementary Fig. 14 legend continuing from the previous page:
optimal number of clusters was determined to be 11, based on the negligible improvement (<1%) of the within-cluster sum of squares by increasing the number of clusters over 11. Clusters have been ordered by increasing lifetimes, with Cluster 1 corresponding to the lowest and Cluster 11 to the highest lifetimes. **b-d**, Summary of the functional analysis for all the 11 clusters, detailing the cluster features (number of proteins, mean lifetime and lifetime boundaries). The analysis was performed with WebGestalt 2017[11]. Only non-redundant significant terms are represented (with a false discovery rate lower than 0.05). The *P*-values are reported as $-\log_{10}$ (where the higher numbers color-coded in darker shades of red correspond to the most significant terms). In brief, Cluster 1, which has the shortest lifetimes, corresponds to nuclear proteins implicated in mRNA metabolism and translation. Cluster 2 contains some proteins that are part of the nuclear envelope. Cluster 3 is implicated in protein folding and is linked to ER and ER-Golgi transition functional processes. Cluster 4 is related to protein production and intracellular signaling. Cluster 5 includes several ribosomal proteins and is linked to adhesion and carbohydrate metabolism. Cluster 6 has similar features to Cluster 5. Cluster 7 is implicated in actin binding. Cluster 8 is not enriched for any term (for any cellular pathway). Cluster 9 includes terms implicated in the modulation of synaptic transmission and in mitochondrial metabolism. Cluster 10 is clearly mitochondrial. Cluster 11, the most stable, includes several mitochondrial processes, axon and neuron development, myelin components and neuronal nuclear development. This analysis is in good agreement with the specific lifetime differences reported in **Fig. 1** and **2**.

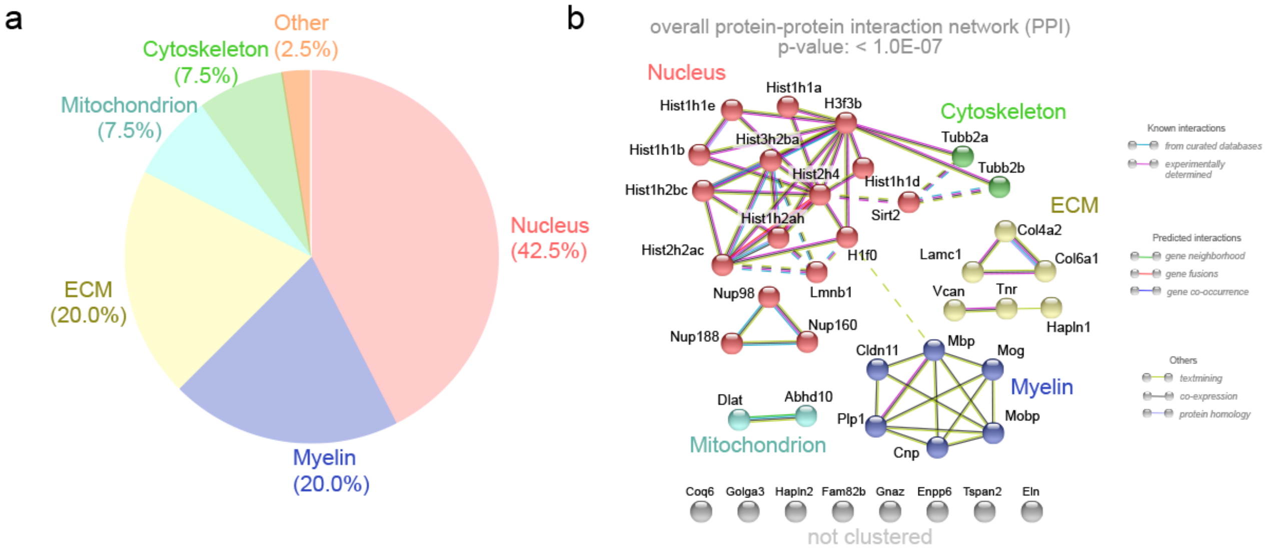

c

Cellular component	-log10 FDR
nucleosome (GO:0000786)	16.41
protein complex (GO:0043234)	7.52
chromatin (GO:0000785)	6.67
myelin sheath (GO:0043209)	6.47
chromosomal part (GO:0044427)	5.80
proteinaceous extracellular matrix (GO:0005578)	4.94
macromolecular complex (GO:0032991)	4.86
extracellular region part (GO:0044421)	4.86
nuclear chromatin (GO:0000790)	4.62
nuclear chromosome (GO:0000228)	4.45
extracellular region (GO:0005576)	3.96
extracellular exosome (GO:0070062)	3.20
nuclear euchromatin (GO:0005719)	3.10
extracellular matrix component (GO:0044420)	2.51
nuclear pore (GO:0005643)	2.30
membrane-bounded organelle (GO:0043227)	2.18
nuclear pore outer ring (GO:0031080)	2.08
microtubule (GO:0005874)	1.82
nuclear nucleosome (GO:0000788)	1.66
nuclear part (GO:0044428)	1.61
intracellular membrane-bounded organelle (GO:0043231)	1.61

d

Biological process	-log10 FDR
nucleosome assembly (GO:0006334)	6.47
protein complex assembly (GO:0006461)	6.02
protein complex biogenesis (GO:0070271)	6.02
cellular macromolecular complex assembly (GO:0034622)	4.87
protein complex subunit organization (GO:0071822)	3.77
chromatin assembly (GO:0031497)	3.27
macromolecular complex assembly (GO:0065003)	3.27
substantia nigra development (GO:0021762)	3.25
nucleosome organization (GO:0034728)	3.22
cellular component assembly (GO:0022607)	3.20
DNA packaging (GO:0006323)	2.82
cellular component biogenesis (GO:0044085)	2.82
midbrain development (GO:0030901)	2.25
axon ensheathment (GO:0008366)	2.09

e

Molecular function	-log10 FDR
structural molecule activity (GO:0005198)	4.98
chromatin DNA binding (GO:0031490)	2.33
hyaluronic acid binding (GO:0005540)	2.11
extracellular matrix structural constituent (GO:0005201)	1.83
structural constituent of myelin sheath (GO:0019911)	1.83

Supplementary Fig. 15 | Classification and string analysis of the Extremely Long Lived Proteins (ELLPs). a, Pie chart representation of the classification of ELLPs (corresponding to the 98th-100th percentile of the most stabilized proteins). The majority of ELLPs are nuclear. There is also a clear over-representation of myelin and extracellular matrix proteins. **b**, String analysis[12] of ELLPs reveals a large nuclear cluster including histones and Nups, as well as a myelin and an ECM cluster, confirming the distribution represented in **a**. **c-e**, Detailed functional classification of ELLPs, deriving from the string analysis confirms the overrepresentation of nucleosomes, myelin, extracellular matrix, nuclear pore and the related biological processes.

a

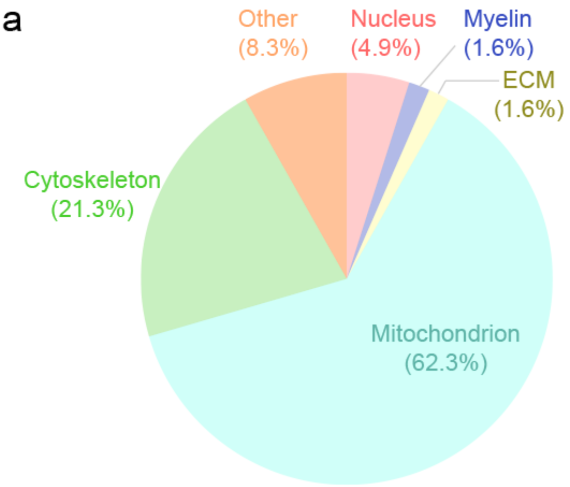

b

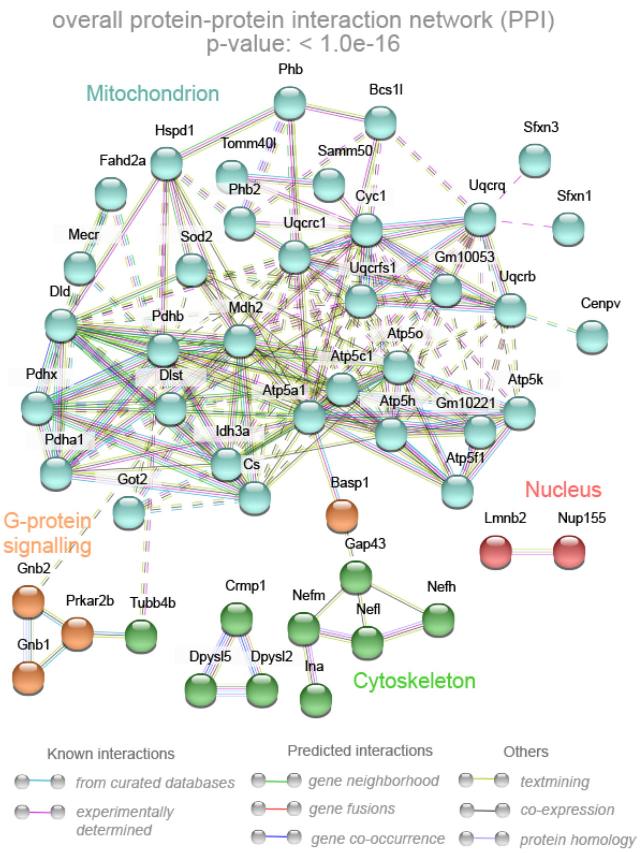

c

Molecular function	-log10 FDR
hydrogen ion transmembrane transporter activity (GO:0015078)	6.85
structural molecule activity (GO:0005198)	3.41
proton-transporting ATP synthase activity, rotational mechanism (GO	3.16
transmembrane transporter activity (GO:0022857)	2.46
transporter activity (GO:0005215)	2.44
ion transmembrane transporter activity (GO:0015075)	2.44
substrate-specific transporter activity (GO:0022892)	2.44
catalytic activity (GO:0003824)	2.44
pyruvate dehydrogenase (acetyl-transferring) activity (GO:0004739)	2.18
ubiquinol-cytochrome-c reductase activity (GO:0008121)	1.74
nucleoside-triphosphatase activity (GO:0017111)	1.36

d

Cellular component	-log10 FDR
myelin sheath (GO:0043209)	30.60
mitochondrion (GO:0005739)	14.75
mitochondrial part (GO:0044429)	14.42
mitochondrial membrane (GO:0031966)	12.45
mitochondrial envelope (GO:0005740)	12.21
organelle envelope (GO:0031967)	12.21
organelle inner membrane (GO:0019866)	12.08
mitochondrial inner membrane (GO:0005743)	11.43
mitochondrial proton-transporting ATP synthase complex, coupling fa	8.98
extracellular exosome (GO:0070062)	7.05
protein complex (GO:0043234)	6.80
oxidoreductase complex (GO:1990204)	6.67
membrane-bounded vesicle (GO:0031988)	6.65
mitochondrial membrane part (GO:0044455)	6.56
neurofilament (GO:0005883)	6.37
extracellular region part (GO:0044421)	6.04
mitochondrial protein complex (GO:0098798)	5.92
organelle membrane (GO:0031090)	5.69
respiratory chain (GO:0070469)	5.54
inner mitochondrial membrane protein complex (GO:0098800)	5.40
extracellular region (GO:0005576)	4.78
mitochondrial respiratory chain complex III (GO:0005750)	4.71
mitochondrial matrix (GO:0005759)	4.57
intracellular organelle part (GO:0044446)	4.54
pyruvate dehydrogenase complex (GO:0045254)	4.52
mitochondrial proton-transporting ATP synthase complex (GO:00057	4.00
organelle part (GO:0044422)	3.73
cytoplasmic part (GO:0044444)	3.53
intermediate filament (GO:0005882)	3.40
cytoskeletal part (GO:0044430)	3.39

e

Biological process	-log10 FDR
ATP metabolic process (GO:0046034)	11.80
cellular respiration (GO:0045333)	11.32
ATP synthesis coupled proton transport (GO:0015986)	10.22
nucleotide metabolic process (GO:0009117)	9.61
organophosphate metabolic process (GO:0019637)	7.73
tricarboxylic acid cycle (GO:0006099)	7.70
generation of precursor metabolites and energy (GO:0006091)	7.40
hydrogen ion transmembrane transport (GO:1902600)	6.33
small molecule metabolic process (GO:0044281)	6.24
aerobic respiration (GO:0009060)	5.97
neurofilament cytoskeleton organization (GO:0060052)	5.57
oxidation-reduction process (GO:0055114)	5.32
carbohydrate derivative metabolic process (GO:1901135)	5.12
respiratory electron transport chain (GO:0022904)	4.51
intermediate filament bundle assembly (GO:0045110)	4.38
organonitrogen compound metabolic process (GO:1901564)	4.19
acetyl-CoA metabolic process (GO:0006084)	4.15
mitochondrial ATP synthesis coupled electron transport (GO:004277	4.15
acetyl-CoA biosynthetic process from pyruvate (GO:0006086)	4.11
single-organism biosynthetic process (GO:0044711)	4.07
single-organism metabolic process (GO:0044710)	3.92
mitochondrial electron transport, ubiquinol to cytochrome c (GO:0006	3.54
oxidative phosphorylation (GO:0006119)	3.52
organophosphate biosynthetic process (GO:0090407)	3.38
neuron projection morphogenesis (GO:0048812)	3.32
neuron development (GO:0048666)	3.12

Supplementary Fig. 16 | Classification and string analysis of the Long Lived Proteins (LLPs). a, Pie chart representation of the classification of LLPs (corresponding to the 95th-98thpercentile of the most stabilized proteins). The majority of LLPs are mitochondrial. There is also a clear over-representation of cytoskeletal proteins. **b**, String analysis[12] of LLPs reveals a large nuclear cluster including mitochondrial proteins, as well as a cytoskeletal cluster, confirming the distribution represented in **a**. **c-e**, Detailed functional classification of LLPs, deriving from the string analysis indicates an overrepresentation of the mitochondrial and of the cytoskeletal clusters (neurofilaments), and it also detects that among these proteins there are components of myelin, extracellular matrix and extracellular exosomes.

a

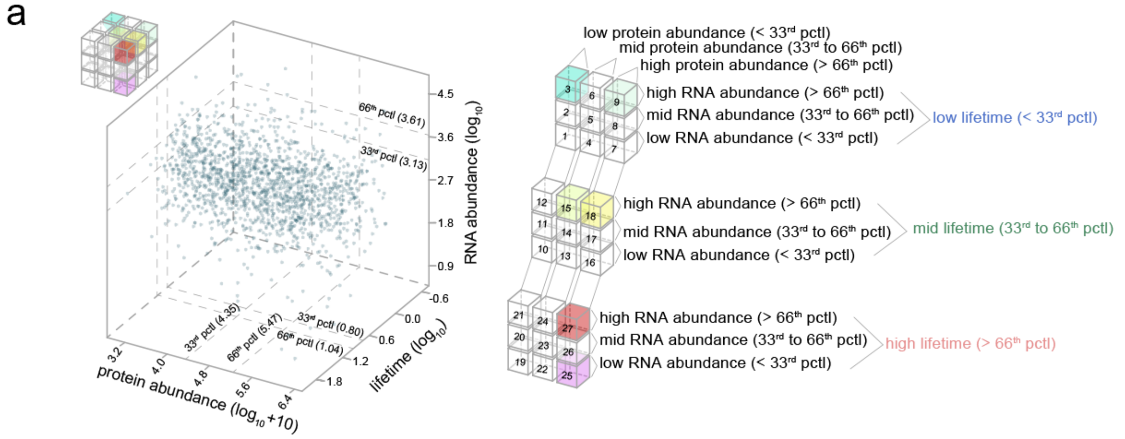

b

Group number	lifetime	protein abundance	mRNA abundance	Biological process FDR < 0.05	Biological process p-val < 0.001	Cellular component FDR < 0.05	Cellular component p-val < 0.001	Molecular function FDR < 0.05	Molecular function p-val < 0.001
1	low	low	low	---	1	1	1	---	---
2	low	low	medium	---	1	---	---	---	---
3	low	low	high	2	4	---	1	1	1
4	low	medium	low	1	1	---	---	---	1
5	low	medium	medium	1	2	1	1	---	---
6	low	medium	high	---	1	---	---	---	---
7	low	high	low	---	1	---	---	---	---
8	low	high	medium	---	---	1	1	---	---
9	low	high	high	---	3	5	5	---	2
10	medium	low	low	---	---	1	2	---	---
11	medium	low	medium	---	---	1	1	---	---
12	medium	low	high	---	---	1	1	---	---
13	medium	medium	low	---	2	---	---	---	---
14	medium	medium	medium	---	---	---	---	---	---
15	medium	medium	high	5	6	---	---	1	1
16	medium	high	low	---	1	1	1	---	1
17	medium	high	medium	---	1	---	---	---	---
18	medium	high	high	---	4	6	4	---	---
19	high	low	low	---	---	---	---	---	---
20	high	low	medium	---	1	---	---	---	---
21	high	low	high	---	1	---	---	---	---
22	high	medium	low	---	1	---	1	---	---
23	high	medium	medium	---	---	---	---	---	---
24	high	medium	high	---	2	---	2	---	---
25	high	high	low	---	---	8	7	2	3
26	high	high	medium	1	3	7	10	---	1
27	high	high	high	16	21	12	9	6	8

c

group 3 (low lifetime, low protein, high mRNA)	-log10 p-value
protein polyubiquitination (GO:0000209)	3.84
cellular response to lipid (GO:0071396)	3.84
response to radiation (GO:0009314)	3.40
positive regulation of GTPase activity (GO:0043547)	3.17
chromatin (GO:0000785)	3.27
ubiquitin-like protein transferase activity (GO:0019787)	3.61

group 9 (low lifetime, high protein, high mRNA)	-log10 p-value
regulation of gene expression (GO:0010608)	3.77
multi-organism cellular process (GO:0044764)	3.15
translational elongation (GO:0006414)	3.09
microtubule associated complex (GO:0005875)	6.43
microtubule (GO:0005874)	4.26
autophagosome (GO:0005776)	3.08
late endosome (GO:0005770)	3.01
unfolded protein binding (GO:0051082)	3.39
ribonucleoprotein complex binding (GO:0043021)	3.08

group 15 (med. lifetime, med. protein, high mRNA)	-log10 p-value
peptidyl-serine modification (GO:0018209)	4.96
animal organ formation (GO:0048645)	4.25
gastrulation (GO:0007369)	3.69
vesicle-mediated transport in synapse (GO:0099003)	3.48
negative regulation of cell cycle (GO:0045786)	3.41
neuron migration (GO:0001764)	3.01
protein serine/threonine kinase activity (GO:0004674)	3.68

group 18 (high lifetime, high protein, low mRNA)	-log10 p-value
pyridine-compound metabolic process (GO:0072524)	3.76
pyruvate metabolic process (GO:0006090)	3.12
nucleoside diphosphate metabolic process (GO:0009132)	3.03
regulation of DNA metabolic process (GO:0051052)	3.02
myelin sheath (GO:0043209)	5.80
neuron projection terminus (GO:0044306)	4.58
vacuolar part (GO:0044437)	3.50

group 25 (high lifetime, high protein, low mRNA)	-log10 p-value
ribosome (GO:0005840)	6.77
organelle inner membrane (GO:0019866)	5.30
cytosolic part (GO:0044445)	3.68
mitochondrial protein complex (GO:0098798)	3.36
mitochondrial matrix (GO:0005759)	3.18
structural constituent of ribosome (GO:0003735)	5.86
rRNA binding (GO:0019843)	4.75

group 27 (high lifetime, high protein, high mRNA)	-log10 p-value
generation of metabolites and energy (GO:0006091)	6.86
hydrogen transport (GO:0006818)	6.28
tricarboxylic acid metabolic process (GO:0072350)	6.22
nucleoside triphosphate metabolic process (GO:0009141)	5.77
nucleoside monophosphate metab. process (GO:0009123)	5.38
monovalent inorganic cation transport (GO:0015672)	4.96
ATP hydrolysis coupled TM transport (GO:0090662)	4.80
glycosyl compound metabolic process (GO:1901657)	4.58
ribose phosphate metabolic process (GO:0019693)	4.57
purine-compound metabolic process (GO:0072521)	4.50
monovalent inorganic cation homeostasis (GO:0055067)	4.12
midbrain development (GO:0030901)	3.94
organic acid transport (GO:0015849)	3.89
import into cell (GO:0098657)	3.70
anion transport (GO:0006820)	3.63
neural nucleus development (GO:0048857)	3.41
myelin sheath (GO:0043209)	16.00
organelle inner membrane (GO:0019866)	4.97
axon (GO:0030424)	3.72
oxidoreductase complex (GO:1990204)	3.56
respiratory chain (GO:0070469)	3.52
mitochondrial protein complex (GO:0098798)	3.30
cytochrome complex (GO:0070069)	3.10
active transmembrane transporter activity (GO:0022804)	8.12
inorganic cation TM transporter activity (GO:0022890)	6.20
structural constituent of cytoskeleton (GO:0005200)	4.76
organic acid TM transporter activity (GO:0005342)	3.42
anion transmembrane transporter activity (GO:0008509)	3.26
cofactor binding (GO:0048037)	3.15

Supplementary Fig. 17 | Clustering of protein lifetimes based on protein and mRNA level quantifications. a, 3D scatter of protein lifetimes versus protein and mRNA abundance obtained as detailed

Supplementary Fig. 17 legend continuing from the previous page:
in the Online Methods. The data was subdivided in 27 groups of proteins depending on the percentile distribution of these proteins in three dimensions (protein lifetime, mRNA abundance and protein abundance), as schematized on the right side of the graph. This results in 27 groups or clusters, ranging from "low protein abundance, low mRNA abundance, and low lifetime" to "high protein abundance, high mRNA abundance, and high lifetime". **b**, Table resuming the results of the functional enrichment analysis of the 27 clusters[11]. Some of the most interesting results (further detailed in panel **c**) are highlighted in color. **c**, Detailed functional analysis for the groups highlighted in **b**. Group 3, which corresponds to proteins with a low abundance and a short lifetime that have relatively high mRNA levels, includes regulatory proteins that presumably need to be fast produced on demand, when needed. Group 9 corresponds to proteins that are abundant and have high mRNA levels, but have short lifetimes, and is functionally associated with auto-phagosomal function, late endosomes and microtubule turnover. Group 15, with a medium lifetime and protein abundance but high mRNA is linked to protein signaling, vesicle transport and several developmental processes. Group 18. with long lifetimes, abundant proteins but with low mRNAs, includes processes associated with nucleotide metabolism and regulation of DNA synthesis. Group 25, with long lifetimes, abundant proteins, but low mRNA levels, is associated with the ribosome and with some specific mitochondrial functions. There are also several biological processes that require high mRNA and protein abundance, coupled to long lifetimes (group 27). These include mitochondrial functions and brain components such as axon and myelin structural proteins.

a

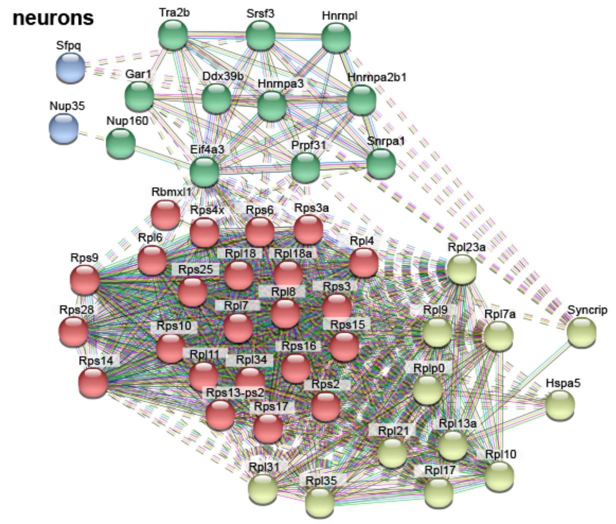

	GO category	-log10 FDR
String analysis Biological Process	translation (GO.0006412)	24.33
	gene expression (GO.0010467)	16.85
	cellular nitrogen compound metabolic process (GO.0034641)	14.13
	RNA processing (GO.0006396)	12.58
	cellular macromolecule metabolic process (GO.0044260)	11.97
	macromolecule metabolic process (GO.0043170)	10.72
	ribonucleoprotein complex biogenesis (GO.0022613)	8.95
	cellular macromolecule biosynthetic process (GO.0034645)	8.62
	cellular protein metabolic process (GO.0044267)	8.58
String analysis Molecular Function	structural constituent of ribosome (GO.0003735)	33.83
	poly(A) RNA binding (GO.0044822)	33.34
	RNA binding (GO.0003723)	31.05
	nucleic acid binding (GO.0003676)	17.95
	heterocyclic compound binding (GO.1901363)	8.95
	organic cyclic compound binding (GO.0097159)	8.86
	rRNA binding (GO.0019843)	8.75
	mRNA binding (GO.0003729)	8.71
	binding (GO.0005488)	2.24
String analysis Cellular Component	cytosolic ribosome (GO.0022626)	42.67
	ribonucleoprotein complex (GO.0030529)	38.36
	ribosomal subunit (GO.0044391)	37.42
	ribosome (GO.0005840)	35.65
	cytosolic large ribosomal subunit (GO.0022625)	29.27
	large ribosomal subunit (GO.0015934)	24.92
	focal adhesion (GO.0005925)	19.06
	macromolecular complex (GO.0032991)	15.52
	nuclear part (GO.0044428)	14.77

b

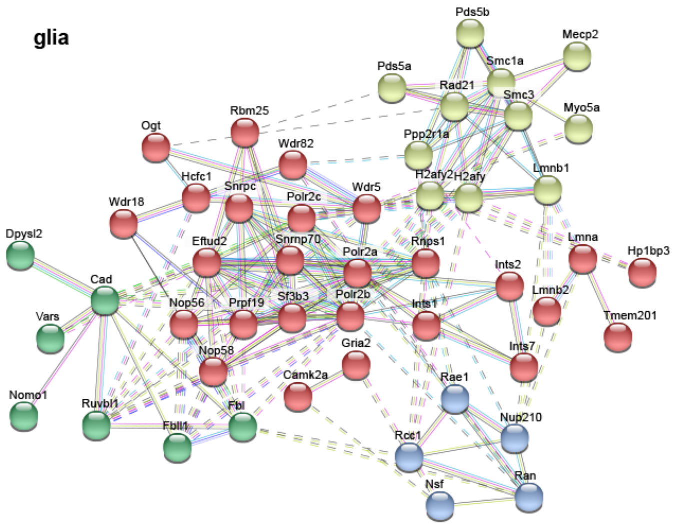

	GO category	-log10 FDR
String analysis Biological Process	nucleic acid metabolic process (GO.0090304)	6.66
	RNA metabolic process (GO.0016070)	6.64
	RNA processing (GO.0006396)	6.38
	cellular aromatic compound metabolic process (GO.0006725)	6.38
	gene expression (GO.0010467)	6.07
	organic cyclic compound metabolic process (GO.1901360)	6.07
	mitotic nuclear division (GO.0007067)	5.23
	rRNA modification (GO.0000154)	3.95
	cell cycle process (GO.0022402)	3.74
String analysis Molecular Function	nucleic acid binding (GO.0003676)	4.66
	chromatin binding (GO.0003682)	4.66
	RNA binding (GO.0003723)	4.66
	poly(A) RNA binding (GO.0044822)	4.64
	macromolecular complex binding (GO.0044877)	3.72
	heterocyclic compound binding (GO.1901363)	3.53
	organic cyclic compound binding (GO.0097159)	3.49
	histone acetyltransferase activity (H4-K5 specific) (GO.0043995)	3.32
	histone acetyltransferase activity (H4-K8 specific) (GO.0043996)	3.32
String analysis Cellular Component	nucleoplasm (GO.0005654)	19.52
	nuclear part (GO.0044428)	17.03
	nuclear lumen (GO.0031981)	16.58
	nucleoplasm part (GO.0044451)	15.70
	nucleus (GO.0005634)	12.61
	macromolecular complex (GO.0032991)	10.01
	intracellular organelle part (GO.0044446)	9.89
	intracellular membrane-bounded organelle (GO.0043231)	9.56
	membrane-bounded organelle (GO.0043227)	8.59

Supplementary Fig. 18 | Detailed string analysis categorization of the proteins with differentially regulated lifetimes in Glia and neuron nuclei. a, String analysis representation[12] of the proteins significantly longer-lived in neuronal nuclei ($NeuN^+$) with respect to Glia nuclei ($NeuN^-$). Only proteins with an adjusted $P < 0.001$ were included in the analysis. K-mean clustering of the identified clusters reveals two large clusters of ribosomal proteins corresponding to ribosomes enriched in the nuclear envelope (red and yellow, as also detailed in the right side of the panel). The green and the blue clusters include several mRNA binding proteins and nuclear components. **b**, As in **a** for the proteins longer lived in Glial nuclei ($NeuN^-$). The largest cluster (red) includes RNA polymerase II components and several regulators of mRNA splicing. In glial cells there is a less clear functional association of the proteins that seem to be preferentially stabilized (as seen from the lower number of links in the cluster graph), although the study of the specific pathways highlighted here might reveal specific regulatory mechanisms.

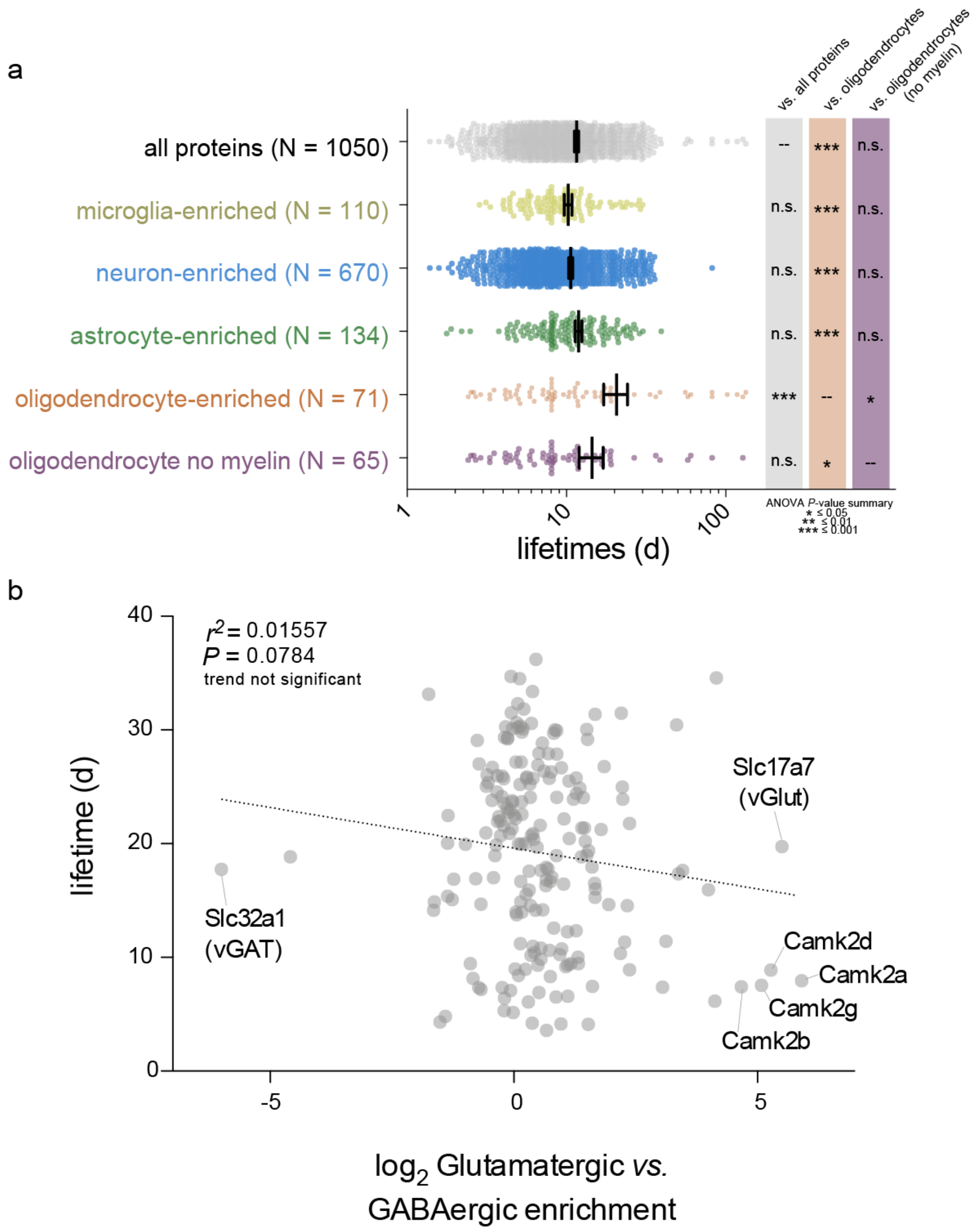

Supplementary Fig. 19 | Protein lifetimes in different brain cell subtypes. a, Protein lifetimes grouped in accordance to their enrichment in different cellular populations of the brain, as defined elsewhere[13]. We considered a protein enriched in a cell type when its amounts were >10-fold more abundant in one cell type compared with all the others cell types (following the same principle that was used by Sharma and collaborators[13]). Each data point corresponds to a single protein lifetime, and the black lines indicate the mean and the standard error of the mean (SEM) for each group. Oligodendrocytes are the only cell type that with a significantly higher mean lifetime (*** = *P*-value < 0.001). This difference is accountable to the fact that these cells produce myelin, which is an extremely long-lived structure in the brain. If the proteins that are characteristic for myelin are excluded, the mean protein lifetime of Oligodendrocytes is not significantly longer than the one of the other cell populations (see the ANOVA P-value summary on the right side of the panel for significances). **b**, Protein lifetimes in glutamatergic *vs.* GABAergic neurons. For the specificity of the glutamatergic/Gabaergic enrichment we relied on data published elsewhere[14]. The data is represented as a scatter plot where the log2 ratio of the enrichment has a positive value on the x-axis when the protein is more abundant in the glutamatergic population vs. the GABAergic one. The specific GABAergic and glutamatergic vesicular transporters (respectively vGlut and vGAT) are highlighted. There is trend for the Glutamatergic population of neurons to have shorter lifetimes (not statistically significant), but it is only due to the high abundance of CamK subunits in these neurons, which are short-lived (also detailed in the graph).

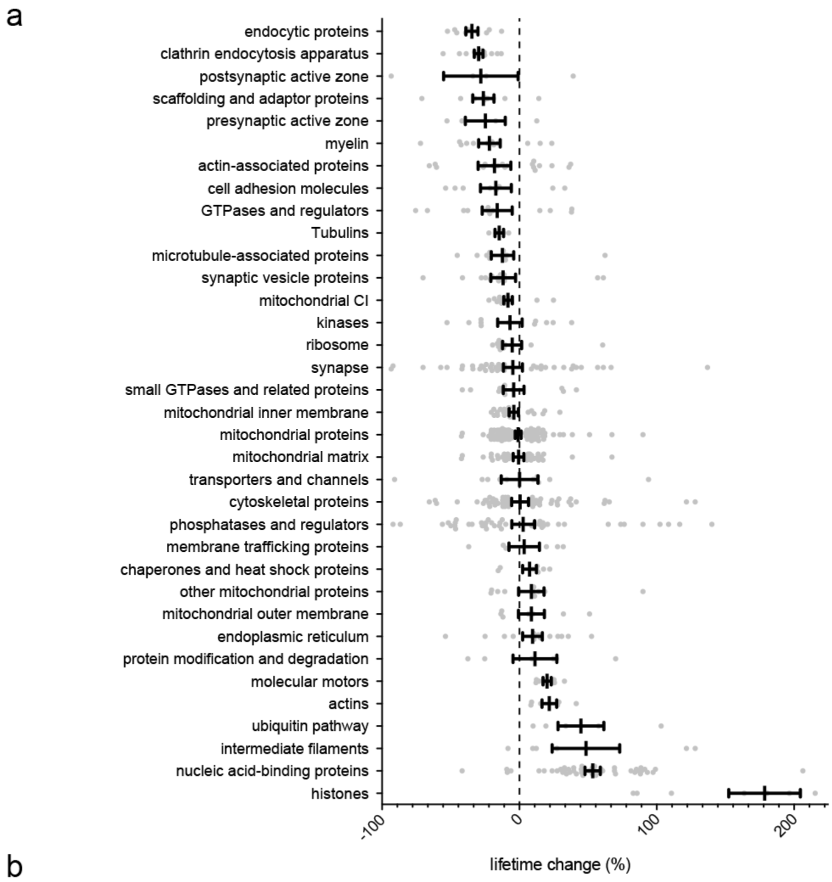

b

higher lifetimes in the brain cortex vs. cerebellum

Biological process (non redundant; FDR < 0.05)	-log10 *P*-value
protein localization to membrane (GO:0072657)	5.43
receptor-mediated endocytosis (GO:0006898)	5.35
protein localization to cell periphery (GO:1990778)	4.79
endomembrane system organization (GO:0010256)	4.34
cell morphogenesis involved in neuron diff. (GO:0048667)	4.14
regulation of cell-cell adhesion (GO:0022407)	4.14
receptor metabolic process (GO:0043112)	4.00
cell junction organization (GO:0034330)	3.75
negative regulation of transport (GO:0051051)	3.54
membrane biogenesis (GO:0044091)	3.50
cell-cell adhesion via plasma-membrane (GO:0098742)	3.50
leukocyte cell-cell adhesion (GO:0007159)	3.44
regulation of embryonic development (GO:0045995)	3.14

Cellular component (non redundant; FDR < 0.05)	-log10 *P*-value
membrane region (GO:0098589)	12.20
coated membrane (GO:0048475)	4.90
plasma membrane protein complex (GO:0098797)	4.84
secretory vesicle (GO:0099503)	4.68
cell leading edge (GO:0031252)	4.60
axon (GO:0030424)	4.51
basolateral plasma membrane (GO:0016323)	4.16
anchored component of membrane (GO:0031225)	3.95
dendrite (GO:0030425)	3.52
presynapse (GO:0098793)	3.40
cell projection membrane (GO:0031253)	2.96
neuron to neuron synapse (GO:0098984)	2.95
occluding junction (GO:0070160)	2.90
postsynapse (GO:0098794)	2.89
transport vesicle (GO:0030133)	2.75
vesicle membrane (GO:0012506)	2.53
apical junction complex (GO:0043296)	2.41

higher lifetimes in the cerebellum vs. brain cortex

Biological process (non redundant; FDR < 0.05)	-log10 *P*-value
RNA splicing (GO:0008380)	>20
mRNA metabolic process (GO:0016071)	>20
chromatin assembly or disassembly (GO:0006333)	8.23
chromosome organization (GO:0051276)	6.94
protein-DNA complex subunit organization (GO:0071824)	6.58
covalent chromatin modification (GO:0016569)	5.81
peptidyl-lysine modification (GO:0018205)	5.61
chromatin remodeling (GO:0006338)	5.08
methylation (GO:0032259)	4.36
RNA localization (GO:0006403)	3.35
nucleobase-containing compound transport (GO:0015931)	3.35
rhythmic process (GO:0048511)	3.35
protein alkylation (GO:0008213)	3.33
ribonucleoprotein complex subunit organ. (GO:0071826)	3.08
DNA repair (GO:0006281)	3.02
ribonucleoprotein complex biogenesis (GO:0022613)	2.98
gene silencing (GO:0016458)	2.91

Cellular component (non redundant; FDR < 0.05)	-log10 *P*-value
spliceosomal complex (GO:0005681)	>20
chromatin (GO:0000785)	15.11
nuclear body (GO:0016604)	14.70
nuclear chromosome (GO:0000228)	10.94
DNA packaging complex (GO:0044815)	7.42
methyltransferase complex (GO:0034708)	5.69
protein-DNA complex (GO:0032993)	5.29
small nuclear ribonucleoprotein complex (GO:0030532)	5.02
nuclear periphery (GO:0034399)	3.86
ribonucleoprotein granule (GO:0035770)	2.89
exon-exon junction complex (GO:0035145)	2.60
SWI/SNF superfamily-type complex (GO:0070603)	2.60
transcription factor complex (GO:0005667)	2.44

Supplementary Fig. 20 | Protein lifetime changes in the cerebellum *versus* the brain cortex, organized by protein groups. a, The lifetime changes in the cerebellum (**Fig. 5**; expressed as a percentage) are subdivided using the classification from **Fig. 1** and detailed in **Supplementary Data 1**. A positive change corresponds to a longer lifetime in the cerebellum, while a negative change indicates a longer lifetime in the brain cortex. Each data point corresponds to a single protein lifetime and the black lines indicate the mean and the standard error of the mean (SEM) for each group. The results are in agreement with the functional enrichment analysis presented in panel b. Briefly, endocytic proteins and the clathrin endocytosis apparatus are shorter-lived in the cerebellum, while nuclear binding proteins and histones are longer-lived in the cerebellum. **b**, Non redundant gene ontology analysis[11] of the lifetime changes in the cortex compared to the cerebellum. Exo-endocytosis pathways and cell adhesions are more stable in the cortex, while proteins implicated in chromatin assembly and nuclear organization are stabilized in the cerebellum.

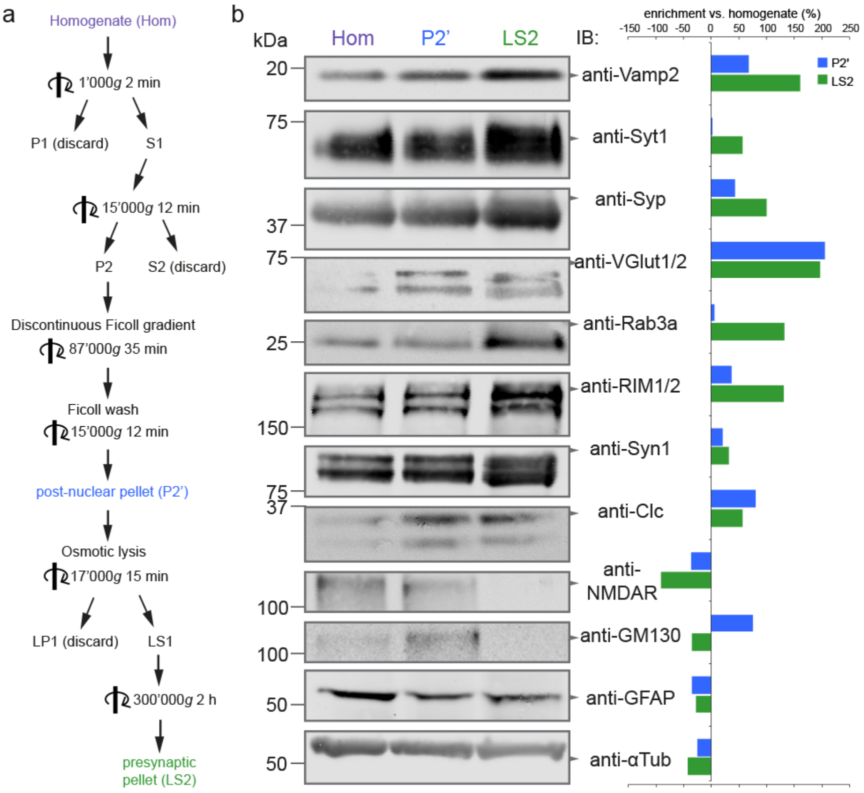

c

Increased lifetimes in synaptic fractions in general (two or more)

Biological process (non redundant; FDR < 0.05)	-log10 p-value
cofactor metabolic process (GO:0051186)	5.80
pyridine-containing compound metabolic process (GO:0072524)	5.65
tricarboxylic acid metabolic process (GO:0072350)	5.00

Cellular component (non redundant; FDR < 0.05)	-log10 p-value
myelin sheath (GO:0043209)	4.80
coated membrane (GO:0048475)	4.58
microtubule (GO:0005874)	3.46
actin cytoskeleton (GO:0015629)	2.89

Molecular function (non redundant; FDR < 0.05)	-log10 p-value
oxidoreductase activity, CH-OH group of donors (GO:0016614)	4.40
oxidoreductase activity, aldehyde group of donors (GO:0016903)	4.32
protein transporter activity (GO:0008565)	3.80

d

Increased lifetimes in the synaptic fractions from the cerebellum

Biological process (non redundant; FDR < 0.05)	-log10 p-value
tricarboxylic acid metabolic process (GO:0072350)	7.91
pyridine-containing compound metabolic process (GO:0072524)	6.40
cofactor metabolic process (GO:0051186)	6.19
sulfur compound metabolic process (GO:0006790)	5.89
generation of precursor metabolites and energy (GO:0006091)	5.28
pyruvate metabolic process (GO:0006090)	4.21

Cellular component (non redundant; FDR < 0.05)	-log10 p-value
mitochondrial matrix (GO:0005759)	5.89
oxidoreductase complex (GO:1990204)	4.74
myelin sheath (GO:0043209)	4.17

Molecular function (non redundant; FDR < 0.05)	-log10 p-value
oxidoreductase activity, aldehyde group of donors (GO:0016903)	4.45

e

Increased lifetimes in the synaptic fractions from the cortex

Cellular component (non redundant; FDR < 0.05)	-log10 p-value
coated membrane (GO:0048475)	5.49
microtubule (GO:0005874)	3.44
membrane region (GO:0098589)	3.02
peptidase complex (GO:1905368)	2.95

Molecular function (non redundant; FDR < 0.05)	-log10 p-value
protein transporter activity (GO:0008565)	4.31
threonine-type peptidase activity (GO:0070003)	4.08

f

Increased lifetimes in SVs

Cellular component (non redundant; FDR < 0.05)	-log10 p-value
actin cytoskeleton (GO:0015629)	3.36
microtubule associated complex (GO:0005875)	3.34

Molecular function (non redundant; FDR < 0.05)	-log10 p-value
motor activity (GO:0003774)	3.75

Supplementary Fig. 21 | The procedure for preparing synaptosomes and synaptic vesicles. a, Graphical view of the centrifugation steps in this procedure. **b**, Western Blots of several synaptic proteins in the important fractions: cortex homogenate (Hom), post-nuclear pellet (P2', containing the synaptosomes), and presynaptic pellet (LS2, containing the crude synaptic vesicles). The blots present four synaptic vesicle proteins (VAMP2, synaptotagmin 1, synaptophysin, and VGLUT1/2), four soluble synaptic proteins (Rab3a, RIM1/2, synapsin 1, and the clathrin light chain, clc), and four control proteins: post-synaptic NMDA receptors, the Golgi marker GM130, the glia marker GFAP, and tubulin. As a graphic aid, the enrichment of different proteins in these fractions, compared to the cortex homogenate, is shown in the graphs on the right. Both postsynaptic and glia markers are de-enriched from the presynaptic fraction. **c-f**, Non redundant gene ontology analysis[11] of the lifetime changes among different fractions. The cellular components that are stabilized in most of the fractions belong to myelin, clathrin coated pits, actin and the microtubule cytoskeleton (**d**). The same trend is observed specifically in the cortex (**e**). In the cerebellum, several mitochondrial proteins are also stabilized in the synaptic fractions. Panel (**g**) analyzes proteins whose lifetimes are not changed in synaptosomes vs. brain homogenates, but are significantly different in synaptic vesicle fractions. This analysis shows that actin and microtubule structures live longer when they are associated to vesicles (**g**).

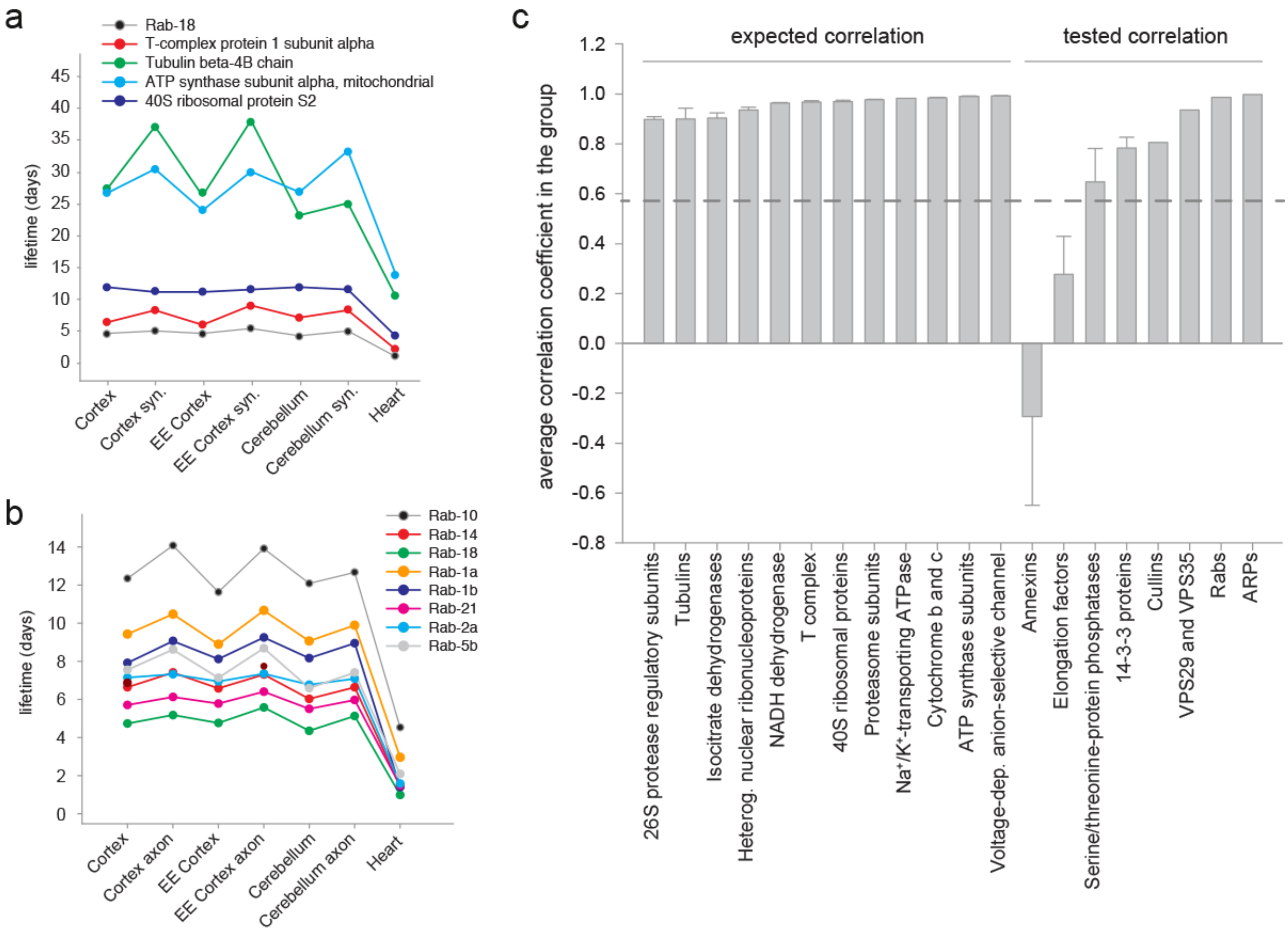

Supplementary Fig. 22 | Protein lifetimes appear to be correlated to their functional interactions, and may reveal functionally related groups of proteins. **a**, The lifetimes of 5 randomly selected proteins, shown in 7 different tissues or compartments. Note the variation in their behaviors across tissues. **b**, A similar plot, for the lifetimes of 9 Rab proteins. Their behaviors are similar. **c**, A quantification of the variation in protein lifetimes across tissues and organs. We calculated the correlation coefficient between the lifetime curves (such as those from panels **a-b**) for the different proteins we detected in all of the 7 tissues or compartments we analyzed. The dashed line indicates the overall correlation of all proteins. The first 12 bars indicate the correlation among functional complexes. The following 8 bars test protein groups whose functional relations are not perfectly understood. Note that some of the correlations are even stronger than those of known functional complexes.

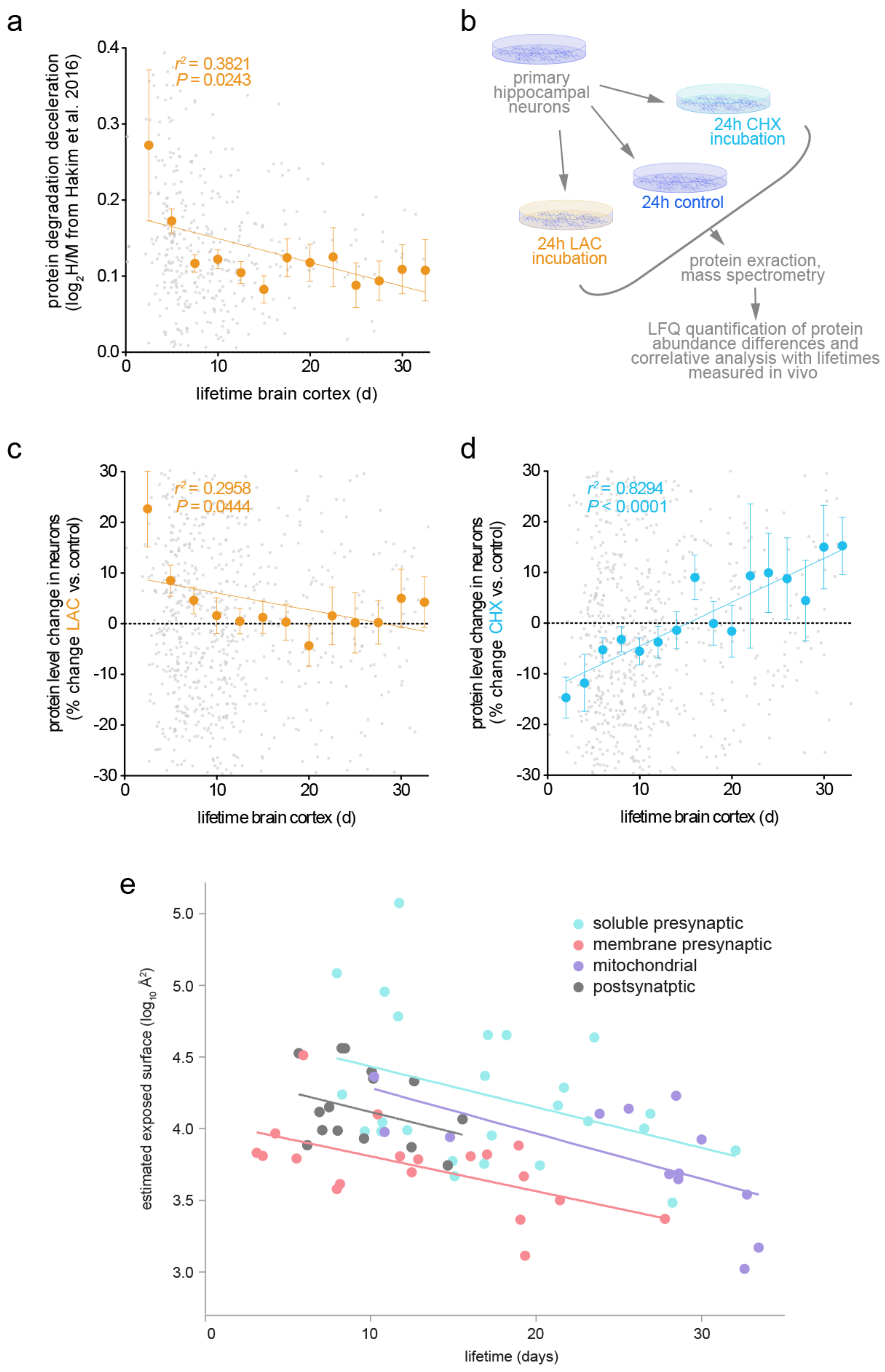

Supplementary Fig. 23 | Processes influencing protein lifetimes. a, We compared lifetimes from the brain cortex with a measurement in which proteasomal protein degradation was inhibited in rat *in vitro*

Supplementary Fig. 23 legend continuing from the previous page:
neuronal cultures[15]. The graph plots the brain lifetimes versus the log_2 of the ratio between the amounts of heavy (H) proteins, obtained in cultures treated with a proteasome inhibitor (Lactacystin), and the medium (M) proteins, obtained in control, untreated cultures (the H and M labels are arbitrary, and derive solely from the experimental design of this publication[15]). Due to the noisy nature of the data, two-day bins are reported (± s.e.m). The graph suggests that the inhibition of protein degradation enables the accumulation mainly of proteins with short lifetimes. **b**, Schematic representation of the experiments performed on primary hippocampal neurons by either blocking the proteasomal function with Lactacystin, or blocking protein translation with Cycloheximide. Primary hippocampal neurons were cultured as previously described [16], were grown *in vitro* for 15 days, and were subjected to the two drugs for 24h. At the end of the experiments proteins were extracted and quantified by MS as described in the methods. This reveled protein level changes in culture that were compared to the lifetimes measured in the brain. **c**, Scatter plot of the protein lifetimes in the cortex *versus* the protein abundance change (expressed as a percentage of the protein abundance ratio between treatment and control). Positive values indicate increase in the protein abundance upon treatment and *vice versa*. The protein degradation block increases the levels of shorter-living proteins, reinforcing the results shown in panel **a**. Also in this case two-day bins are reported (± s.e.m). **d**, As in **a**, for the samples treated with the protein synthesis blocker Cycloheximide. The block of protein translation decreases most significantly the level of short-living proteins, while longer-living proteins appear relatively more abundant, as shown by the positive association, calculated on the binned data. **e**, Analysis of the lifetime of 72 proteins (or protein complexes), versus the size of the exposed surfaces (as detailed in the online methods). We separated the proteins in soluble or membrane presynaptic proteins (cyan and pink, respectively), mitochondria proteins (purple), and postsynaptic proteins (dark gray). Negative correlations between surface and lifetime were determined for all four groups (see line fits).

Supplementary Tables:

	Uniprot ID	Protein	lifetime cortex hom. short pulses (days)	lifetime cortex hom. long pulses (days)	Unlabeled percentile rank	Note	Reported for the first time in this work	Reported in Toyama 2013	Reported in Heo et al 2018
**Nucleus**	Q64523	Histone H2A-2-C	---	*692.83	0.999	Histone	***		
	Q8CGP6	Histone H2A-1-H	28.21	*569.02	0.999	Histone		***	
	P84244	Histone H3.3	103.86	*169.42	0.997	Histone	***		
	P43276	Histone H1.5	45.73	*110.81	0.996	Histone		***	
	P62806	Histone H4	61.68	*87.63	0.992	Histone		***	
	Q6ZWY9	Histone H2B-1-C/E/G	---	*76.53	0.992	Histone		***	
	P43275	Histone H1.1-VAR.3	---	*35.23	0.989	Histone		***	
	Q9D2U9	Histone H2B-3-A	---	*51.57	0.988	Histone	***		
	P43274	Histone H1.4	30.93	*59.76	0.987	Histone	***		
	P43277	Histone H1.3	23.90	*40.54	0.984	Histone		***	
	P10922	Histone H1.0	28.18	*40.77	0.983	Histone		***	
	Q6ZQH8	Nup188 homolog	---	*67.95	0.986	Nuclear pore complex		***	
	Q6PFD9	Nup98-Nup96	---	*36.89	0.988	Nuclear pore complex		***	
	Q9Z0W3	Nup160	---	*35.17	0.986	Nuclear pore complex		***	
	Q99P88	Nup155	---	*23.46	0.953	Nuclear pore complex		***	
	P14733	Lamin-B1	46.98	*86.17	0.992	Nuclear lamina		***	
	P02468	Laminin gamma-1	21.47	*43.71	0.987	Nuclear lamina		***	
	P21619	Lamin-B2	30.65	37.37	0.979	Nuclear lamina		***	
	Q8VDQ8	Sirtuin-2	60.34	*98.00	0.994	--		***	***
	Q60596	DNA repair XRCC1	---	*32.43	0.972	DNA repair enzyme	***		
**Myelin**	Q60771	Claudin-11	133.52	139.17	0.997	Myelin	***		
	Q61885	MOG	117.80	164.41	0.996	Myelin		***	
	Q9D2P8	MBP	79.40	*136.70	0.996	Myelin		***	
	Q8BGN3	Ectonucleotide phosphatase	128.12	157.08	0.994	Probable myelin prot.		***	
	Q922J6	Tetraspanin-2	91.53	89.46	0.993	Probable myelin prot.	***		
	P60202	PLP	102.20	80.36	0.990	Myelin		***	***
	P04370	MBP	82.17	70.09	0.988	Myelin		***	***
	P16330	CNPase	55.64	53.52	0.987	Probable myelin prot.		***	***
	Q9JL62	GLTP	26.65	30.31	0.967	Probable myelin prot.	***		
**Extracellular matrix**	Q62059	Versican core protein	57.77	*687.19	0.999	Extracellular matrix		***	***
	P54320	Tropoelastin	---	*158.60	0.996	Extracellular matrix	***		
	P08122	Collagen alpha-2(IV)	---	*89.17	0.995	Extracellular matrix		***	
	Q9QUP5	Proteoglycan link protein 1	82.13	*112.42	0.994	Extracellular matrix			***
	Q8BYI9	Tenascin-R	38.72	*51.13	0.988	Extracellular matrix			***
	Q04857	Collagen alpha-1(VI)	---	*61.68	0.986	Extracellular matrix		***	
	Q9ESM3	Proteoglycan link protein 2	---	*75.95	0.986	Extracellular matrix	***		
	P11087	Collagen alpha-1(I)	---	*24.72	0.950	Extracellular matrix		***	
**Cytoskeleton**	Q9DCV4	Regulator of MT dynamics	19.45	*60.38	0.990	Microtubule	***		
	Q922F4	Tubulin beta-6	---	*72.54	0.978	Microtubule	***		
	O70318	Epb41l2	24.94	*59.69	0.951	Actin	***		
	Q9CXS4	CENP-V	25.61	*30.23	0.970	centromere	***		
	Q7TMM9	Tubulin beta-2A	32.59	*62.50	0.986	Microtubule			***
	Q9CWF2	Tubulin beta-2B	25.94	37.20	0.980	Microtubule	***		
	P08551	Neurofilament light	33.91	39.37	0.979	Microtubule			***
	P97427	CRMP-1	31.17	32.28	0.969	Microtubule			***
	Q7TSJ2	MAP-6	26.96	31.55	0.966	Microtubule			***
	O08553	CRMP-2	26.13	28.10	0.955	Microtubule			***
	P68372	Tubulin beta-4B	27.20	28.41	0.954	Microtubule			***
	Q9EQF6	CRMP-5	35.23	30.26	0.951	Microtubule			***
	P08553	Neurofilament medium	29.47	36.83	0.977	Neurofilament		***	***
	P46660	Neurofilament-66 (internexin)	35.68	37.61	0.974	Neurofilament			***
	Q810U3	Neurofascin	33.45	39.26	0.974	Neurofilament			***
	P19246	Neurofilament heavy	35.06	32.28	0.962	Neurofilament			***
**Mitochondrion**	Q6PE15	Mycophenolic esterase	23.79	*68.84	0.993	--	***		
	Q8R1S0	Ubiquinone monooxygenase	---	*68.39	0.991	--	***		
	Q8BMF4	PDCE2	36.22	*44.69	0.984	Inner matrix			***
	Q9DCS3	NRBF-1	29.40	35.74	0.978	Inner matrix			***
	Q8BKZ9	Pyruvate dehydrogenase X	33.16	35.17	0.975	Inner matrix			***
	Q9D2G2	OGDC-E2	34.70	36.05	0.974	Inner matrix			***
	Q9D051	PDHE1-B	34.50	35.56	0.972	Inner matrix			***
	Q9CQQ7	ATP synthase F(0) B1	30.66	32.26	0.971	--			***
	Q9CQ69	Cytochrome b-c1/8	29.31	37.67	0.970	Complex III	***		
	Q9EP89	LACTB	27.85	30.63	0.969	--			***
	P62897	Cytochrome c	31.38	31.45	0.966	--			***
	Q9DCX2	ATPase subunit d	32.32	32.89	0.965	Complex V			***
	P35486	PDHE1-A type I	22.48	29.05	0.965	Inner matrix			***
	Q91VR2	ATP synthase gamma	31.50	31.12	0.964	Complex V			***
	Q9D0M3	Cytochrome c1	32.93	31.56	0.962	Complex III			***
	O08749	Dihydrolipoyl dehydrogenase	31.86	31.40	0.962	Inner matrix			***
	Q9DB20	ATP synthase subunit O	30.21	30.64	0.962	Complex V			***
	Q64516	Glycerol kinase	24.73	30.98	0.962	--			***
	P05202	mAspAT	30.34	30.89	0.961	Inner matrix			***
	Q9CZ13	Cytochrome b-c1	30.57	31.54	0.959	Complex III			***
	Q3TC72	Fumarylacetoacetate hydro.	25.80	30.17	0.959	--			***
	Q9CZR3	TOM40B	28.70	32.18	0.959	--	***		
	Q9CPQ8	ATP synthase g	30.11	30.64	0.958	Complex V			***
	P67778	Prohibitin-1	30.11	30.32	0.958	Inner membrane			***
	P08249	Malate dehydrogenase	29.71	29.43	0.956	Inner matrix			***
	Q03265	ATP synthase subunit alpha	26.63	29.15	0.956	Complex V			***
	Q91V61	Sideroflexin-3	30.02	28.93	0.956	Inner membrane			***
	Q9CR68	Cytochrome b-c1	25.71	27.84	0.956	Complex III			***
	Q9CZP5	BCS1	---	*29.71	0.955	--	***		
	P62880	G protein subunit beta-2	27.51	29.46	0.955	--			***
	Q06185	ATP synthase subunit e	30.27	28.41	0.954	Complex V			***
	Q9DCZ4	MICOS (Mic26)	26.97	30.59	0.954	Inner membrane			***
	Q99JR1	Sideroflexin-1	29.94	33.05	0.954	--			***
	Q9D6R2	Isocitrate dehydrogenase	27.17	26.74	0.953	Inner matrix			***
	Q8BGH2	Samm50	29.20	29.36	0.953	Outer membrane			***
	Q9CZU6	Citrate synthase	28.86	28.39	0.952	Inner matrix			***
	Q9D855	Cytochrome b-c1/7	30.40	31.13	0.952	Complex III			***
	P09671	Superoxide dismutase	29.09	28.13	0.952	--			***
	P63038	Hsp60	25.74	27.15	0.952	Inner matrix			***
	O35129	Prohibitin-2	29.35	30.27	0.960	Inner membrane			***
	O55125	NipSnap1	33.40	37.49	0.957	Also in synapse			***
**Other**	P06837	Neuromodulin (GAP-43)	34.60	37.62	0.977	Signalling regulator			***
	P55937	Golgin-160	56.23	48.39	0.981	Golgi structural	***		
	Q91XV3	NAP-22	28.36	36.43	0.972	Signalling regulator			***
	P31324	Prkar2b	27.19	28.64	0.961	Signalling regulator			***
	O70443	Gz-alpha	35.84	44.26	0.981	Signalling regulator			***
	P62874	Transducin beta chain 1	29.19	28.86	0.956	Signalling regulator			***
	O35526	Syntaxin-1A	31.48	30.53	0.957	Presynaptic protein			***

Supplementary Table 1 | List of long-living proteins, divided by subcellular localization. The list contains the proteins that are in the 95th percentile of most stable proteins identified in the brain cortex homogenate sample from this study. We also report the position of the specific protein with respect of the entire proteome as “Unlabeled percentile rank”. This is close to the type of measurement performed in previous studies of ELLPs, in which this was the only major parameter measured. In the last three columns we also indicate long living proteins that have been reported in previous works[8,17].

a

	lifetime change EE hom. vs. ctrl	adj. *P*-value homogenate	lifetime change EE syn. vs. ctrl	adj. *P*-value synaptosomes
L1-CAM	-32.06	1.40E-07	-30.88	2.20E-05
OPA1	-26.99	<1E-10	-14.83	9.80E-10
HADNB	-21.46	<1E-10	-13.80	3.30E-10
Neurexin-4 (Cntnap1)	-15.03	<1E-10	-15.67	<1E-10
VDAC2	-15.12	<1E-10	-11.87	<1E-10
PSD3	-15.86	<1E-10	-15.97	2.00E-09
Neurofilament-M	-15.17	<1E-10	-11.79	1.20E-06
SRC	-17.35	<1E-10	-11.26	<1E-10
Mitofilin	-12.54	<1E-10	-9.93	6.10E-09
GPDH-M (Gpd2)	-15.57	<1E-10	-8.82	7.70E-06
CASK	-15.42	<1E-10	-7.65	1.00E-05
Septin-7	-8.80	1.40E-08	-17.02	<1E-10
HADNA	-7.96	<1E-10	-7.44	8.10E-09
SAP97 (Dlg1)	-21.56	2.4-09	-10.14	4.78E-08
Neuroplastin	-7.36	1.3-06	-8.07	2.80E-06
Neuronal RasGAP (SynGap1)	-9.19	5.9-09	-4.87	0.0008
Glut. dehydrog. (Glud1)	-6.94	<1E-10	-5.65	0.0003
Synapsin-1	-8.85	1.3-06	-9.34	0.0001
Myelin proteolipid (PLP1)	4.87	9.8-06	8.93	<1E-10
Claudin-11 (Cldn11)	14.79	<1E-10	21.98	<1E-10

b

String analysis Biological Process	FDR
nervous system development (GO:0007399)	0.0064
single-organism cellular process (GO:0044763)	0.0072
cellular process (GO:0009987)	0.0114
single-organism process (GO:0044699)	0.0169
regulation of neuronal synaptic plasticity (GO:0048168)	0.0193
negative regulation of signal transduction (GO:1902532)	0.0251
anatomical structure development (GO:0048856)	0.0363
small molecule catabolic process (GO:0044282)	0.0408
system development (GO:0048731)	0.0408

String analysis Molecular Function	FDR
acyl-CoA dehydrogenase activity (GO:0016509)	0.003
acetyl-CoA C-acyltransferase activity (GO:0003988)	0.0279

String analysis KEGG Pathways	FDR
Fatty acid elongation (62)	0.0293
Cell adhesion molecules (CAMs) (4514)	0.0293
Tight junction (4530)	0.0293
Fatty acid degradation (71)	0.0418
Valine, leucine and isoleucine degradation (280)	0.0418
Fatty acid metabolism (1212)	0.0418

Panther pathway analysis for all significantly changed lifetimes	FDR
PI3 kinase pathway (P00048)	0.0155
Enkephalin release (P05913)	0.0155
Metabotropic glutamate receptor group II pathway (P00040)	0.0155
Heterotrimeric G-protein signaling (P00026)	0.0337
5HT1 type receptor mediated signaling pathway (P04373)	0.0391
Muscarinic acetylcholine receptor 2 and 4 signaling pathway (P00043)	0.0444

String analysis Cellular Component	FDR
myelin sheath (GO:0043209)	4E-11
mitochondrial inner membrane (GO:0005743)	3E-07
synapse (GO:0045202)	3E-07
mitochondrial envelope (GO:0005740)	3E-06
membrane (GO:0016020)	1E-05
neuron part (GO:0097458)	1E-05
synapse part (GO:0044456)	2E-05
neuron projection (GO:0043005)	2E-05
cell projection part (GO:0044463)	2E-05
axon part (GO:0033267)	3E-05
axon (GO:0030424)	4E-05
mitochondrial fatty acid beta-oxid. complex (GO:0016507)	7E-05
mitochondrial part (GO:0044429)	0.0002
cell projection (GO:0042995)	0.0002
whole membrane (GO:0098805)	0.0003
mitochondrial nucleoid (GO:0042645)	0.0004
plasma membrane part (GO:0044459)	0.0005
membrane region (GO:0098589)	0.0007
postsynaptic density (GO:0014069)	0.001
cell junction (GO:0030054)	0.0011
plasma membrane region (GO:0098590)	0.0024
synaptic membrane (GO:0097060)	0.0027
plasma membrane (GO:0005886)	0.003
membrane part (GO:0044425)	0.0045
axon terminus (GO:0043679)	0.0046
organelle membrane (GO:0031090)	0.0053
postsynapse (GO:0098794)	0.0078
mitochondrial outer membrane (GO:0005741)	0.0092

Supplementary Table 2 | Protein lifetime differences upon chronic environmental enrichment. a, A precise analysis of the proteins with significantly different lifetimes identifies synaptic components that are turned over at a higher speed following environmental enrichment, such as the presynaptic adhesion molecule Neurexin-4, the scaffold molecule CASK, the phosphoprotein synapsin-1 and the neuronal RasGAP SynGAP1 (See also **Fig. 7**). Some mitochondrial components implicated in the metabolism of glutamate and acetyl-CoA are also turned over at a higher speed following environmental enrichment. On the contrary, two myelin components (PLP1 and Claudin 11) are stabilized upon environmental enrichment. **b**, Details of the string analysis of the 20 common proteins presented in **Fig. 7**. Three functional protein clusters are changed upon environmental enrichment. The analysis indicates that the most important differences between these two cohorts of mice are at the level of myelin, the mitochondrial inner membrane and the synapse.

a

GO categories for relatively shorter living in cultured neurons vs. brain cortex (<15th percentile; non redundant; FDR < 0.05)	$-\log_{10}$ p-value
presynapse (GO:0098793)	9.65
myelin sheath (GO:0043209)	8.64
transport vesicle (GO:0030133)	7.48
secretory vesicle (GO:0099503)	6.68
inorganic cation TM transporter activity (GO:0022890)	6.19
active transmembrane transporter activity (GO:0022804)	6.10
axon (GO:0030424)	5.92
neurotransmitter transport (GO:0006836)	5.88
plasma membrane protein complex (GO:0098797)	5.58
hydrogen transport (GO:0006818)	5.51
regulation of neurotransmitter levels (GO:0001505)	5.31
monovalent inorganic cation transport (GO:0015672)	5.00
neuron projection terminus (GO:0044306)	5.00
synaptic vesicle cycle (GO:0099504)	4.64
membrane region (GO:0098589)	4.39
ATP hydrolysis coupled TM transport (GO:0090662)	4.23

GO categories for relatively longer living in cultured neurons vs. brain cortex (>85th percentile; non redundant; FDR < 0.05)	$-\log_{10}$ p-value
peptidase complex (GO:1905368)	13.3
ubiquitin-dependent catabolic process (GO:0006511)	5.23
transcription factor binding (GO:0008134)	4.62
endopeptidase activity (GO:0004175)	4.61
threonine-type peptidase activity (GO:0070003)	4.30
proteasomal protein catabolic process (GO:0010498)	4.27
DNA-templated transcription, initiation (GO:0006352)	3.68

b

GO categories for relatively shorter living in heart vs. brain cortex (<15th percentile; non redundant; FDR < 0.05)	$-\log_{10}$ p-value
GTPase activity (GO:0003924)	7.50
guanyl nucleotide binding (GO:0019001)	6.19
membrane region (GO:0098589)	5.44
small GTPase mediated signal transduction (GO:0007264)	4.93
midbody (GO:0030496)	4.69
cytokinesis (GO:0000910)	4.31
vesicle membrane (GO:0012506)	4.09
plasma membrane protein complex (GO:0098797)	3.81
coated membrane (GO:0048475)	3.78
extrinsic component of membrane (GO:0019898)	3.66
positive regulation of component biogenesis (GO:0044089)	3.65
Golgi membrane (GO:0000139)	3.56
coated vesicle (GO:0030135)	2.70
organelle subcompartment (GO:0031984)	2.70
microtubule (GO:0005874)	2.51
cell leading edge (GO:0031252)	2.51

GO categories for relatively longer living in heart vs. brain cortex (>85th percentile; non redundant; FDR < 0.05)	$-\log_{10}$ p-value
fatty acid metabolic process (GO:0006631)	7.68
oxidoreductase activity (GO:0016627)	6.88
lipid catabolic process (GO:0016042)	6.47
lipid modification (GO:0030258)	6.44
small molecule catabolic process (GO:0044282)	5.72
lipid homeostasis (GO:0055088)	5.66
electron carrier activity (GO:0009055)	5.08
protein-DNA complex (GO:0032993)	4.76
sulfur compound binding (GO:1901681)	3.91
DNA packaging complex (GO:0044815)	3.53
cofactor binding (GO:0048037)	3.40

c

GO categories for relatively shorter living in muscle vs. brain cortex (<15th percentile; non redundant; FDR < 0.05)	$-\log_{10}$ p-value
DNA biosynthetic process (GO:0071897)	4.26
positive regulation of transferase activity (GO:0051347)	3.69
cellular protein complex assembly (GO:0043623)	3.46
cellular response to nitrogen compound (GO:1901699)	3.29
negative regulation of organelle organization (GO:0010639)	3.20
regulation of DNA metabolic process (GO:0051052)	3.20

GO categories for relatively longer living in muscle vs. brain cortex (>85th percentile; non redundant; FDR < 0.05)	$-\log_{10}$ p-value
nucleoside diphosphate metabolic process (GO:0009132)	5.24
nucleotide phosphorylation (GO:0046939)	3.67

Supplementary Table 3 | Gene ontology analysis of lifetime changes across different cells or tissues Associated to Fig. 8. a, Analysis of the lifetimes in the cortex homogenate versus the lifetimes of primary rat neurons (published elsewhere[7]). **b**, Analysis of the lifetimes in the cortex homogenate versus the lifetimes in heart samples. **c**, Analysis of the lifetimes in the cortex homogenate versus the lifetimes of skeletal muscle (gastrocnemius). Please note that all these analysis take into account the average differences, and point to the "especially short-lived" or "especially long-lived" proteins.

Target	Antibody/company	Origin	Concentration
VAMP2	SySy 104211 or 69.1	mouse monoclonal	1:1000
Synaptotagmin1	SySy 105011	mouse monoclonal	1:1000
Synaptophysin	SySy 101011 or 7.2	guinea pig	1:1000
VGlut1/2	SySy 135503	rabbit polyclonal	1:1000
Rab3a	BD Clone 9 - 610379	mouse monoclonal	1:1000
Rim1/2	SySy 140003	rabbit polyclonal	1:500
Synapsin	clone M10.22 from Jahn R	mouse monoclonal	1:1000
clathrin light chain	SySy 113001	mouse monoclonal	1:5000
NMDA receptor	BD clone 54.2 -	mouse monoclonal	1:1000
GM130	BD 610822	mouse monoclonal	1:1000
GFAP	NB300-141	rabbit polyclonal	1:1000
alpha-Tubulin	SySy 302203	rabbit polyclonal	1:2000

Supplementary Table 4 | Antibodies used in this study for WB

Supplementary References

1. Spiess, A. N. & Neumeyer, N. An evaluation of R2as an inadequate measure for nonlinear models in pharmacological and biochemical research: A Monte Carlo approach. *BMC Pharmacol.* **10,** 1–11 (2010).

2. Soriano, P. Generalized lacZ expression with the ROSA26 Cre reporter strain. *Nat. Genet.* **21,** 70–1 (1999).

3. Erdmann, G., Schütz, G. & Berger, S. Inducible gene inactivation in neurons of the adult mouse forebrain. *BMC Neurosci.* **8,** 63 (2007).

4. John, A. M. & Bell, J. M. Amino acid requirements of the growing mouse. *J. Nutr.* **106,** 1361–7 (1976).

5. Price, J. C., Guan, S., Burlingame, A., Prusiner, S. B. & Ghaemmaghami, S. Analysis of proteome dynamics in the mouse brain. *Proc. Natl. Acad. Sci. U. S. A.* **107,** 14508–13 (2010).

6. Dörrbaum, A. R., Kochen, L., Langer, J. D. & Schuman, E. M. Local and global influences on protein turnover in neurons and glia. *Elife* **7,** 1–24 (2018).

7. Mathieson, T. *et al.* Systematic analysis of protein turnover in primary cells. *Nat. Commun.* **9,** 1–10 (2018).

8. Heo, S. *et al.* Identification of long-lived synaptic proteins by proteomic analysis of synaptosome protein turnover. *Proc. Natl. Acad. Sci.* 201720956 (2018). doi:10.1073/pnas.1720956115

9. Cohen, L. L. D. *et al.* Metabolic turnover of synaptic proteins: kinetics, interdependencies and implications for synaptic maintenance. *PLoS One* **8,** e63191 (2013).

10. Bezdek, J. C., Ehrlich, R. & Full, W. FCM: The fuzzy c-means clustering algorithm. *Comput. Geosci.* **10,** 191–203 (1984).

11. Wang, J., Vasaikar, S., Shi, Z., Greer, M. & Zhang, B. WebGestalt 2017: a more comprehensive, powerful, flexible and interactive gene set enrichment analysis toolkit. *Nucleic Acids Res.* **45,** 1–8 (2017).

12. Szklarczyk, D. *et al.* The STRING database in 2017: quality-controlled protein-protein association networks, made broadly accessible. *Nucleic Acids Res.* **45,** D362–D368 (2017).

13. Sharma, K. *et al.* Cell type- and brain region-resolved mouse brain proteome. *Nat. Neurosci.* **18,** 1819–31 (2015).

14. Boyken, J. *et al.* Molecular profiling of synaptic vesicle docking sites reveals novel proteins but few differences between glutamatergic and GABAergic synapses. *Neuron* **78,** 285–297 (2013).

15. Hakim, V., Cohen, L. D., Zuchman, R., Ziv, T. & Ziv, N. E. The effects of proteasomal inhibition on synaptic proteostasis. *EMBO J.* **9,** e201593594 (2016).

16. Kaech, S. & Banker, G. Culturing hippocampal neurons. *Nat. Protoc.* **1,** 2406–15 (2006).

17. Toyama, B. H. *et al.* Identification of long-lived proteins reveals exceptional stability of essential cellular structures. *Cell* **154,** 971–82 (2013).
