# Supplementary material for: Precisely measured protein lifetimes in the mouse brain reveal differences across tissues and subcellular fractions
Source: Nat Commun. 2018 Oct 12;9:4230. doi: 10.1038/s41467-018-06519-0 (PMC6185916; doi:10.1038/s41467-018-06519-0)
Supplement: Supplementary file 2 — Description of Additional Supplementary Files [file 41467_2018_6519_MOESM2_ESM.pdf]

Description of Additional Supplementary Files

File Name: Supplementary Data 1

Description: Protein lifetime values, categorization and protein information generated in this work. For a detailed description of Supplementary Data 1 content, please refer to the first sheet of the file.
